# Assessment of RainDrop BS-seq as a method for large-scale, targeted bisulfite sequencing

**DOI:** 10.4161/epi.28041

**Published:** 2014-02-11

**Authors:** Dirk S Paul, Paul Guilhamon, Anna Karpathakis, Lee M Butcher, Christina Thirlwell, Andrew Feber, Stephan Beck

**Affiliations:** UCL Cancer Institute; University College London; London, United Kingdom

**Keywords:** DNA methylation, EWAS, RainDrop BS-seq, epigenetic biomarker, microdroplet PCR, targeted bisulfite sequencing

## Abstract

We present a systematic assessment of RainDrop BS-seq, a novel method for large-scale, targeted bisulfite sequencing using microdroplet-based PCR amplification coupled with next-generation sequencing. We compared DNA methylation levels at 498 target loci (1001 PCR amplicons) in human whole blood, osteosarcoma cells and an archived tumor tissue sample. We assessed the ability of RainDrop BS-seq to accurately measure DNA methylation over a range of DNA quantities (from 10 to 1500 ng), both with and without whole-genome amplification (WGA) following bisulfite conversion. DNA methylation profiles generated using at least 100 ng correlated well (median *R* = 0.92) with those generated on Illumina Infinium HumanMethylation450 BeadChips, currently the platform of choice for epigenome-wide association studies (EWAS). WGA allowed for testing of samples with a starting DNA amount of 10 and 50 ng, although a reduced correlation was observed (median *R* = 0.79). We conclude that RainDrop BS-seq is suitable for measuring DNA methylation levels using nanogram quantities of DNA, and can be used to study candidate epigenetic biomarker loci in an accurate and high-throughput manner, paving the way for its application to routine clinical diagnostics.

## Introduction

Variation in DNA methylation has been associated with predisposition, progression and response to treatment of a broad range of clinical conditions such as cancer and autoimmune diseases.^1^ Due to its chemical stability, cytosine methylation (5mC) in the CpG dinucleotide context can be reliably detected from many different sources such as body fluids (e.g., blood and urine), buccal mucosa, as well as fresh and archived tissues.^2^

Technological advances in next-generation sequencing have enabled studies of DNA methylation variation on a genome-wide scale and with single CpG resolution. Such methods include whole-genome bisulfite sequencing (WGBS), reduced representation bisulfite sequencing (RRBS) and methylCRF, which combines methylated DNA immunoprecipitation (MeDIP-seq) and methylation-sensitive restriction enzyme sequencing (MRE-seq). However, these techniques are still prohibitively costly, resource-intensive and inefficient when assessing large numbers of samples.^2^^-^^4^

Microarray-based applications, foremost the Illumina Infinium HumanMethylation450 BeadChip (“450K array”), interrogate only a predefined fraction of the DNA methylome but at reduced cost.^5^ Indeed, 450K arrays have proven to be the method of choice for epigenome-wide association studies (EWAS), in which disease-associated DNA methylation variable positions are identified in case-control sample cohorts.^6^^,^^7^ Several techniques have been established to map DNA methylation with single CpG resolution at a selected subset of genomic regions of interest (e.g., to validate EWAS signals), including pyrosequencing, Sanger sequencing and high-resolution melting curve analysis. However, most of these have not been optimized for high-throughput applications.^8^^,^^9^

Emerging techniques that utilize next-generation DNA sequencing platforms are particularly promising for the large-scale, targeted bisulfite sequencing of genomic regions of interest. For example, the enrichment of selected bisulfite-converted loci has been achieved by array-based^10^ and solution-based^11^ hybridization capture as well as bisulfite padlock probes,^12^^-^^14^ combined with next-generation sequencing of the captured loci. Recently, RainDance Technologies developed an alternative and fully integrated enrichment system using microdroplet PCR that can also be coupled to next-generation sequencing platforms.^15^ The encapsulation of distinct PCR reactions in microdroplets enables the sensitive, specific and simultaneous amplification of up to 20 000 and 4000 target loci using unconverted and bisulfite-converted genomic DNA, respectively. Its application for targeted bisulfite sequencing was first reported in 2011 by Komori et al.^16^ to analyze over 77000 CpGs in primary human CD4^+^ T cells, and Herrmann et al.^17^ to track hepitype evolution in tumors. In 2013, Guilhamon et al. refined the method into RainDrop BS-seq and used it to validate a hypermethylation phenotype in isocitrate dehydrogenase (IDH) mutant chondrosarcoma.^18^

The key limitation of RainDrop BS-seq is the requirement of large quantities of DNA as starting material (i.e., 4 μg before bisulfite conversion), impeding its application to clinical samples such as tumor fragments obtained by laser capture microdissection, fluorescence-sorted or stem cell populations, for which often only nanogram quantities of DNA are available. To this end, Bundo et al. recently evaluated the reproducibility of multiple displacement amplification (MDA)-based whole-genome amplification (WGA) of bisulfite-treated DNA compared with unamplified samples. The results indicated reasonable correlation between amplified and unamplified DNA methylation profiles, i.e., *R_s_* = 0.86 and *R_s_* = 0.93 for 10 and 50 ng, respectively.^19^ While this assessment concerned methylation profiles generated on 450K arrays, it showed the potential benefit of applying WGA to samples of limited DNA quantity.

Here, we present a systematic assessment of RainDrop BS-seq as a method for large-scale, targeted bisulfite sequencing using a wide range of starting DNA quantity and quality, different cell types and application of MDA-based WGA of bisulfite-converted DNA. In addition, we correlate the DNA methylation profiles generated with those obtained on 450K arrays to validate RainDrop BS-seq as a suitable method for EWAS validation and replication experiments.

## Results and Discussion

### Sample preparation

We isolated human genomic DNA from whole blood, the osteosarcoma cell line 143B, and a formalin-fixed paraffin-embedded (FFPE) tumor sample (Methods section). We applied bisulfite conversion to 1500, 1000, 250, and 100 ng of genomic DNA from each sample. In parallel, we bisulfite-treated and subsequently applied MDA-based WGA to another 250, 100, 50, and 10 ng of genomic DNA from each sample.

### Primer panel design

We designed two independent primer panels to measure methylation levels at selected genomic regions. Each interrogated target locus was centered on at least one specific CpG site that is also present on the 450K array platform. First, we targeted 27 loci (500 PCR amplicons) containing a total of 212 CpG sites that are represented on the 450K array (Table S1A). These loci were recently identified as being differentially methylated in central chondrosarcoma with and without IDH mutations.^18^ The sarcoma panel (“SC panel”) was used with eight different starting DNA amounts from each of whole blood (BL), osteosarcoma cells (SCL) and an FFPE sample (see above). Second, we targeted an additional 462 candidate epigenetic biomarker loci (501 PCR amplicons) containing 778 of the 450K CpG sites (Table S1B). These loci were selected as part of an ongoing epigenetic biomarker validation experiment (Bock et al., in preparation). Here, the biomarker panel (“BM panel”) was applied to BL and SCL samples only. Both primer panels were devised using a primer design algorithm described in detail by Komori et al.^16^ The primer design pipeline utilizes the Primer3 software as well as electronic PCR to restrict off-target amplification. All steps of the primer panel generation were performed by RainDance Technologies.

### RainDrop BS-seq

Bisulfite-treated DNA was used as template for the microdroplet-based PCR amplification reaction with a RainDance ThunderStorm system (Fig. 1). After destabilization of the microdroplet PCR, the purified PCR amplicons were analyzed on an Agilent 2100 Bioanalyzer. Samples that did not undergo WGA (“unamplified samples”) demonstrated a DNA quantity-dependent enrichment at the expected fragment size range (Fig. S1A–E). While WGA samples also showed enrichment at the expected size range, additional unspecific, high-molecular artifacts were observed (Fig. S1A–E). The latter may have arisen through incomplete bisulfite conversion, after which high-molecular DNA fragments get preferably amplified in MDA-based WGA.^20^ However, such high-molecular fragments are unlikely to serve as templates for the microdroplet PCR due to steric hindrance within the droplets, and thus do not carry the incorporated sequence tag crucial for the subsequent PCR step (Fig. 1). The amplified PCR fragments then served as templates for a second (“universal”) PCR step to incorporate the sequencing adapters and unique barcodes to enable Illumina DNA sequencing. Following purification, PCR products were again analyzed on an Agilent 2100 Bioanalyzer. Both unamplified and WGA samples demonstrated successful integration of Illumina adapters (Fig. S2A–E). The samples were combined in equimolar concentrations, and the library sequenced on an Illumina MiSeq.

**Figure:**
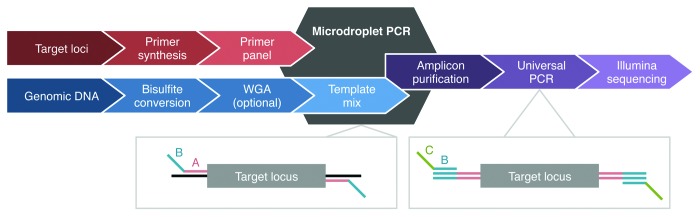
**Figure 1.** RainDrop BS-seq workflow. RainDrop BS-seq allows targeted, high-throughput bisulfite sequencing using the microdroplet-based PCR amplification system developed by RainDance Technologies. The custom primer panel for the genomic regions of choice is prepared by RainDance Technologies. The workflow comprises the following key steps: (1) bisulfite conversion of genomic templates; (2) merger of picoliter-volume droplets of bisulfite-treated templates with pre-made primer pair droplets (primer panel) on microfluidic chips; (3) pooled, thermal cycling of the PCR reactions (microdroplet PCR); (4) destabilization of droplets to release the PCR products; (5) purification of PCR products using magnetic beads; (6) incorporation of DNA sequencing barcodes through standard PCR (universal PCR), followed by purification of the PCR products and Illumina sequencing. The workflow comprises a two-step tailed primer strategy. First, target sequences (**A**) are amplified with tailed primers containing a partial Illumina adapter sequence (**B**). Second, the remainder of the Illumina adapter sequence as well as a unique barcode sequence (**C**) is added to enable multiplexing of samples for next-generation DNA sequencing.

### Targeted DNA sequencing statistics

We analyzed the sequencing data as described in the Methods section, with sequencing statistics shown in Table S2A and B. For the SC panel (Table S2A), the fraction of aligned sequencing reads mapping to the target amplicons ranged between 93.0–97.4% for unamplified BL and SCL samples. For unamplified FFPE samples, the fraction ranged between 81.4–84.5%. Taken together, this suggests a relatively small impact of starting DNA amount (between 100–1500 ng) on read mapping specificity. WGA did not improve the target coverage in comparison to unamplified samples of the same quantity of DNA material, i.e., 100 and 250 ng (Table S2A and B). Samples with a starting DNA amount of 10 and 50 ng attained a target coverage of at least 59.8%. Note that samples with DNA quantities of 10 and 50 ng were only processed following WGA. Target coverage profiles obtained for the BM panel followed a similar read coverage pattern as for the SC panel (Table S2B).

Figure 2 gives an overview of the average read coverage per individual PCR amplicon for the two primer panels. Notably, only one PCR amplicon across the two panels failed to amplify in any of the samples tested. For the BM panel, only the samples with a starting material of 10 and 50 ng had a PCR amplification success rate of less than 95% (Fig. 2A). For the SC panel, this was also the case for the 10 ng samples in BL, and 10 and 50 ng samples in FFPE (Fig. 2B). In summary, these results indicate a robust performance overall of the custom primer panel applied to different tissue types and DNA quantities, however it is less reliable when applied to WGA-treated DNA.

**Figure:**
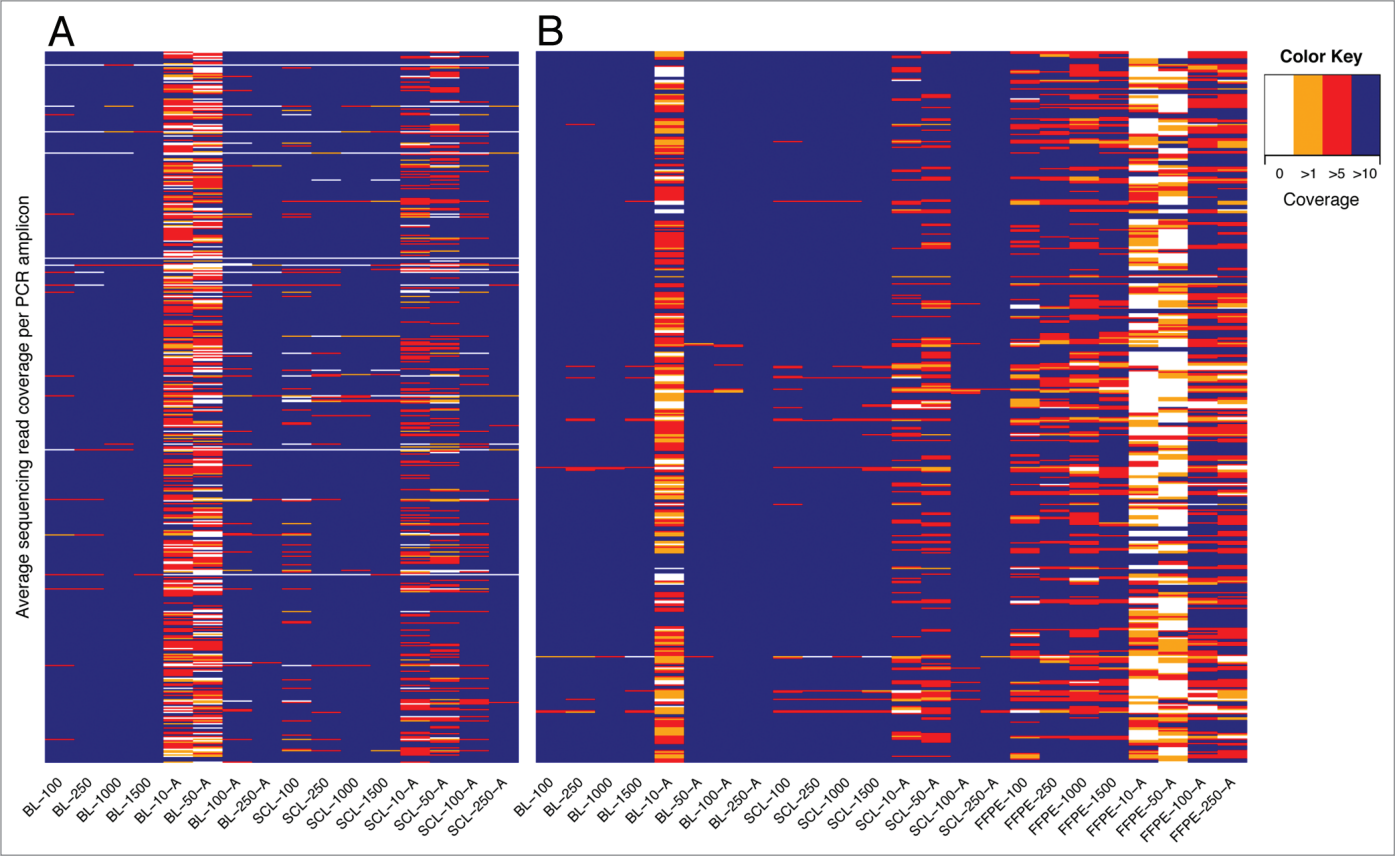
**Figure 2.** Average per-base sequencing read coverage across PCR amplicons. The heatmaps show the average per-base read coverage across the ~500 PCR amplicons using the (**A**) BM primer panel and (**B**) SC panel. The color scheme indicates different levels of read coverage, i.e., 0, 1, 5 and 10. PCR amplicons were mostly covered with at least five sequencing reads across all samples other than those with a starting DNA material of 10 and 50 ng.

Next, we assessed the fraction of target bases with a coverage ranging from 1 to 1000 across the 462 and 27 target loci of the BM (Fig. 3A and B) and SC panel (Fig. S3A–C), respectively. Across both primer panels, and in line with the data shown in Figure 2, the 10 and 50 ng WGA samples showed markedly reduced coverage levels compared with all other samples. In order to assess the effect of different starting DNA quantities on target coverage, we calculated the standard deviation between samples at differing coverage levels. For the BM panel, with a 5-fold coverage the standard deviation of coverage values across unamplified BL and SCL samples was σ = 0.013 and σ = 0.036, respectively, suggesting little effect of input quantity. In contrast, the standard deviation was σ = 0.185 and σ = 0.289, respectively, with a coverage of 100 sequencing reads (Fig. 3A and B). We confirmed this trend using the SC panel, although smaller standard deviation values were observed with a coverage of 100 sequencing reads (Fig. S3A and B). This was likely due to the increased tiling (i.e., multiple, overlapping PCR amplicons) at the target loci. These results indicate a negligible effect of different starting DNA amounts of unamplified samples on the fraction of target bases with a coverage of five sequencing reads, a coverage we consider sufficient to confidently call DNA methylation levels for comparison to 450K arrays, e.g., in EWAS validation experiments (Fig. S4).

**Figure:**
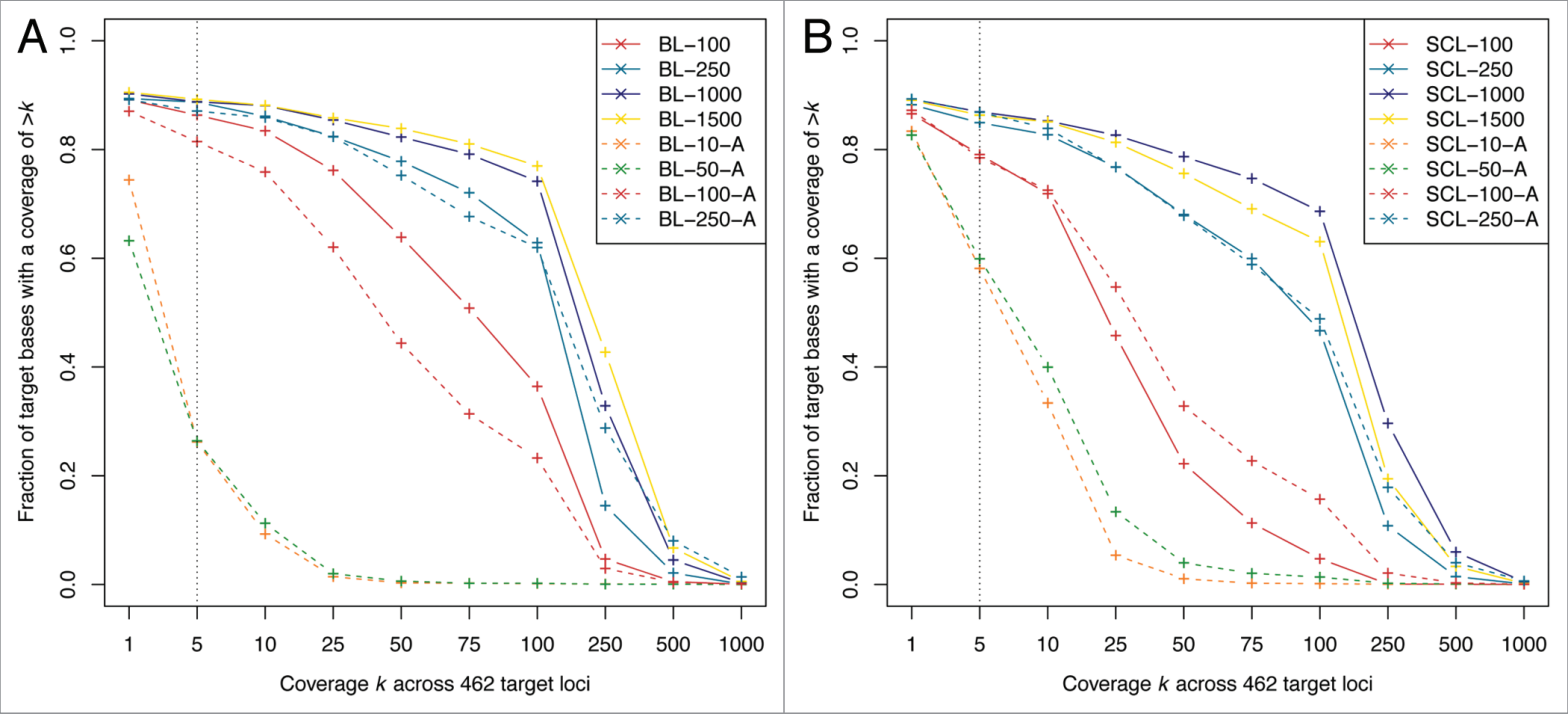
**Figure 3.** Fraction of target bases with a coverage ranging from 1 to 1000 across target loci. The plots show the fraction of target bases with a coverage ranging from 1 to 1000 at 462 target loci across (**A**) BL and (**B**) SCL samples, assessed using the BM panel. Note that the *x*-axis is nonlinear. A coverage of five sequencing reads is indicated with a dotted line. With this coverage level, 80% of target bases were usually covered across all samples other than those with an input material of 10 and 50 ng. A reduction of the starting DNA amount resulted in decreased coverage overall at the target loci.

Finally, for FFPE samples, the fraction of target bases with a coverage of five sequencing reads was ~50%, suggesting impeded PCR amplification (Fig. S3C). We conclude that after bisulfite conversion of the already degraded DNA of the FFPE sample through the fixation and storage process, the DNA templates may be too short for microdroplet-based PCR amplification using a primer panel with an average PCR amplicon length of ~200 bp (Fig. S1C). In future protocols, PCR amplification may be enhanced through the incorporation of locked nucleic acid (LNA) residues into primer sequences, which demonstrate increased affinity and specificity for very short DNA templates.^21^

### Assessment of DNA methylation levels

We then extracted the methylation levels within the CpG context, applying a cut-off of at least five sequencing reads covering the PCR amplicon (corresponding to a quantitative resolution of 20%; Fig. S4). For the BM panel, we correlated the methylation levels at 497 and 1581 CpG sites across BL and SCL samples, respectively (Fig. 4A and B). Globally, we found that the methylation profiles were strongly correlated in unamplified samples (median *R* = 0.98). The strong correlation was confirmed (median *R* = 0.97) at 240, 774 and 13 CpG sites across BL, SCL and FFPE samples using the SC primer panel (Fig. S5A–C). These findings are in agreement with the notion that the fraction of target bases with a coverage of five sequencing reads is similar (see above and Fig. 3A and B).

**Figure:**
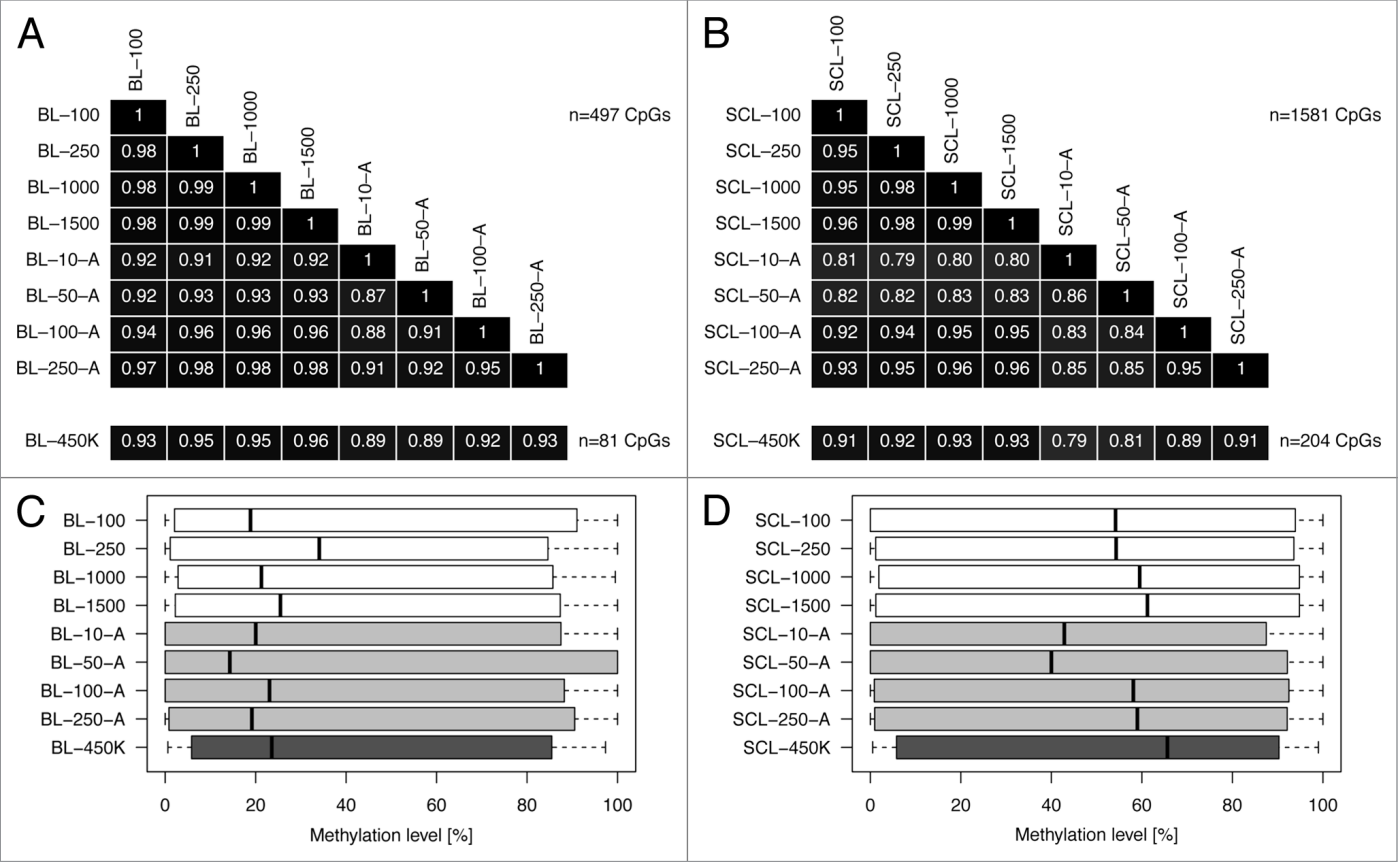
**Figure 4.** Correlation of DNA methylation levels and comparison to 450K arrays. The heatmaps show matrices of Pearson’s correlation coefficients across (**A**) BL samples (n = 497 CpG sites) and (**B**) SCL samples (n = 1581 CpG sites), assessed using the BM panel. The DNA methylation levels of unamplified samples with a starting DNA amount between 100 ng and 1500 ng were strongly correlated (median *R* = 0.98). Further, we correlated a subset of these CpG sites, i.e., n = 81 and n = 204 CpGs across BL and SCL samples, that are represented on the 450K array. We found a strong correlation (*R* ≥ 0.91) for unamplified samples and a moderate correlation (*R* ≥ 0.79) for WGA samples. Panels (**C**) and (**D**) show the distribution of DNA methylation scores at the assessed CpG sites.

Next, we correlated the methylation levels at CpG sites assessed with RainDrop BS-seq and 450K arrays in the same tissue type. For the BM panel, the correlation was *R* ≥ 0.91 for unamplified and *R* ≥ 0.79 for WGA samples in BL (n = 81 CpG sites) and SCL (n = 204 CpG sites), as shown in Figure 4A and B. These trends were again confirmed using the SC panel (Fig. S5A–C). We found that, particularly for unamplified samples, the correlation was high across the entire quantitative range (Figs. S6A, 6B, and S7). DNA methylation scores showed a wide distribution across the assessed sites (Fig. 4C and D). CpGs in SCL samples were generally more methylated compared with BL samples.

For the BM panel, the CpG call rate was lower for the 10 and 50 ng WGA samples (16.7–33.5%) compared with all other samples tested (46.5–53.0%). For these WGA samples in SCL but not BL, we observed a considerable deviation of median methylation levels compared with all other samples (Fig. 4D). Nonetheless, the methylation levels assessed at these CpG sites in BL and SCL samples correlated reasonably well with 450K arrays (median *R* = 0.85). Thus, despite the reduced call rate for the 10 and 50 ng WGA samples, we note that methylation levels can still be estimated. Finally, a graphical representation of the methylation profiles obtained using RainDrop BS-seq is shown in Figure S8, using the example of the *LAMA2* and *KCNQ1* target loci.

### Conclusion

In this article, we assessed the recently introduced RainDrop BS-seq as a method for large-scale, targeted bisulfite sequencing. We demonstrated reliable microdroplet-based PCR amplification of up to 500 target loci in human whole blood, an osteosarcoma cell line and an FFPE sample. We showed that methylation profiles generated using a starting DNA amount of only 100 ng correlated well with 450K arrays. Further, we found that tiling of the genomic regions of choice using several overlapping PCR amplicons may improve target coverage. In our assessment, we did not find the application of MDA-based WGA to be beneficial in conjunction with the RainDrop BS-seq workflow concerning read coverage and CpG call rate, but presented good correlation of DNA methylation levels with those obtained on 450K arrays. In conclusion, we suggest that RainDrop BS-seq may be applicable to large-scale EWAS validation and replication experiments, even when only nanogram quantities of DNA are available. As the sample throughput for EWAS increases (akin to those for genome-wide association studies; GWAS), novel techniques such as RainDrop BS-seq are urgently needed to facilitate the experimental validation of the discovered association loci. Further studies (including one currently underway as part of the BLUEPRINT Consortium^22^) will address how the method presented here compares to other large-scale, targeted bisulfite sequencing methods.

## Materials and Methods

### Sample preparation

Genomic DNA was isolated from whole blood taken from a group of individuals using a GeneCatcher gDNA Blood Kit (Invitrogen) following the manufacturer’s protocol. Osteosarcoma 143B cells were cultured and genomic DNA prepared as previously described.^18^ The preparation of genomic DNA from a FFPE tissue sample of a neuroendocrine tumor omental metastasis (REC approval: 09/H0722/27) was described by Thirlwell et al.^23^ The DNA concentration was assessed using a Qubit dsDNA BR Assay Kit (Invitrogen).

### Bisulfite conversion and WGA

Genomic DNA was bisulfite-treated using an EpiTect Bisulfite Kit (QIAGEN). We followed the standard protocol according to the manufacturer’s instructions for BL and SCL samples, but used the optimized protocol for FFPE samples. For samples with a starting DNA material of less than 250 ng, we added Carrier RNA to BL Buffer. Purified bisulfite-converted DNA was eluted in 13 μl of EB Buffer. Selected samples were subjected to WGA using an EpiTect Whole Bisulfitome Kit (QIAGEN) following the manufacturer’s protocol. The entire sample of the bisulfite conversion reaction (~10 μl) was used for WGA.

### Microdroplet PCR

For microdroplet PCR, 7.20 μl of bisulfite-treated (and optionally, whole-genome amplified) DNA were added to 4.70 μl of 10× High-Fidelity Buffer (Invitrogen), 1.80 μl of 50 mM MgSO_4_ (Invitrogen), 1.62 μl of 10 mM dNTP solution mix (NEB), 3.60 μl of 4 M betaine solution (Sigma-Aldrich), 3.60 μl of droplet stabilizer (RainDance Technologies), 1.80 μl of 100% dimethyl sulfoxide (Sigma-Aldrich) and 0.72 μl of 5 U/μl Platinum Taq Polymerase High-Fidelity (Invitrogen), to a total volume of 25 μl. The sample plate was sealed using an ALPS 50V microplate heat sealer (Thermo Scientific).

The bisulfite-treated genomic DNA template mix was then applied to a fully automated ThunderStorm system (RainDance Technologies) following the manufacturer’s instructions. In brief, primer panel droplets (MethylSeq Solution, RainDance Technologies) were dispensed to a microfluidic chip. The DNA template mix was converted into droplets within the microfluidic chip. The primer pair droplets and template droplets were then paired together in a 1:1 ratio. Paired droplets passed through an electric field inducing the discrete droplets to coalesce into a single PCR droplet (26 pl). Approximately 1 million PCR droplets are usually collected per sample.

PCR droplets were processed in a PTC-225 thermocycler (MJ Research) as follows: 94 °C for 2 min; 55 cycles of 94 °C for 30 s, 54 °C for 45 s, 68 °C for 80 s; followed by 68 °C for 10 min; 4 °C until further processing. The ramp rate was set to 1 °C per second. Following PCR amplification, 70 μl of droplet destabilizer (RainDance Technologies) were added to each sample to break the PCR droplet emulsion and release the amplicons contained within the droplets. The solution was mixed well and incubated for 15 min at RT. Samples were purified using Agencourt AMPure XP magnetic beads (Beckman Coulter) following the manufacturer’s protocol. For each sample, 234 μl of beads were used. Samples were eluted from magnetic beads in 40 μl of EB Buffer. The integrity and concentration (fragment range: 120–250 bp) of purified amplicon DNA were assessed using a High Sensitivity DNA Kit (Agilent Technologies) on a 2100 Bioanalyzer (Agilent Technologies).

### Universal PCR

To prepare the samples for high-throughput DNA sequencing, Illumina adapter sequences and unique barcodes were introduced through an additional PCR step. Here, 15 ng of purified amplicon DNA were added to 3.25 μl of 10x High-Fidelity Buffer, 0.88 μl of 50 mM MgSO_4_, 0.88 μl of 10 mM dNTP solution mix, 2.50 μl of 4 M betaine solution, 1.25 μl of 100% dimethyl sulfoxide, 0.50 μl of 5 U/μl Platinum Taq Polymerase High-Fidelity and 2.5 μl of 5 μM PCR primers, to a total volume of 25 μl. All primer sequences are provided in Table S3A.

Samples were amplified as follows: 94 °C for 2 min; 10 cycles of 94 °C for 30 s, 56 °C for 45 s, 68 °C for 60 s; followed by 68 °C for 10 min; 4 °C until further processing. DNA was purified using a MinElute PCR Purification Kit (QIAGEN) according to the manufacturer’s protocol. Purified DNA was eluted in 10 μl of EB Buffer. Samples were quantified (fragment range: 100–400 bp and 220–450 bp for the SC and BM panel, respectively) using a DNA 1000 Kit on a 2100 Bioanalyzer. Of each sample, 50 ng were pooled. The resulting sequencing library was quantified using a Qubit dsDNA BR Assay Kit.

### High-throughput DNA sequencing

The pooled sequencing library (12 pM) and custom sequencing primers (0.5 μM) were applied to a MiSeq 300-cycle PE consumable cartridge (Illumina) according to the manufacturer’s protocol. The DNA sequences of the custom sequencing primers are provided in Table S3B. Sequencing was performed on a MiSeq DNA sequencer (Illumina) using 75-bp paired-end reads.

### Data and statistical analyses

Sequencing adapters were trimmed from the raw sequencing reads using the fastq-mcf tool of ea-utils v1.1.2-537 (parameter -k 0). Trimmed sequencing data were mapped to an in silico bisulfite-converted human reference genome (GRCh37) using Bismark v0.7.12 (parameters: -non_directional, -bowtie2).^24^ Methylation information was extracted using the methylation_extractor tool of Bismark v0.7.12 (parameters: -p, -comprehensive, -merge_non_CpG). Targeted DNA sequencing analyses were performed using the R package TEQC v3.2.0.^25^ All statistical analyses were performed in R, an environment for statistical computing.

### Methylation profiling on 450K arrays

Genomic DNA was prepared from whole blood and osteosarcoma 143B cells as described above. Of each sample, 500 ng of gDNA were bisulfite-converted using an EZ DNA Methylation Kit (Zymo Research) following the manufacturer’s instructions, but using optimized incubation conditions (i.e., 16 cycles of 95 °C for 30 s, 50 °C for 60 min; followed by 4 °C until further processing). Purified bisulfite-treated DNA was eluted in 15 μl of M-Elution Buffer. Array processing was performed following the Infinium HD Methylation Assay Guide (Illumina). Data analyses were performed using the R package ChAMP v1.0.6.^26^ In brief, IDAT files were transformed into intensity (beta) values. We filtered probes with a median detection *P* value ≥ 0.01 in at least one sample. Beta values were normalized using the BMIQ (Beta Mixture Quantile dilation) method.^27^ By applying this method, the beta values of type II design probes were adjusted into a statistical distribution characteristic of type I probes.

## Supplementary Material

Additional material

## Abbreviations:

BL: whole blood
EWAS: epigenome-wide association study
FFPE: formalin-fixed paraffin-embedded
MDA: multiple displacement amplification
SCL: osteosarcoma cell line
WGA: whole-genome amplification

## Notes

10.4161/epi.28041
