# Supplementary material for: Assessment of RainDrop BS-seq as a method for large-scale, targeted bisulfite sequencing
Source: Epigenetics. 2014 Feb 11;9(5):678–84. doi: 10.4161/epi.28041 (PMC4063826; doi:10.4161/epi.28041)
Supplement: Additional material [file epi-9-678-s01.pdf]

Landes Bioscience
www.landesbioscience.com

Supplemental Material to:

Dirk S Paul, Paul Guilhamon, Anna Karpathakis,
Lee M Butcher, Christina Thirlwell, Andrew Feber,
and Stephan Beck

Assessment of RainDrop BS-seq as a method for large-scale, targeted bisulfite sequencing

Epigenetics 2013; 9(5)
http://dx.doi.org/10.4161/epi.28041

http://www.landesbioscience.com/journals/epigenetics/
article/28041/

Assessment of RainDrop BS-seq as a method for large-scale, targeted bisulfite sequencing

SUPPLEMENTARY INFORMATION

Dirk S. Paul, Paul Guilhamon, Anna Karpathakis, Lee M. Butcher, Christina Thirlwell, Andrew Feber and Stephan Beck

UCL Cancer Institute; University College London; London, United Kingdom

Correspondence:

Dirk S. Paul (d.paul@ucl.ac.uk) and Stephan Beck (s.beck@ucl.ac.uk)

UCL Cancer Institute, 72 Huntley Street, London, WC1E 6DD, United Kingdom

Table of Contents

Figure S1. Digital gel electrophoresis of DNA amplicons following microdroplet PCR and purification....3

Figure S2. Digital gel electrophoresis of DNA amplicons following universal PCR and purification....4

Figure S3. Fraction of target bases with a coverage ranging from 1 to 1000 across target loci....5

Figure S4. Assessment of the quantitative resolution of the 450K array platform in comparison to RainDrop BS-seq....6

Figure S5. Correlation of DNA methylation levels and comparison to 450K arrays....7

Figure S6. Scatterplot of DNA methylation levels and comparison to 450K arrays....8

Figure S7. Distribution of DNA methylation levels over the entire dynamic range of the 450K array....9

Figure S8. Methylation profiling of the *LAMA2* and *KCNQ1* target loci....10

Table S1. Selected genomic loci assessed using RainDrop BS-seq....11

Table S2. Summary sequencing and methylation statistics for the primer panels....21

Table S3. Primer sequences for the universal PCR and Illumina sequencing reaction....24

Supplementary Figures

Figure S1. Digital gel electrophoresis of DNA amplicons following microdroplet PCR and purification. The PCR amplicons were analyzed using a High Sensitivity DNA Kit [Agilent Technologies]. Gels are shown for **(A)** BL, **(B)** SCL and **(C)** FFPE samples using the SC primer panel, and **(D)** BL and **(E)** SCL samples using the BM panel. The expected band of the amplified fragments was between 120 and 250 bp. The band indicating primer dimers was below 100 bp. For WGA samples, we observed bands corresponding to unspecific, high-molecular fragments.

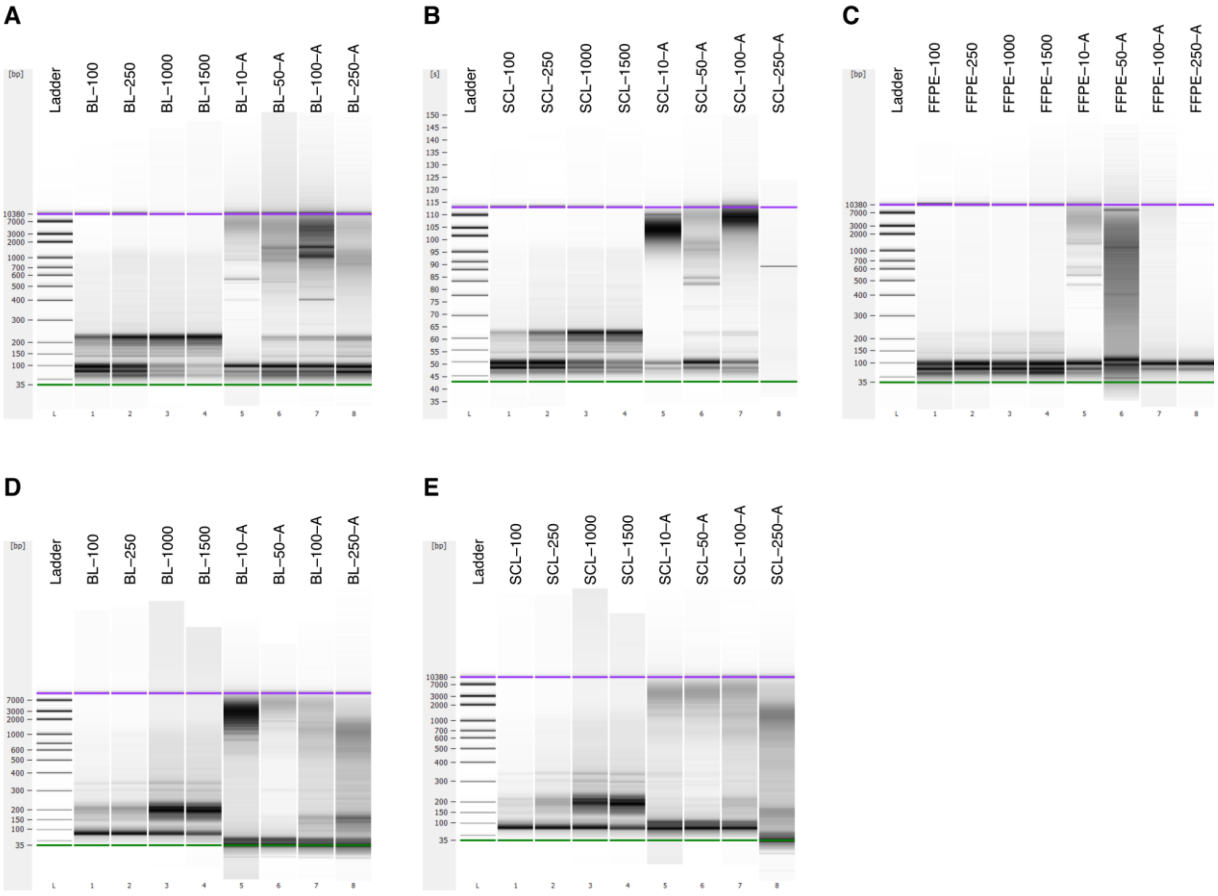

Figure S2. Digital gel electrophoresis of DNA amplicons following universal PCR and purification. The PCR amplicons were analyzed using a DNA 1000 Kit [Agilent Technologies]. Gels are shown for **(A)** BL, **(B)** SCL and **(C)** FFPE samples using the SC primer panel, and **(D)** BL and **(E)** SCL samples using the BM panel. The expected band of the amplified fragments after integration of Illumina adapters was shifted by +130 bp (Fig. S1 and Table S3A). Primer dimers (<100 bp), which could affect the subsequent DNA sequencing reaction, were not observed.

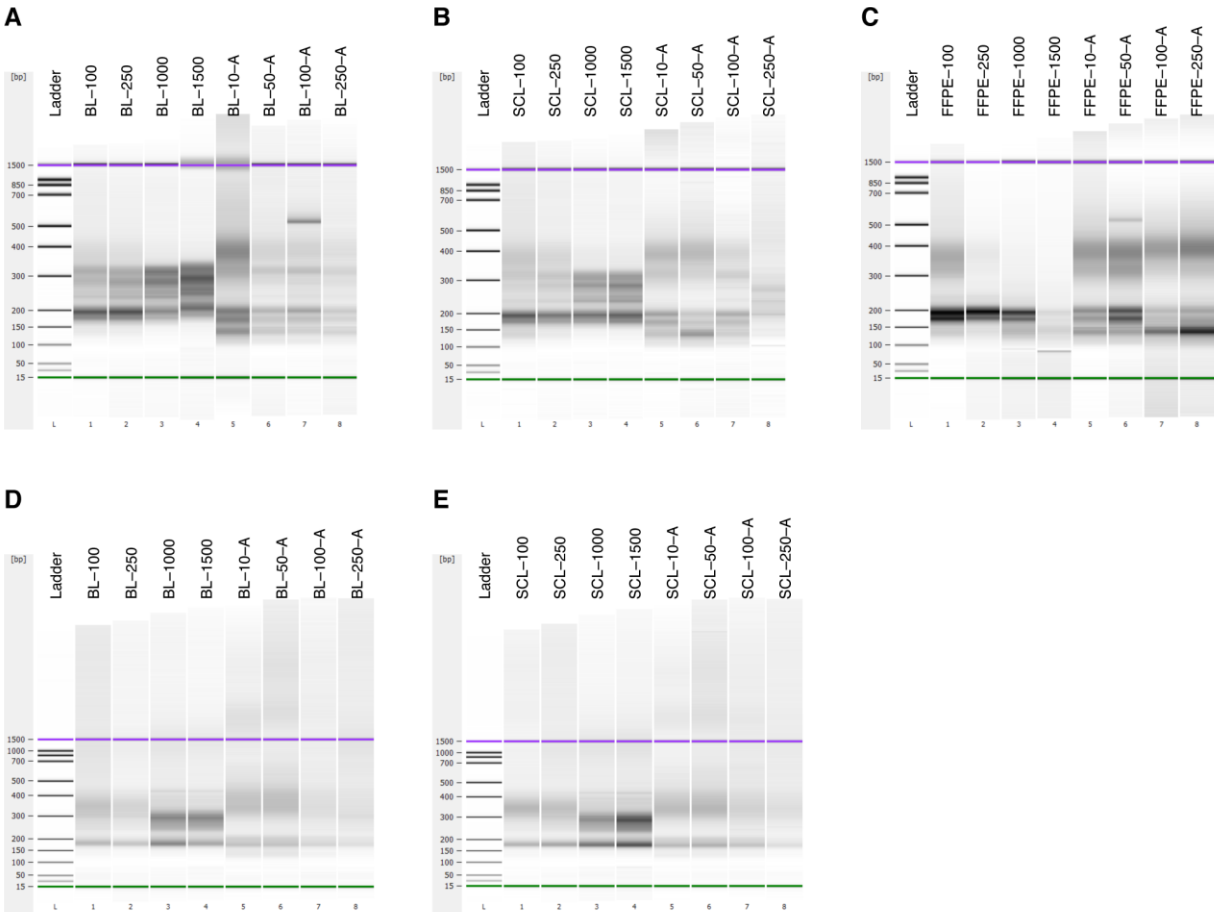

Figure S3. Fraction of target bases with a coverage ranging from 1 to 1000 across target loci. The plots show the fraction of target bases with a coverage ranging from 1 to 1000 at 27 target loci across **(A)** BL, **(B)** SCL and **(C)** FFPE samples, assessed using the SC panel. A coverage of five sequencing reads is indicated with a dotted line. For BL and SCL samples, the coverage profiles for unamplified samples as well as the 100 and 250 ng WGA samples, were relatively similar across different starting DNA amounts.

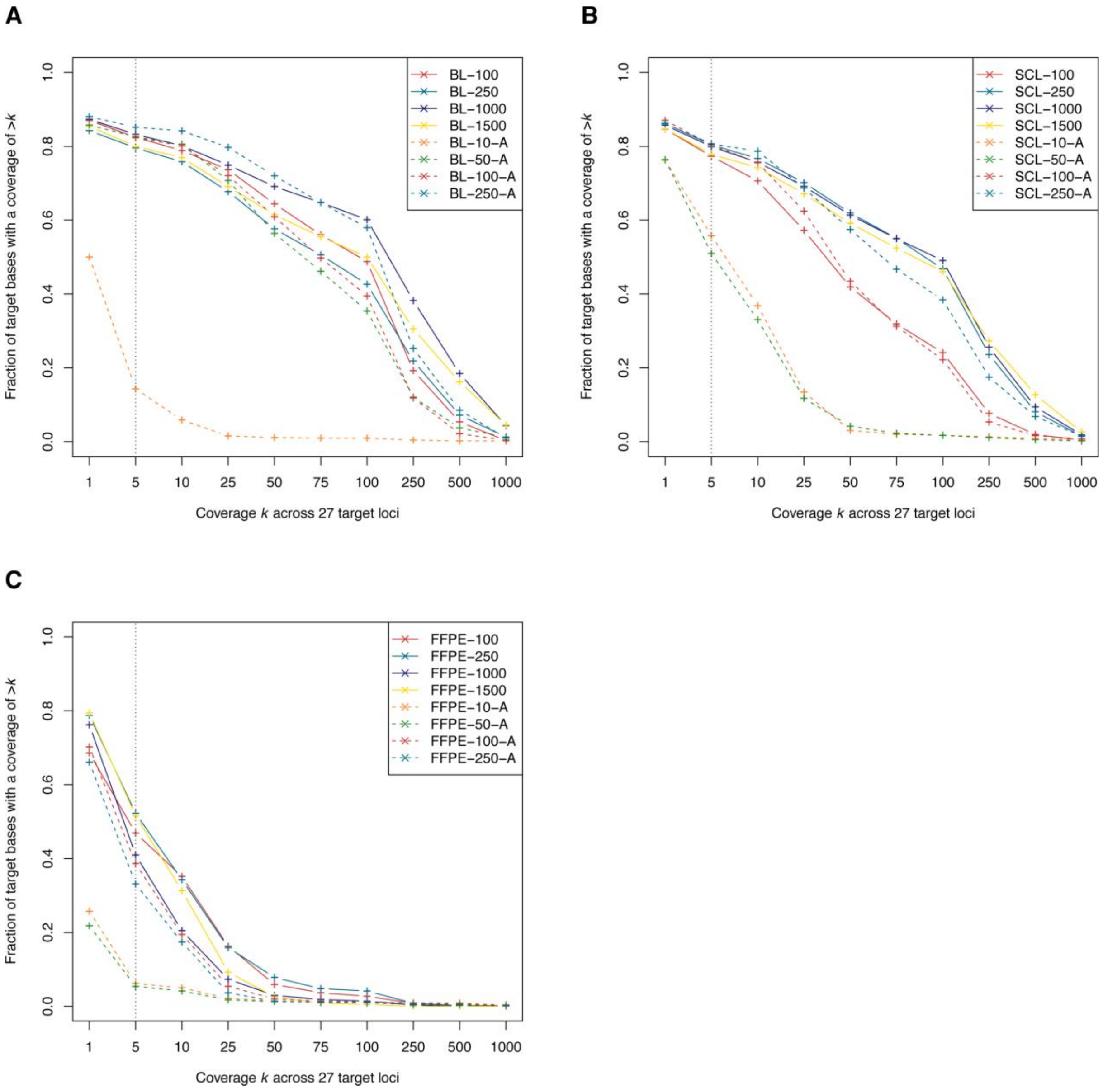

Figure S4. Assessment of the quantitative resolution of the 450K array platform in comparison to RainDrop BS-seq. We performed two technical replicates each of *in vitro* methylated and unmethylated whole blood genomic DNA. The *in vitro* methylated DNA was created using SssI methyltransferase. The *in vitro* unmethylated DNA was created by performing two sequential rounds of whole-genome amplification using REPLI-g. Array processing and analysis were performed as described in the Methods section. The figure shows the distribution of beta values for these experiments. Non-CpG probes (n=2800) are indicated with an asterisk. For *in vitro* methylated CpG sites we found that 95% of 450K probes had beta values above 0.82, while *in vitro* unmethylated CpGs had beta values below 0.31. On the basis of these data, the quantitative resolution of RainDrop BS-seq is comparable to 450K arrays to detect 'methylated' sites (i.e. beta≥0.82 vs ≥4/5 [≥80%] methylated reads), and actually exceeds the 450K array's ability to resolve 'unmethylated' sites (i.e. beta≤0.31 vs ≤1/5 [≤20%] methylated reads). Therefore, we suggest a quantitate resolution of 20% to be appropriate for the experiments performed in this study.

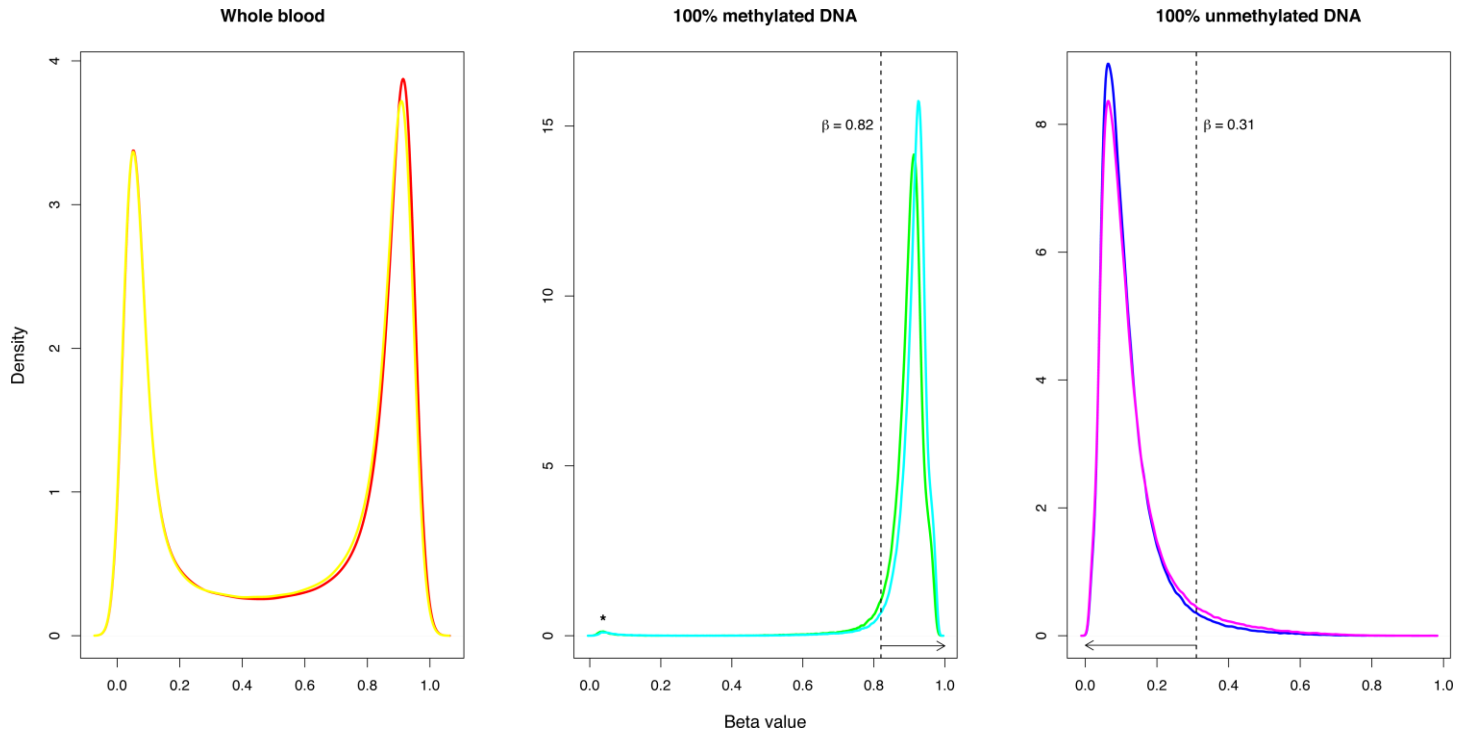

Figure S5. Correlation of DNA methylation levels and comparison to 450K arrays. The heatmaps show matrices of Pearson's correlation coefficients across **(A)** BL (n=240 CpG sites), **(B)** SCL (n=774 CpG sites) and **(C)** FFPE samples (n=13 CpG sites), assessed using the SC panel. We confirmed the strong correlation of DNA methylation levels found for unamplified samples using the BM panel (Fig. 4A,B), with a median R=0.97. Panels **(D)**, **(E)** and **(F)** show the distribution of DNA methylation scores at the assessed CpG sites.

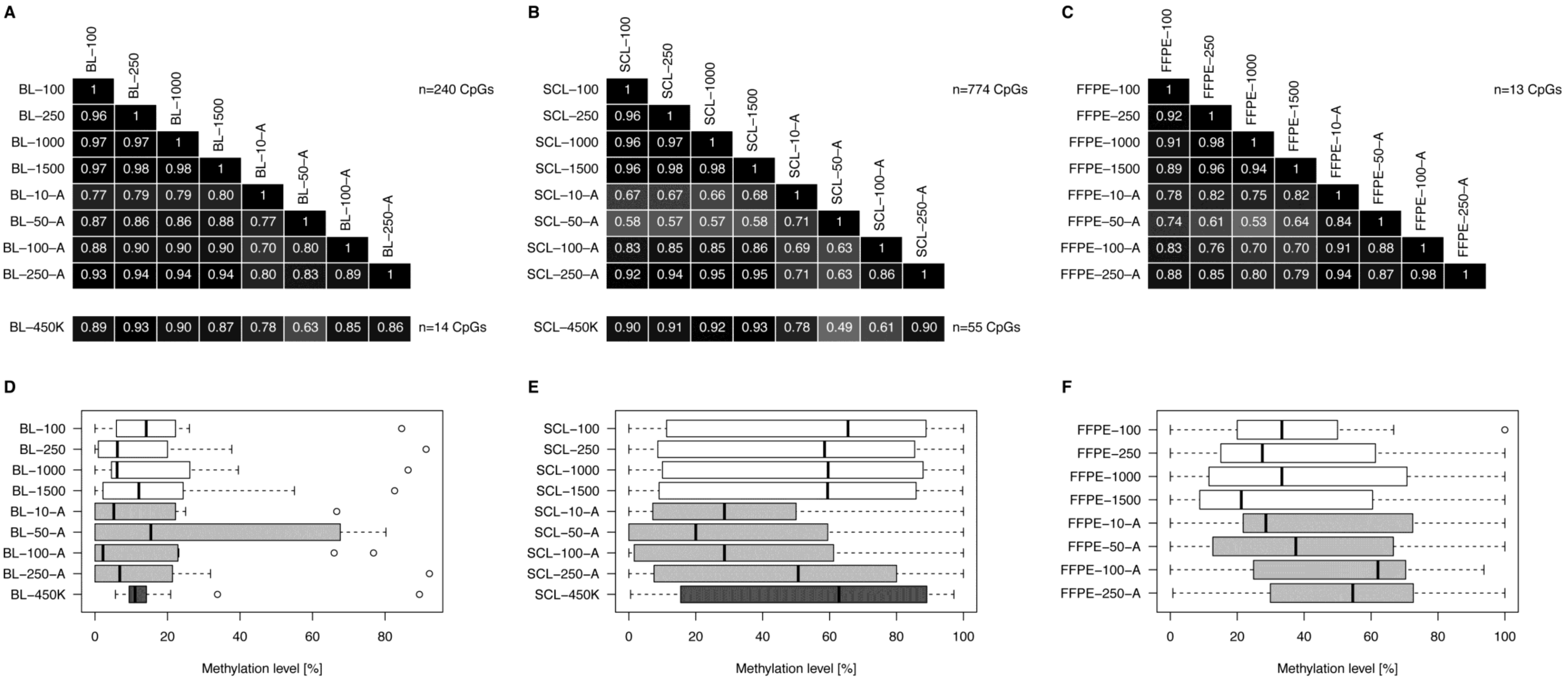

Figure S6. Scatterplot of DNA methylation levels and comparison to 450K arrays. (A) The scatterplot matrix shows the correlation of DNA methylation levels across BL samples (n=497 CpG sites), assessed using the BM panel. **(B)** In addition, we correlated a subset of these CpG sites that are represented on the 450K array (n=81). The assessed CpG sites correspond to those shown in Figure 4A.

A

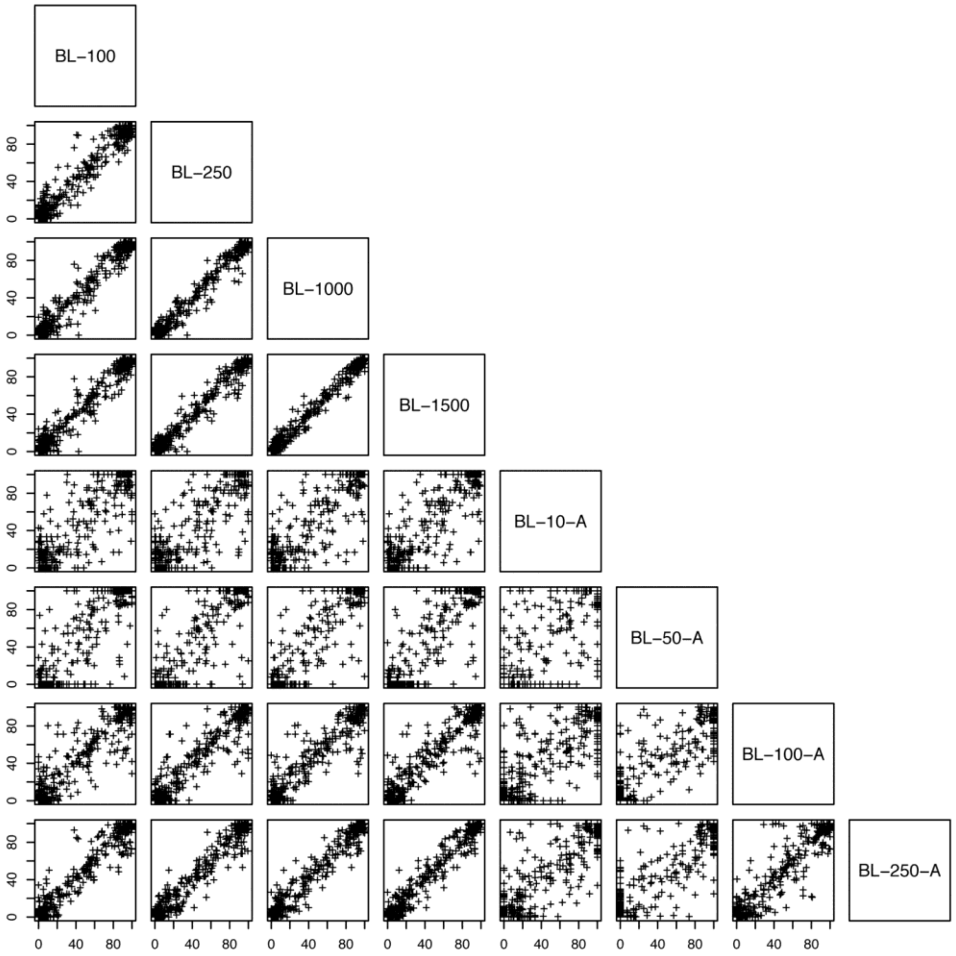

B

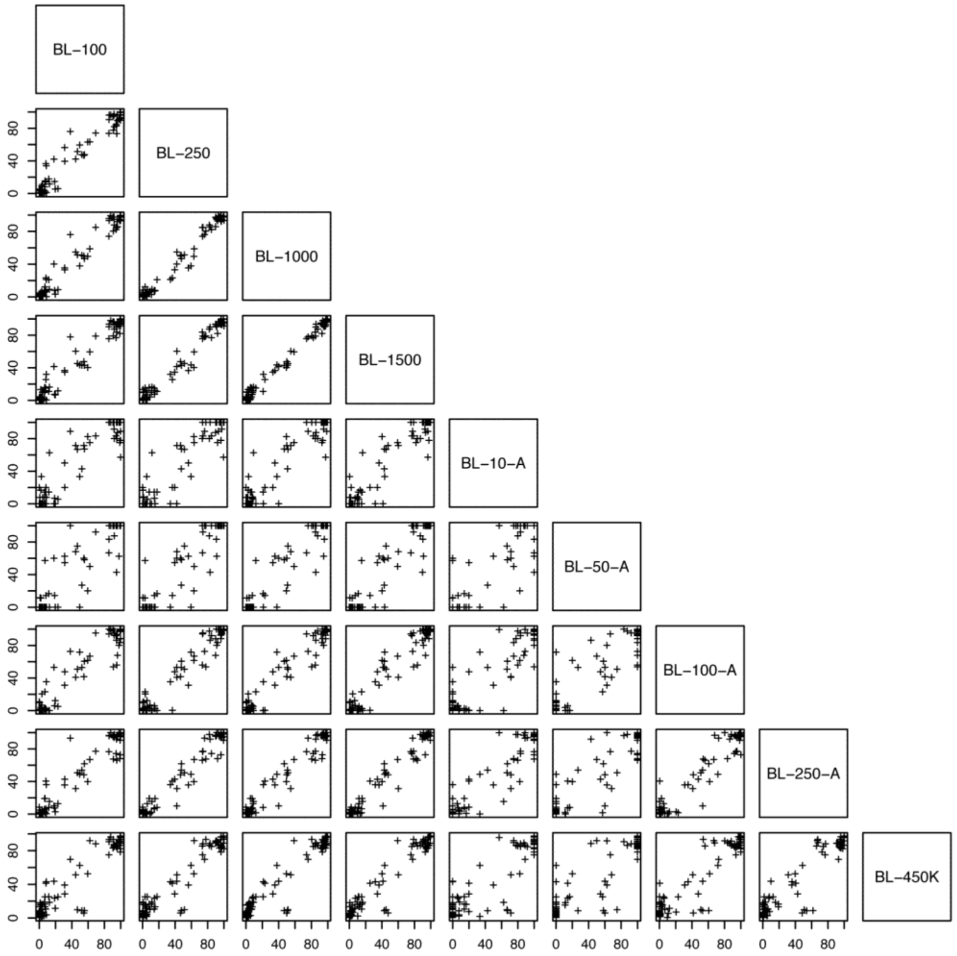

Figure S7. Distribution of DNA methylation levels over the entire dynamic range of the 450K array. The boxplots show the observed DNA methylation levels for CpG sites that, according to the 450K array, fall into low (0–20%), medium (20–80%) and high (80–100%) methylation bins (indicated with light blue shading). As in Figure S6B, we assessed CpG sites (n=81) in BL samples that are represented on the 450K array.

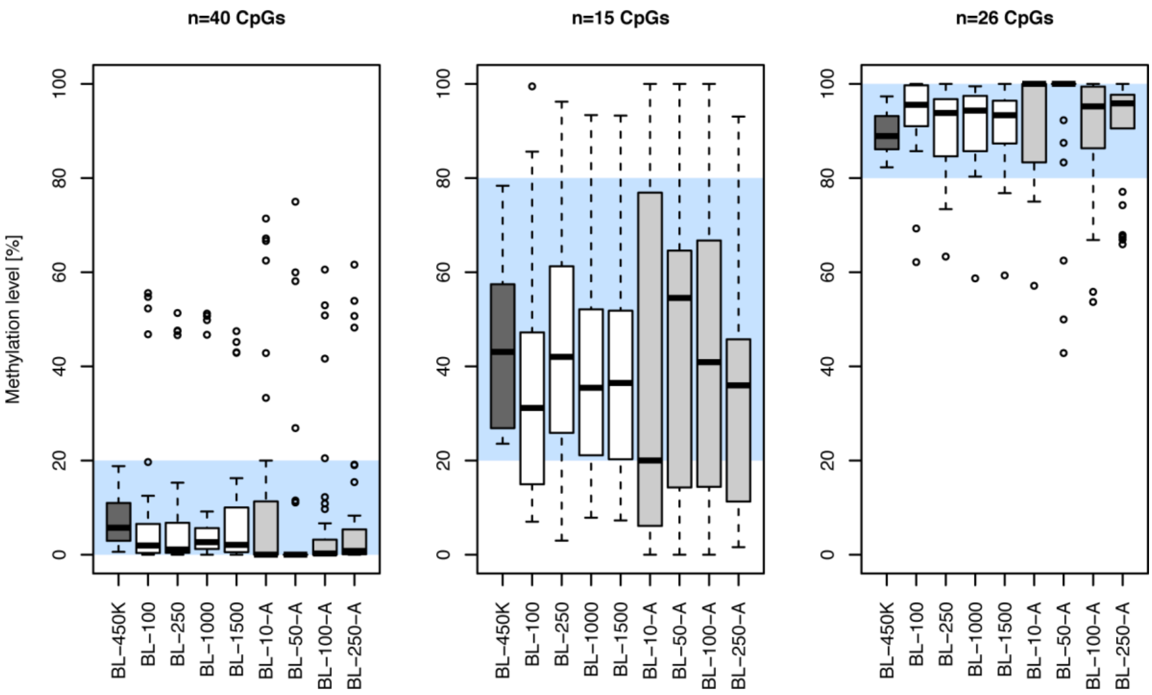

Figure S8. Methylation profiling of the (A) *LAMA2* and (B) *KCNQ1* target loci. The figure shows the *LAMA2* (BM primer panel; Locus ID: 161; Amplicon ID: 175) and *KCNQ1* (BM primer panel; Locus ID: 243; Amplicon ID: 263) gene loci in the UCSC Genome Browser. We annotated the genomic loci with the RainDrop BS-seq target region coordinates in gray, and the methylation levels (color range: yellow–blue) assessed in independent experiments using a range of starting DNA quantities (10–1500 ng) and application of MDA-based WGA of bisulfite-converted DNA (indicated with '-A'). We also added the methylation data generated on the 450K array platform.

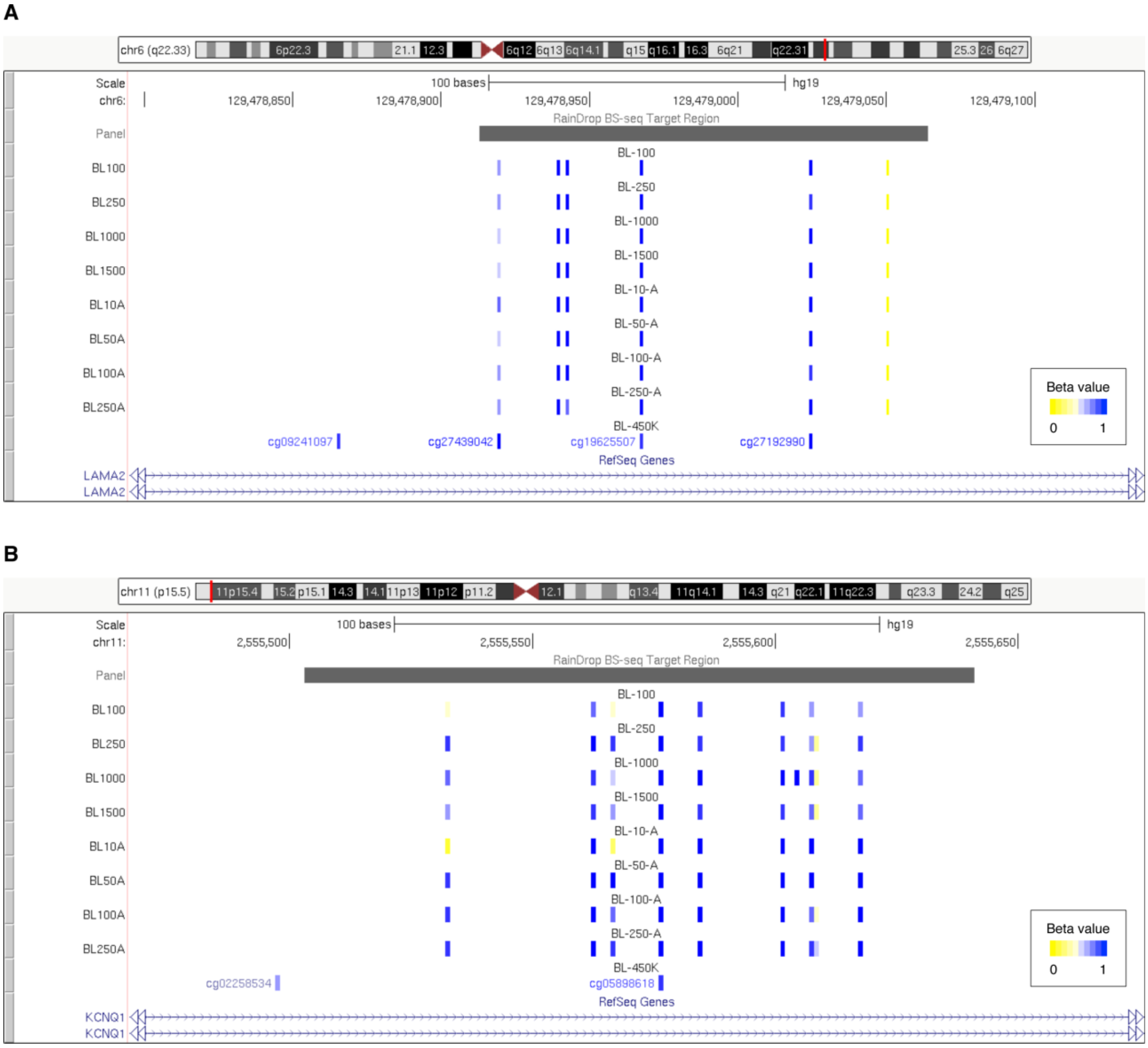

Supplementary Tables

Table S1. Selected genomic loci assessed using RainDrop BS-seq. The table provides genomic coordinates (hg19) of the target loci and PCR amplicons. In addition, CpG sites that are present on the 450K array and located within the target loci are indicated. **(A)** The SC primer panel targeted 27 genomic regions (500 PCR amplicons) containing a total of 212 CpG sites that are represented on the 450K array. **(B)** The BM panel targeted 462 candidate epigenetic biomarker loci (501 PCR amplicons) containing 778 of the 450K CpG sites.

(A) SC Panel

Locus	Amplicon				450K CpG Site	
ID	ID	Chr	Start	End	ID	Position
1	1	1	3,569,197	3,569,352	NA	NA
1	2	1	3,569,197	3,569,380	NA	NA
1	3	1	3,569,335	3,569,530	cg16741710	3,569,386
1	4	1	3,569,364	3,569,562	cg16741710	3,569,386
1	5	1	3,569,504	3,569,698	cg05924583	3,569,624
1	6	1	3,569,597	3,569,757	cg05924583	3,569,624
1	7	1	3,569,703	3,569,809	NA	NA
1	8	1	3,569,714	3,569,870	NA	NA
1	9	1	3,569,776	3,569,941	cg11504517	3,569,899
1	10	1	3,569,873	3,570,003	cg11504517	3,569,899
1	11	1	3,569,927	3,570,115	NA	NA
1	12	1	3,570,037	3,570,226	NA	NA
1	13	1	3,570,082	3,570,279	NA	NA
1	14	1	3,570,183	3,570,343	NA	NA
1	15	1	3,570,262	3,570,460	NA	NA
2	16	2	74,271,947	74,272,097	NA	NA
2	17	2	74,272,021	74,272,196	cg00694660	74,272,104
2	18	2	74,272,076	74,272,207	cg00694660	74,272,104
2	19	2	74,272,223	74,272,373	NA	NA
2	20	2	74,272,293	74,272,456	NA	NA
2	21	2	74,272,378	74,272,525	NA	NA
2	22	2	74,272,466	74,272,665	NA	NA
2	23	2	74,272,556	74,272,748	NA	NA
2	24	2	74,272,627	74,272,820	cg01355757	74,272,773
2	25	2	74,272,729	74,272,870	cg01355757	74,272,773
2	26	2	74,272,818	74,272,962	cg01244346	74,272,962
2	27	2	74,272,882	74,273,079	cg01244346	74,272,962
2	28	2	74,272,989	74,273,151	NA	NA
2	29	2	74,273,089	74,273,270	NA	NA
2	30	2	74,273,116	74,273,311	NA	NA
2	31	2	74,273,251	74,273,450	NA	NA
2	32	2	74,273,390	74,273,562	NA	NA
2	33	2	74,273,458	74,273,609	NA	NA
3	34	2	106,681,135	106,681,319	cg22948077	106,681,280
3	35	2	106,681,191	106,681,373	cg22948077	106,681,280
3	36	2	106,681,292	106,681,463	NA	NA
3	37	2	106,681,350	106,681,533	NA	NA
3	38	2	106,681,468	106,681,667	NA	NA
3	39	2	106,681,612	106,681,718	NA	NA
3	40	2	106,681,670	106,681,869	cg10735632	106,681,831
3	41	2	106,681,804	106,681,975	cg10735632	106,681,831
3	41	2	106,681,804	106,681,975	cg14535980	106,681,937
3	41	2	106,681,804	106,681,975	cg21838979	106,681,945
3	42	2	106,681,861	106,682,026	cg14535980	106,681,937
3	42	2	106,681,861	106,682,026	cg21838979	106,681,945
3	42	2	106,681,861	106,682,026	cg06499647	106,681,983
3	42	2	106,681,861	106,682,026	cg07510423	106,681,998
3	43	2	106,681,972	106,682,072	cg06499647	106,681,983
3	43	2	106,681,972	106,682,072	cg07510423	106,681,998
3	44	2	106,682,239	106,682,309	NA	NA
3	45	2	106,682,240	106,682,439	cg09661951	106,682,331
3	46	2	106,682,276	106,682,450	cg09661951	106,682,331
3	47	2	106,682,405	106,682,536	NA	NA
3	48	2	106,682,495	106,682,669	cg10885338	106,682,640
3	49	2	106,682,577	106,682,769	cg10885338	106,682,640
3	50	2	106,682,646	106,682,806	NA	NA
3	51	2	106,682,765	106,682,909	NA	NA
4	52	2	121,669,794	121,669,993	NA	NA
4	53	2	121,669,892	121,670,063	NA	NA
4	54	2	121,670,000	121,670,160	NA	NA

(B) BM Panel

Locus	Amplicon				450K CpG Site	
ID	ID	Chr	Start	End	ID	Position
1	1	1	949,741	949,920	cg04788999	949,850
1	1	1	949,741	949,920	cg16526047	949,893
2	2	1	1,158,573	1,158,728	cg03467014	1,158,648
3	3	1	2,266,291	2,266,447	cg16600733	2,266,362
3	3	1	2,266,291	2,266,447	cg02687883	2,266,431
4	4	1	3,459,820	3,460,014	cg06511312	3,459,949
5	5	1	3,567,586	3,567,765	cg07382920	3,567,646
5	5	1	3,567,586	3,567,765	cg10038618	3,567,719
5	5	1	3,567,586	3,567,765	cg24678611	3,567,732
5	5	1	3,567,586	3,567,765	cg25731359	3,567,738
6	6	1	10,579,082	10,579,235	cg10801633	10,579,190
7	7	1	12,123,442	12,123,552	NA	NA
7	8	1	12,123,527	12,123,696	cg05121790	12,123,554
8	9	1	14,343,060	14,343,254	cg13686807	14,343,114
9	10	1	21,978,575	21,978,724	cg18150885	21,978,612
9	10	1	21,978,575	21,978,724	cg08892236	21,978,698
10	11	1	21,986,195	21,986,331	cg05120085	21,986,286
11	12	1	22,615,848	22,616,017	cg02053477	22,615,900
12	13	1	25,257,516	25,257,669	cg15014975	25,257,547
12	13	1	25,257,516	25,257,669	cg24019564	25,257,566
12	13	1	25,257,516	25,257,669	cg10993442	25,257,587
12	13	1	25,257,516	25,257,669	cg24842859	25,257,599
12	13	1	25,257,516	25,257,669	cg20695936	25,257,624
12	13	1	25,257,516	25,257,669	cg13106389	25,257,626
12	13	1	25,257,516	25,257,669	cg18087266	25,257,629
13	14	1	47,898,845	47,898,969	cg05658236	47,898,911
14	15	1	55,752,451	55,752,568	cg03847541	55,752,514
15	16	1	65,991,125	65,991,285	cg08234308	65,991,176
16	17	1	87,795,021	87,795,189	cg03129717	87,795,122
17	18	1	91,182,737	91,182,936	cg18322569	91,182,777
17	18	1	91,182,737	91,182,936	cg17241310	91,182,856
18	19	1	110,052,257	110,052,519	cg05947402	110,052,352
18	19	1	110,052,257	110,052,519	cg21646186	110,052,458
18	19	1	110,052,257	110,052,519	cg12419308	110,052,464
18	19	1	110,052,257	110,052,519	cg06764670	110,052,490
19	20	1	111,175,024	111,175,158	cg05656442	111,175,082
20	21	1	115,124,440	115,124,544	cg20145149	115,124,491
21	22	1	121,261,272	121,261,451	cg07794500	121,261,369
21	22	1	121,261,272	121,261,451	cg05827631	121,261,404
22	23	1	144,930,878	144,931,062	cg02854922	144,930,964
23	24	1	146,547,949	146,548,127	cg12406683	146,548,032
24	25	1	147,736,119	147,736,302	cg21167167	147,736,190
25	26	1	155,271,700	155,271,834	cg15490880	155,271,762
26	27	1	161,442,628	161,442,808	cg08527195	161,442,697
26	27	1	161,442,628	161,442,808	cg09846895	161,442,738
27	28	1	171,811,400	171,811,594	cg20800956	171,811,477
27	28	1	171,811,400	171,811,594	cg16515523	171,811,529
28	29	1	177,002,175	177,002,347	cg22506490	177,002,228
29	30	1	202,317,732	202,317,906	cg05798608	202,317,770
29	30	1	202,317,732	202,317,906	cg13976253	202,317,780
30	31	1	209,822,863	209,823,015	cg07168232	209,822,928
31	32	1	210,425,198	210,425,298	cg14561217	210,425,254
31	33	1	210,425,255	210,425,409	NA	NA
32	34	1	224,400,579	224,400,742	cg01553584	224,400,672
33	35	1	227,057,956	227,058,113	cg20000641	227,058,046
33	35	1	227,057,956	227,058,113	cg00479101	227,058,070
34	36	1	230,289,965	230,290,156	cg22153407	230,290,089
35	37	1	231,981,776	231,981,974	cg09186051	231,981,906
36	38	1	232,940,913	232,941,059	cg02191044	232,940,990
36	38	1	232,940,913	232,941,059	cg02903907	232,941,055

4	55	2	121,670,110	121,670,296	cg03562360	121,670,282	37	39	1	246,582,016	246,582,206	cg10055505	246,582,035
4	56	2	121,670,153	121,670,350	cg03562360	121,670,282	37	39	1	246,582,016	246,582,206	cg07397891	246,582,071
4	56	2	121,670,153	121,670,350	cg06378990	121,670,320	37	39	1	246,582,016	246,582,206	cg16046505	246,582,116
4	57	2	121,670,273	121,670,461	cg03562360	121,670,282	38	40	2	3,642,528	3,642,702	cg19867917	3,642,629
4	57	2	121,670,273	121,670,461	cg06378990	121,670,320	38	40	2	3,642,528	3,642,702	cg09974661	3,642,634
4	58	2	121,670,331	121,670,508	NA	NA	39	41	2	7,843,846	7,844,006	cg26457075	7,843,941
4	59	2	121,670,464	121,670,663	cg01484075	121,670,634	40	42	2	8,597,248	8,597,424	cg09590377	8,597,389
4	60	2	121,670,560	121,670,697	cg01484075	121,670,634	40	43	2	8,597,366	8,597,546	cg09590377	8,597,389
4	61	2	121,670,640	121,670,812	NA	NA	41	44	2	9,518,197	9,518,378	cg19426625	9,518,324
4	62	2	121,670,684	121,670,880	NA	NA	42	45	2	9,614,498	9,614,606	cg01417553	9,614,528
4	63	2	121,670,791	121,670,976	NA	NA	42	45	2	9,614,498	9,614,606	cg17568255	9,614,530
4	64	2	121,670,920	121,671,111	NA	NA	42	45	2	9,614,498	9,614,606	cg20556744	9,614,537
4	65	2	121,670,947	121,671,138	NA	NA	42	45	2	9,614,498	9,614,606	cg05986044	9,614,578
4	66	2	121,671,092	121,671,257	NA	NA	43	46	2	27,274,440	27,274,590	cg07101579	27,274,487
4	67	2	121,671,202	121,671,388	NA	NA	43	46	2	27,274,440	27,274,590	cg20724004	27,274,548
4	68	2	121,671,320	121,671,517	NA	NA	43	46	2	27,274,440	27,274,590	cg18512092	27,274,554
5	69	2	209,119,417	209,119,595	NA	NA	44	47	2	27,305,486	27,305,648	cg16597102	27,305,552
5	70	2	209,119,548	209,119,735	NA	NA	45	48	2	31,455,343	31,455,494	cg13149833	31,455,443
5	71	2	209,119,616	209,119,795	cg24324379	209,119,750	46	49	2	37,814,978	37,815,162	cg23519155	37,815,128
5	72	2	209,119,721	209,119,878	cg24324379	209,119,750	47	50	2	38,204,758	38,204,912	cg17329835	38,204,832
5	72	2	209,119,721	209,119,878	cg16863783	209,119,814	48	51	2	42,275,692	42,275,791	cg12630082	42,275,740
5	73	2	209,119,838	209,119,992	cg09406631	209,119,940	49	52	2	66,562,381	66,562,544	cg14068057	66,562,437
5	73	2	209,119,838	209,119,992	cg07129696	209,119,949	50	53	2	73,518,659	73,518,828	cg26049726	73,518,702
5	73	2	209,119,838	209,119,992	cg10356455	209,119,992	51	54	2	75,792,113	75,792,262	cg02181594	75,792,215
5	74	2	209,119,942	209,120,012	cg07129696	209,119,949	51	55	2	75,792,189	75,792,359	cg02181594	75,792,215
5	74	2	209,119,942	209,120,012	cg10356455	209,119,992	52	56	2	80,459,230	80,459,355	cg17331757	80,459,300
5	74	2	209,119,942	209,120,012	cg25795625	209,120,001	53	57	2	101,500,472	101,500,628	cg12737833	101,500,543
5	75	2	209,119,949	209,120,082	cg10356455	209,119,992	54	58	2	103,089,994	103,090,158	cg02567476	103,090,077
5	75	2	209,119,949	209,120,082	cg25795625	209,120,001	55	59	2	105,489,829	105,489,978	cg00500333	105,489,930
5	76	2	209,120,004	209,120,157	cg16085695	209,120,094	56	60	2	129,076,290	129,076,416	cg17977173	129,076,335
5	77	2	209,120,097	209,120,166	NA	NA	56	60	2	129,076,290	129,076,416	cg13525428	129,076,361
5	78	2	209,120,308	209,120,397	NA	NA	56	60	2	129,076,290	129,076,416	cg25180075	129,076,367
5	79	2	209,120,309	209,120,426	NA	NA	56	60	2	129,076,290	129,076,416	cg17032602	129,076,392
5	80	2	209,120,346	209,120,541	NA	NA	56	60	2	129,076,290	129,076,416	cg23425970	129,076,394
5	81	2	209,120,454	209,120,602	NA	NA	57	61	2	131,553,877	131,554,064	cg01825818	131,554,018
5	82	2	209,120,546	209,120,745	NA	NA	58	62	2	149,215,467	149,215,617	cg06758980	149,215,535
5	83	2	209,120,657	209,120,854	NA	NA	59	63	2	155,314,703	155,314,819	cg11065425	155,314,765
5	84	2	209,120,766	209,120,935	NA	NA	60	64	2	162,209,866	162,210,015	cg08135437	162,209,962
5	85	2	209,120,873	209,120,996	NA	NA	61	65	2	164,593,197	164,593,392	cg20593611	164,593,221
5	86	2	209,120,921	209,121,067	NA	NA	61	65	2	164,593,197	164,593,392	cg04457196	164,593,264
5	87	2	209,120,986	209,121,094	NA	NA	62	66	2	201,390,205	201,390,369	cg24930416	201,390,305
5	88	2	209,121,048	209,121,157	NA	NA	63	67	2	203,777,779	203,777,957	cg15057621	203,777,863
6	89	2	220,378,123	220,378,308	NA	NA	64	68	2	220,114,225	220,114,340	cg13315450	220,114,289
6	90	2	220,378,269	220,378,459	NA	NA	65	69	2	232,393,132	232,393,308	cg01616956	232,393,196
6	91	2	220,378,397	220,378,572	NA	NA	65	69	2	232,393,132	232,393,308	cg22069247	232,393,256
6	92	2	220,378,522	220,378,648	NA	NA	66	70	2	240,174,578	240,174,711	cg06478504	240,174,613
6	93	2	220,378,578	220,378,775	cg11248254	220,378,659	66	70	2	240,174,578	240,174,711	cg17939889	240,174,650
6	94	2	220,378,729	220,378,889	NA	NA	66	70	2	240,174,578	240,174,711	cg14020052	240,174,659
6	95	2	220,378,803	220,378,930	NA	NA	67	71	2	241,235,309	241,235,468	cg04585787	241,235,366
6	96	2	220,378,879	220,379,065	cg10515731	220,378,996	68	72	2	241,396,188	241,396,315	cg15700022	241,396,251
6	96	2	220,378,879	220,379,065	cg18844301	220,379,000	69	73	2	242,794,819	242,795,002	cg07281781	242,794,854
6	96	2	220,378,879	220,379,065	cg09726982	220,379,016	69	73	2	242,794,819	242,795,002	cg10526431	242,794,928
6	96	2	220,378,879	220,379,065	cg17610929	220,379,044	69	73	2	242,794,819	242,795,002	cg09319815	242,794,941
6	96	2	220,378,879	220,379,065	cg24743778	220,379,058	69	73	2	242,794,819	242,795,002	cg06291111	242,794,981
6	97	2	220,378,969	220,379,167	cg10515731	220,378,996	70	74	3	5,470,829	5,470,957	cg15446845	5,470,909
6	97	2	220,378,969	220,379,167	cg18844301	220,379,000	71	75	3	25,482,829	25,483,013	cg15011775	25,482,937
6	97	2	220,378,969	220,379,167	cg09726982	220,379,016	71	75	3	25,482,829	25,483,013	cg06705767	25,483,002
6	97	2	220,378,969	220,379,167	cg17610929	220,379,044	72	76	3	30,933,682	30,933,878	cg04074353	30,933,751
6	97	2	220,378,969	220,379,167	cg24743778	220,379,058	73	77	3	33,318,580	33,318,779	cg19241168	33,318,720
6	97	2	220,378,969	220,379,167	cg19210770	220,379,091	74	78	3	45,838,037	45,838,171	cg02716516	45,838,074
6	97	2	220,378,969	220,379,167	cg18395327	220,379,109	74	78	3	45,838,037	45,838,171	cg20829379	45,838,083
6	98	2	220,379,048	220,379,231	cg24743778	220,379,058	74	78	3	45,838,037	45,838,171	cg25924911	45,838,094
6	98	2	220,379,048	220,379,231	cg19210770	220,379,091	75	79	3	49,211,666	49,211,849	cg24090938	49,211,749
6	98	2	220,379,048	220,379,231	cg18395327	220,379,109	76	80	3	51,990,160	51,990,344	cg22621867	51,990,301
6	98	2	220,379,048	220,379,231	cg12046414	220,379,199	76	81	3	51,990,272	51,990,396	cg22621867	51,990,301
6	99	2	220,379,172	220,379,351	cg12046414	220,379,199	77	82	3	62,356,861	62,357,044	cg20072171	62,356,962
6	99	2	220,379,172	220,379,351	cg21767952	220,379,260	78	83	3	72,897,587	72,897,751	cg00702577	72,897,627
6	100	2	220,379,265	220,379,423	NA	NA	78	83	3	72,897,587	72,897,751	cg24248486	72,897,631
6	101	2	220,379,375	220,379,527	NA	NA	78	83	3	72,897,587	72,897,751	cg14917700	72,897,678
6	102	2	220,379,392	220,379,556	NA	NA	79	84	3	75,701,927	75,702,053	cg12107367	75,701,993
6	103	2	220,379,508	220,379,667	cg03904220	220,379,635	80	85	3	98,482,698	98,482,841	cg04068578	98,482,769
6	104	2	220,379,577	220,379,755	cg03904220	220,379,635	81	86	3	101,431,406	101,431,569	cg21948827	101,431,499
6	104	2	220,379,577	220,379,755	cg14166053	220,379,755	82	87	3	109,033,814	109,033,967	cg16928393	109,033,890
6	105	2	220,379,684	220,379,861	cg14166053	220,379,755	83	88	3	119,217,055	119,217,214	cg19231082	119,217,133
6	105	2	220,379,684	220,379,861	cg20402664	220,379,833	83	88	3	119,217,055	119,217,214	cg02942644	119,217,190
7	106	4	106,066,221	106,066,408	NA	NA	84	89	3	128,513,965	128,514,126	cg15033153	128,514,015
7	107	4	106,066,336	106,066,531	NA	NA	85	90	3	133,611,557	133,611,716	cg00448875	133,611,626
7	108	4	106,066,388	106,066,544	NA	NA	86	91	3	139,253,778	139,253,936	cg18707191	139,253,838
7	109	4	106,066,493	106,066,622	NA	NA	87	92	3	142,837,864	142,838,060	cg19442495	142,837,995
7	110	4	106,066,520	106,066,691	NA	NA	87	92	3	142,837,864	142,838,060	cg19788429	142,838,003
7	111	4	106,066,586	106,066,731	NA	NA	88	93	3	159,886,848	159,887,041	cg05581878	159,886,981
7	112	4	106,066,646	106,066,793	NA	NA	89	94	3	161,577,613	161,577,746	NA	NA
7	113	4	106,066,727	106,066,921	NA	NA	90	95	3	169,351,372	169,351,484	cg04414509	169,351,419
7	114	4	106,066,765	106,066,933	NA	NA	91	96	3	170,136,711	170,136,815	cg02241055	170,136,766
7	115	4	106,066,877	106,067,020	NA	NA	92	97	3	186,256,281	186,256,392	cg15025362	186,256,331

7	116	4	106,066,916	106,067,046	NA	NA
7	117	4	106,067,001	106,067,123	cg08924430	106,067,052
7	118	4	106,067,014	106,067,197	cg08924430	106,067,052
7	119	4	106,067,144	106,067,302	NA	NA
7	120	4	106,067,232	106,067,398	cg06795971	106,067,365
7	120	4	106,067,232	106,067,398	cg09381178	106,067,367
7	121	4	106,067,279	106,067,431	cg06795971	106,067,365
7	121	4	106,067,279	106,067,431	cg09381178	106,067,367
7	122	4	106,067,376	106,067,524	NA	NA
7	123	4	106,067,458	106,067,657	cg01210909	106,067,566
7	123	4	106,067,458	106,067,657	cg14330655	106,067,570
7	124	4	106,067,568	106,067,753	cg14330655	106,067,570
7	124	4	106,067,568	106,067,753	cg13440296	106,067,738
7	125	4	106,067,634	106,067,748	cg13440296	106,067,738
8	126	5	179,243,488	179,243,685	NA	NA
8	127	5	179,243,632	179,243,768	NA	NA
8	128	5	179,243,716	179,243,908	NA	NA
8	129	5	179,243,846	179,243,956	cg11250941	179,243,916
8	130	5	179,243,882	179,244,041	cg11250941	179,243,916
8	131	5	179,243,979	179,244,175	NA	NA
8	132	5	179,244,021	179,244,217	NA	NA
8	133	5	179,244,157	179,244,295	NA	NA
8	134	5	179,244,193	179,244,360	cg06861426	179,244,317
8	135	5	179,244,271	179,244,436	cg06861426	179,244,317
8	136	5	179,244,379	179,244,564	cg24421668	179,244,452
8	137	5	179,244,420	179,244,619	cg24421668	179,244,452
8	138	5	179,244,534	179,244,721	NA	NA
8	139	5	179,244,598	179,244,771	NA	NA
8	140	5	179,244,704	179,244,880	NA	NA
8	141	5	179,244,853	179,244,936	NA	NA
8	142	5	179,244,909	179,245,088	NA	NA
8	143	5	179,245,041	179,245,237	NA	NA
9	144	6	46,702,685	46,702,789	NA	NA
9	145	6	46,702,726	46,702,889	cg21269330	46,702,790
9	146	6	46,702,772	46,702,960	cg21269330	46,702,790
9	147	6	46,702,900	46,703,058	cg18630040	46,702,983
9	148	6	46,703,038	46,703,156	cg26705953	46,703,123
9	149	6	46,703,145	46,703,275	cg04851935	46,703,193
9	149	6	46,703,145	46,703,275	cg27087057	46,703,222
9	149	6	46,703,145	46,703,275	cg08383338	46,703,225
9	149	6	46,703,145	46,703,275	cg23277376	46,703,232
9	149	6	46,703,145	46,703,275	cg18491375	46,703,235
9	149	6	46,703,145	46,703,275	cg26780998	46,703,251
9	150	6	46,703,198	46,703,380	cg27087057	46,703,222
9	150	6	46,703,198	46,703,380	cg08383338	46,703,225
9	150	6	46,703,198	46,703,380	cg23277376	46,703,232
9	150	6	46,703,198	46,703,380	cg18491375	46,703,235
9	150	6	46,703,198	46,703,380	cg26780998	46,703,251
9	150	6	46,703,198	46,703,380	cg04043742	46,703,315
9	151	6	46,703,260	46,703,412	cg04043742	46,703,315
9	152	6	46,703,353	46,703,476	cg17211404	46,703,422
9	152	6	46,703,353	46,703,476	cg05947443	46,703,440
9	152	6	46,703,353	46,703,476	cg03700944	46,703,468
9	153	6	46,703,410	46,703,602	cg17211404	46,703,422
9	153	6	46,703,410	46,703,602	cg05947443	46,703,440
9	153	6	46,703,410	46,703,602	cg03700944	46,703,468
9	153	6	46,703,410	46,703,602	cg19702271	46,703,495
9	153	6	46,703,410	46,703,602	cg16536824	46,703,504
9	153	6	46,703,410	46,703,602	cg07219955	46,703,538
9	153	6	46,703,410	46,703,602	cg11874627	46,703,554
9	154	6	46,703,447	46,703,618	cg03700944	46,703,468
9	154	6	46,703,447	46,703,618	cg19702271	46,703,495
9	154	6	46,703,447	46,703,618	cg16536824	46,703,504
9	154	6	46,703,447	46,703,618	cg07219955	46,703,538
9	154	6	46,703,447	46,703,618	cg11874627	46,703,554
9	155	6	46,703,567	46,703,717	cg12854458	46,703,670
9	156	6	46,703,637	46,703,836	cg12854458	46,703,670
9	157	6	46,703,685	46,703,884	NA	NA
9	158	6	46,703,798	46,703,944	NA	NA
9	159	6	46,703,877	46,704,074	NA	NA
9	160	6	46,704,016	46,704,200	cg26825569	46,704,077
9	161	6	46,704,105	46,704,251	NA	NA
9	162	6	46,704,161	46,704,279	NA	NA
9	163	6	46,704,219	46,704,386	NA	NA
10	164	6	166,581,556	166,581,738	NA	NA
10	165	6	166,581,671	166,581,831	NA	NA
10	166	6	166,581,716	166,581,858	NA	NA
10	167	6	166,581,810	166,581,969	cg23688510	166,581,929
10	168	6	166,581,834	166,581,980	cg23688510	166,581,929
10	169	6	166,581,925	166,582,038	cg23688510	166,581,929
10	170	6	166,581,946	166,582,090	NA	NA
10	171	6	166,582,014	166,582,176	cg02149708	166,582,159
10	172	6	166,582,099	166,582,274	cg02149708	166,582,159
10	172	6	166,582,099	166,582,274	cg05655837	166,582,188
10	172	6	166,582,099	166,582,274	cg17188046	166,582,197

93	98	4	106,652	106,845	cg10858327	106,702
93	98	4	106,652	106,845	cg10523696	106,750
94	99	4	1,229,399	1,229,525	cg22510098	1,229,471
95	100	4	3,480,198	3,480,374	cg23495837	3,480,318
96	101	4	3,748,167	3,748,612	cg08213608	3,748,254
96	101	4	3,748,167	3,748,612	cg22475974	3,748,341
96	101	4	3,748,167	3,748,612	cg03282085	3,748,347
96	101	4	3,748,167	3,748,612	cg05835309	3,748,554
97	102	4	6,695,297	6,695,437	cg27027375	6,695,319
97	102	4	6,695,297	6,695,437	cg14900031	6,695,377
97	102	4	6,695,297	6,695,437	cg14140379	6,695,380
98	103	4	7,526,573	7,526,699	cg17564203	7,526,639
98	103	4	7,526,573	7,526,699	cg14372037	7,526,671
98	104	4	7,526,639	7,526,776	cg14372037	7,526,671
99	105	4	20,255,267	20,255,411	cg08428452	20,255,343
100	106	4	22,517,023	22,517,221	cg13124370	22,517,059
100	106	4	22,517,023	22,517,221	cg08141192	22,517,094
100	106	4	22,517,023	22,517,221	cg26631477	22,517,195
101	107	4	55,521,318	55,521,431	cg08985766	55,521,379
102	108	4	79,642,447	79,642,612	cg17670237	79,642,491
103	109	4	96,471,024	96,471,208	cg12584684	96,471,105
103	109	4	96,471,024	96,471,208	cg15701178	96,471,143
104	110	4	102,160,467	102,160,627	cg19688772	102,160,529
105	111	4	108,641,415	108,641,537	cg08823359	108,641,437
105	111	4	108,641,415	108,641,537	cg15567057	108,641,439
106	112	4	114,758,333	114,758,481	cg08700197	114,758,389
107	113	4	118,975,389	118,975,545	cg12311175	118,975,504
108	114	4	122,795,688	122,795,815	cg09381441	122,795,753
109	115	4	128,553,820	128,554,011	cg25442239	128,553,912
109	115	4	128,553,820	128,554,011	cg13084458	128,553,917
109	115	4	128,553,820	128,554,011	cg11779273	128,553,930
110	116	4	128,983,720	128,983,896	cg19910937	128,983,807
111	117	4	129,209,533	129,209,682	cg13469311	129,209,602
111	117	4	129,209,533	129,209,682	cg24865292	129,209,608
112	118	4	139,485,767	139,485,881	cg17599582	139,485,823
113	119	4	147,557,808	147,557,940	cg01572513	147,557,859
114	120	4	154,710,319	154,710,457	cg03202804	154,710,353
114	120	4	154,710,319	154,710,457	cg11354906	154,710,371
114	120	4	154,710,319	154,710,457	cg25775322	154,710,373
114	120	4	154,710,319	154,710,457	cg14330641	154,710,399
114	120	4	154,710,319	154,710,457	cg05164933	154,710,418
114	120	4	154,710,319	154,710,457	cg23121156	154,710,421
114	120	4	154,710,319	154,710,457	cg23292160	154,710,425
114	120	4	154,710,319	154,710,457	cg10942078	154,710,429
114	121	4	154,710,431	154,710,628	cg14289246	154,710,475
114	121	4	154,710,431	154,710,628	cg22178613	154,710,499
114	121	4	154,710,431	154,710,628	cg05961809	154,710,523
114	121	4	154,710,431	154,710,628	cg23207990	154,710,535
114	121	4	154,710,431	154,710,628	cg25645268	154,710,598
115	122	4	159,740,724	159,740,868	cg21388639	159,740,817
116	123	4	169,542,972	169,543,161	cg21966319	169,543,126
116	124	4	169,543,089	169,543,188	cg21966319	169,543,126
117	125	4	174,450,682	174,450,821	cg10541864	174,450,722
118	126	4	186,306,006	186,306,164	cg15990090	186,306,065
119	127	4	188,149,336	188,149,495	cg12797684	188,149,410
120	128	5	51,336	51,489	cg08767686	51,426
121	129	5	1,123,684	1,123,864	cg03863908	1,123,738
121	129	5	1,123,684	1,123,864	cg15827843	1,123,765
121	129	5	1,123,684	1,123,864	cg00573504	1,123,809
122	130	5	14,294,742	14,294,872	cg04115026	14,294,830
122	131	5	14,294,823	14,294,935	cg04115026	14,294,830
123	132	5	14,326,068	14,326,212	cg12547959	14,326,153
124	133	5	16,856,979	16,857,177	cg01808849	16,857,119
125	134	5	34,655,963	34,656,124	cg03332113	34,656,078
126	135	5	35,919,420	35,919,555	cg13894535	35,919,491
127	136	5	37,371,519	37,371,681	cg22433285	37,371,555
127	136	5	37,371,519	37,371,681	cg15827031	37,371,628
128	137	5	52,773,032	52,773,191	cg18899355	52,773,135
129	138	5	54,281,176	54,281,354	cg14972155	54,281,198
129	138	5	54,281,176	54,281,354	cg06837426	54,281,271
129	138	5	54,281,176	54,281,354	cg20451680	54,281,336
130	139	5	73,623,151	73,623,323	cg17680767	73,623,208
131	140	5	89,000,602	89,000,757	cg00226580	89,000,691
132	141	5	95,409,024	95,409,183	cg21609975	95,409,135
133	142	5	111,869,493	111,869,615	cg03620123	111,869,581
133	143	5	111,869,551	111,869,673	cg03620123	111,869,581
134	144	5	118,691,004	118,691,118	cg17471836	118,691,007
134	144	5	118,691,004	118,691,118	cg07086380	118,691,033
134	144	5	118,691,004	118,691,118	cg23985447	118,691,066
135	145	5	135,364,261	135,364,406	cg18418457	135,364,328
135	145	5	135,364,261	135,364,406	cg09584591	135,364,349
136	146	5	137,683,308	137,683,408	cg11672479	137,683,379
136	147	5	137,683,361	137,683,490	cg11672479	137,683,379
137	148	5	138,608,461	138,608,599	cg25391820	138,608,519
138	149	5	140,686,132	140,686,272	cg07205661	140,686,191

10	172	6	166,582,099	166,582,274	cg14638883	166,582,201
10	172	6	166,582,099	166,582,274	cg19675288	166,582,206
10	173	6	166,582,225	166,582,406	cg06073449	166,582,310
10	173	6	166,582,225	166,582,406	cg06463958	166,582,393
10	174	6	166,582,385	166,582,574	cg06463958	166,582,393
10	174	6	166,582,385	166,582,574	cg08214847	166,582,464
10	175	6	166,582,434	166,582,625	cg08214847	166,582,464
10	175	6	166,582,434	166,582,625	cg13032463	166,582,585
10	176	6	166,582,556	166,582,737	cg13032463	166,582,585
10	177	6	166,582,640	166,582,813	NA	NA
10	178	6	166,582,728	166,582,926	NA	NA
10	179	6	166,582,855	166,583,016	NA	NA
10	180	6	166,582,921	166,583,120	NA	NA
10	181	6	166,583,061	166,583,257	cg01392518	166,583,226
11	182	7	4,921,281	4,921,439	NA	NA
11	183	7	4,921,386	4,921,455	NA	NA
11	184	7	4,921,407	4,921,560	NA	NA
11	185	7	4,921,498	4,921,597	NA	NA
11	186	7	4,921,528	4,921,720	NA	NA
11	187	7	4,921,633	4,921,823	NA	NA
11	188	7	4,921,743	4,921,918	cg04816699	4,921,827
11	189	7	4,921,862	4,922,029	cg25266895	4,921,975
11	190	7	4,921,901	4,922,082	cg25266895	4,921,975
11	190	7	4,921,901	4,922,082	cg03229627	4,922,039
11	191	7	4,922,003	4,922,175	cg03229627	4,922,039
11	192	7	4,922,060	4,922,254	cg10884288	4,922,196
11	193	7	4,922,199	4,922,325	NA	NA
11	194	7	4,922,283	4,922,454	NA	NA
11	195	7	4,922,377	4,922,555	NA	NA
11	196	7	4,922,429	4,922,604	cg26013579	4,922,565
11	197	7	4,922,537	4,922,708	cg26013579	4,922,565
11	197	7	4,922,537	4,922,708	cg06223466	4,922,708
11	198	7	4,922,605	4,922,757	cg06223466	4,922,708
11	199	7	4,922,685	4,922,882	cg06223466	4,922,708
11	200	7	4,922,735	4,922,917	NA	NA
11	201	7	4,922,848	4,922,945	cg03638172	4,922,944
11	202	7	4,922,897	4,923,084	cg03638172	4,922,944
12	203	7	42,276,948	42,277,117	cg19603847	42,276,981
12	203	7	42,276,948	42,277,117	cg10569039	42,277,044
12	203	7	42,276,948	42,277,117	cg11749583	42,277,066
12	203	7	42,276,948	42,277,117	cg25846074	42,277,071
12	204	7	42,277,073	42,277,260	NA	NA
12	205	7	42,277,203	42,277,402	cg06500654	42,277,347
12	205	7	42,277,203	42,277,402	cg07191743	42,277,375
12	205	7	42,277,203	42,277,402	cg19208079	42,277,394
12	206	7	42,277,273	42,277,469	cg06500654	42,277,347
12	206	7	42,277,273	42,277,469	cg07191743	42,277,375
12	206	7	42,277,273	42,277,469	cg19208079	42,277,394
12	206	7	42,277,273	42,277,469	cg03366925	42,277,410
12	207	7	42,277,442	42,277,628	NA	NA
12	208	7	42,277,447	42,277,637	NA	NA
12	209	7	42,277,607	42,277,681	NA	NA
12	210	7	42,277,609	42,277,690	NA	NA
12	211	7	42,277,649	42,277,748	NA	NA
12	212	7	42,277,690	42,277,876	cg25726664	42,277,807
12	213	7	42,277,788	42,277,971	cg25726664	42,277,807
12	213	7	42,277,788	42,277,971	cg02483271	42,277,950
12	214	7	42,277,889	42,278,043	cg02483271	42,277,950
12	215	7	42,277,955	42,278,141	cg17588800	42,278,067
12	215	7	42,277,955	42,278,141	cg24497732	42,278,077
12	215	7	42,277,955	42,278,141	cg14396995	42,278,089
12	215	7	42,277,955	42,278,141	cg26557756	42,278,096
12	216	7	42,278,026	42,278,184	cg17588800	42,278,067
12	216	7	42,278,026	42,278,184	cg24497732	42,278,077
12	216	7	42,278,026	42,278,184	cg14396995	42,278,089
12	216	7	42,278,026	42,278,184	cg26557756	42,278,096
12	217	7	42,278,118	42,278,249	NA	NA
12	218	7	42,278,151	42,278,307	NA	NA
12	219	7	42,278,227	42,278,421	NA	NA
12	220	7	42,278,359	42,278,533	NA	NA
12	221	7	42,278,440	42,278,639	NA	NA
13	222	7	148,394,357	148,394,495	NA	NA
13	223	7	148,394,371	148,394,566	cg18597411	148,394,542
13	224	7	148,394,476	148,394,625	cg18597411	148,394,542
13	225	7	148,394,550	148,394,691	NA	NA
13	226	7	148,394,646	148,394,761	NA	NA
13	227	7	148,394,675	148,394,871	cg03789645	148,394,842
13	228	7	148,394,754	148,394,936	cg03789645	148,394,842
13	228	7	148,394,754	148,394,936	cg11109845	148,394,888
13	229	7	148,394,851	148,395,034	cg11109845	148,394,888
13	229	7	148,394,851	148,395,034	cg06899186	148,394,994
13	230	7	148,394,913	148,395,110	cg06899186	148,394,994
13	231	7	148,395,050	148,395,222	cg12592716	148,395,181
13	232	7	148,395,134	148,395,331	cg12592716	148,395,181
13	233	7	148,395,219	148,395,413	NA	NA

139	150	5	140,855,443	140,855,547	cg02780295	140,855,463
139	150	5	140,855,443	140,855,547	cg15124968	140,855,469
139	150	5	140,855,443	140,855,547	cg13993336	140,855,482
139	150	5	140,855,443	140,855,547	cg23359276	140,855,504
139	150	5	140,855,443	140,855,547	cg02430347	140,855,547
139	151	5	140,855,522	140,855,681	cg02430347	140,855,547
139	151	5	140,855,522	140,855,681	cg00281842	140,855,562
139	151	5	140,855,522	140,855,681	cg10858746	140,855,582
140	152	5	148,803,341	148,803,451	cg18055067	148,803,404
141	153	5	149,682,211	149,682,341	cg25780607	149,682,233
142	154	5	149,792,305	149,792,419	cg26129545	149,792,330
142	154	5	149,792,305	149,792,419	cg01601628	149,792,348
142	154	5	149,792,305	149,792,419	cg14484145	149,792,371
142	154	5	149,792,305	149,792,419	cg24548564	149,792,390
143	155	5	170,210,555	170,210,720	cg27129755	170,210,586
143	155	5	170,210,555	170,210,720	cg22635402	170,210,611
143	155	5	170,210,555	170,210,720	cg23181170	170,210,672
144	156	5	172,260,548	172,260,692	cg02855432	172,260,641
145	157	5	173,213,608	173,213,766	cg02640809	173,213,710
146	158	5	177,365,652	177,365,823	cg20986443	177,365,747
147	159	5	179,340,067	179,340,221	cg05624954	179,340,170
148	160	5	179,402,162	179,402,339	cg04265672	179,402,240
149	161	6	291,802	291,943	cg21548813	291,882
149	161	6	291,802	291,943	cg03395511	291,903
149	161	6	291,802	291,943	cg15383120	291,909
150	162	6	30,140,276	30,140,407	cg05563595	30,140,305
150	162	6	30,140,276	30,140,407	cg15671996	30,140,325
150	162	6	30,140,276	30,140,407	cg24677444	30,140,366
150	162	6	30,140,276	30,140,407	cg02668405	30,140,372
150	163	6	30,140,380	30,140,578	cg17527819	30,140,409
150	163	6	30,140,380	30,140,578	cg03261948	30,140,417
151	164	6	30,851,362	30,851,481	cg02695062	30,851,417
152	165	6	31,696,260	31,696,361	cg08509383	31,696,298
152	165	6	31,696,260	31,696,361	cg16359915	31,696,309
152	165	6	31,696,260	31,696,361	cg19008099	31,696,317
152	165	6	31,696,260	31,696,361	cg23348155	31,696,319
152	165	6	31,696,260	31,696,361	cg14293129	31,696,333
153	166	6	32,042,976	32,043,086	cg14173662	32,043,029
154	167	6	34,123,491	34,123,596	cg00538717	34,123,544
154	167	6	34,123,491	34,123,596	cg26552572	34,123,547
155	168	6	42,167,120	42,167,294	cg20470945	42,167,188
156	169	6	62,996,644	62,996,758	cg23926793	62,996,664
156	169	6	62,996,644	62,996,758	cg02217159	62,996,697
156	169	6	62,996,644	62,996,758	cg00578220	62,996,702
157	170	6	108,439,447	108,439,582	cg12940104	108,439,511
157	170	6	108,439,447	108,439,582	cg22727304	108,439,520
158	171	6	110,679,251	110,679,410	cg25146017	110,679,400
159	172	6	117,737,021	117,737,200	cg13994196	117,737,102
160	173	6	122,007,717	122,007,833	NA	NA
160	174	6	122,007,801	122,007,951	cg14827083	122,007,834
161	175	6	129,478,913	129,479,064	cg27439042	129,478,919
161	175	6	129,478,913	129,479,064	cg19625507	129,478,967
161	175	6	129,478,913	129,479,064	cg27192990	129,479,024
162	176	6	131,480,909	131,481,072	cg12210836	131,481,003
163	177	6	137,242,247	137,242,406	cg10242496	137,242,316
164	178	6	142,277,604	142,277,725	cg16797473	142,277,658
165	179	6	145,392,227	145,392,356	cg02274934	145,392,278
166	180	6	149,367,220	149,367,355	cg06827883	149,367,279
167	181	6	151,645,320	151,645,505	cg19995197	151,645,438
168	182	6	152,128,475	152,128,667	cg21950534	152,128,483
168	182	6	152,128,475	152,128,667	cg11813455	152,128,515
168	182	6	152,128,475	152,128,667	cg24900983	152,128,528
168	182	6	152,128,475	152,128,667	cg05171584	152,128,535
168	182	6	152,128,475	152,128,667	cg23467008	152,128,537
168	182	6	152,128,475	152,128,667	cg22839866	152,128,584
168	182	6	152,128,475	152,128,667	cg23009221	152,128,588
168	182	6	152,128,475	152,128,667	cg13612689	152,128,634
168	183	6	152,128,556	152,128,737	cg22839866	152,128,584
168	183	6	152,128,556	152,128,737	cg23009221	152,128,588
168	183	6	152,128,556	152,128,737	cg13612689	152,128,634
168	183	6	152,128,556	152,128,737	cg27316393	152,128,675
169	184	6	152,129,723	152,129,854	cg02285263	152,129,749
169	184	6	152,129,723	152,129,854	cg02720618	152,129,791
170	185	6	168,517,646	168,517,843	cg18018913	168,517,728
170	185	6	168,517,646	168,517,843	cg05301124	168,517,769
170	185	6	168,517,646	168,517,843	cg04213854	168,517,792
171	186	6	168,720,445	168,720,626	cg24966702	168,720,476
171	186	6	168,720,445	168,720,626	cg11238048	168,720,487
171	186	6	168,720,445	168,720,626	cg04053392	168,720,505
172	187	7	1,272,352	1,272,551	cg21543987	1,272,486
172	187	7	1,272,352	1,272,551	cg08507422	1,272,512
172	187	7	1,272,352	1,272,551	cg20870512	1,272,515
172	187	7	1,272,352	1,272,551	cg21158633	1,272,526
172	187	7	1,272,352	1,272,551	cg05157140	1,272,545
173	188	7	2,394,229	2,394,415	cg23289794	2,394,357

13	234	7	148,395,352	148,395,471	cg00113208	148,395,441
13	235	7	148,395,403	148,395,561	cg00113208	148,395,441
13	236	7	148,395,452	148,395,627	NA	NA
13	237	7	148,395,717	148,395,837	NA	NA
13	238	7	148,395,818	148,396,009	NA	NA
14	239	7	150,710,593	150,710,788	cg09519327	150,710,730
14	240	7	150,710,646	150,710,835	cg09519327	150,710,730
14	241	7	150,710,778	150,710,930	NA	NA
14	242	7	150,710,908	150,711,091	NA	NA
14	243	7	150,710,909	150,711,091	NA	NA
14	244	7	150,711,064	150,711,261	cg02027878	150,711,138
14	245	7	150,711,167	150,711,357	NA	NA
14	246	7	150,711,243	150,711,441	NA	NA
14	247	7	150,711,380	150,711,576	cg25025646	150,711,449
14	248	7	150,711,496	150,711,690	NA	NA
14	249	7	150,711,621	150,711,800	NA	NA
14	250	7	150,711,655	150,711,851	NA	NA
14	251	7	150,711,864	150,712,045	NA	NA
14	252	7	150,711,948	150,712,122	NA	NA
14	253	7	150,712,055	150,712,215	NA	NA
14	254	7	150,712,127	150,712,308	NA	NA
15	255	8	119,124,470	119,124,668	NA	NA
15	256	8	119,124,577	119,124,716	NA	NA
15	257	8	119,124,653	119,124,847	cg16374656	119,124,790
15	258	8	119,124,762	119,124,926	cg16374656	119,124,790
15	259	8	119,124,843	119,125,039	NA	NA
15	260	8	119,124,928	119,125,125	NA	NA
15	261	8	119,125,040	119,125,238	NA	NA
15	262	8	119,125,123	119,125,322	NA	NA
15	263	8	119,125,264	119,125,433	cg15694987	119,125,345
15	264	8	119,125,310	119,125,462	cg15694987	119,125,345
15	265	8	119,125,400	119,125,584	NA	NA
15	266	8	119,125,440	119,125,621	NA	NA
15	267	8	119,125,566	119,125,693	NA	NA
15	268	8	119,125,602	119,125,776	NA	NA
15	269	8	119,125,678	119,125,872	NA	NA
15	270	8	119,125,794	119,125,986	NA	NA
15	271	8	119,125,849	119,126,046	NA	NA
15	272	8	119,125,996	119,126,106	NA	NA
15	273	8	119,126,015	119,126,211	NA	NA
16	274	8	121,137,457	121,137,651	cg23196831	121,137,537
16	274	8	121,137,457	121,137,651	cg02964031	121,137,618
16	275	8	121,137,541	121,137,732	cg02964031	121,137,618
16	276	8	121,137,622	121,137,797	cg23158731	121,137,734
16	277	8	121,137,713	121,137,845	cg23158731	121,137,734
16	278	8	121,137,791	121,137,988	cg26626663	121,137,879
16	279	8	121,137,921	121,138,049	NA	NA
16	280	8	121,137,979	121,138,117	NA	NA
16	281	8	121,138,029	121,138,192	cg12065840	121,138,133
16	282	8	121,138,143	121,138,266	cg23586322	121,138,232
16	283	8	121,138,170	121,138,314	cg23586322	121,138,232
16	284	8	121,138,241	121,138,439	cg23281803	121,138,317
16	285	8	121,138,382	121,138,581	NA	NA
16	286	8	121,138,474	121,138,641	NA	NA
16	287	8	121,138,575	121,138,743	NA	NA
16	288	8	121,138,663	121,138,862	NA	NA
16	289	8	121,138,787	121,138,960	NA	NA
16	290	8	121,138,865	121,138,992	NA	NA
16	291	8	121,138,927	121,139,116	NA	NA
16	292	8	121,139,033	121,139,174	NA	NA
17	293	10	70,318,760	70,318,959	NA	NA
17	294	10	70,318,871	70,319,006	NA	NA
17	295	10	70,318,952	70,319,132	NA	NA
17	296	10	70,318,978	70,319,177	NA	NA
17	297	10	70,319,106	70,319,281	NA	NA
17	298	10	70,319,154	70,319,353	NA	NA
17	299	10	70,319,258	70,319,418	NA	NA
17	300	10	70,319,354	70,319,552	NA	NA
17	301	10	70,319,454	70,319,621	NA	NA
17	302	10	70,319,535	70,319,668	cg23602092	70,319,645
17	303	10	70,319,603	70,319,716	cg23602092	70,319,645
17	304	10	70,319,648	70,319,829	cg22876739	70,319,774
17	304	10	70,319,648	70,319,829	cg02952701	70,319,783
17	305	10	70,319,727	70,319,926	cg22876739	70,319,774
17	305	10	70,319,727	70,319,926	cg02952701	70,319,783
17	306	10	70,319,866	70,320,033	cg00128195	70,319,998
17	307	10	70,319,903	70,320,102	cg00128195	70,319,998
17	307	10	70,319,903	70,320,102	cg07669489	70,320,037
17	307	10	70,319,903	70,320,102	cg10413954	70,320,039
17	307	10	70,319,903	70,320,102	cg21948169	70,320,051
17	307	10	70,319,903	70,320,102	cg05400741	70,320,073
17	308	10	70,319,999	70,320,128	cg07669489	70,320,037
17	308	10	70,319,999	70,320,128	cg10413954	70,320,039
17	308	10	70,319,999	70,320,128	cg21948169	70,320,051
17	308	10	70,319,999	70,320,128	cg05400741	70,320,073

173	188	7	2,394,229	2,394,415	cg05083358	2,394,359
173	188	7	2,394,229	2,394,415	cg02637781	2,394,389
173	188	7	2,394,229	2,394,415	cg25053232	2,394,393
174	189	7	3,025,552	3,025,709	cg15025536	3,025,634
174	189	7	3,025,552	3,025,709	cg23352157	3,025,689
175	190	7	26,206,522	26,206,630	cg07945582	26,206,579
176	191	7	27,154,967	27,155,092	cg16748008	27,155,002
176	191	7	27,154,967	27,155,092	cg16406967	27,155,036
176	191	7	27,154,967	27,155,092	cg18680977	27,155,039
177	192	7	27,170,172	27,170,371	cg22997113	27,170,241
177	192	7	27,170,172	27,170,371	cg04317399	27,170,313
178	193	7	44,801,450	44,801,643	cg26124968	44,801,571
178	193	7	44,801,450	44,801,643	cg20910408	44,801,589
179	194	7	49,894,535	49,894,721	cg18023455	49,894,664
180	195	7	50,341,147	50,341,264	cg08052546	50,341,210
181	196	7	56,419,045	56,419,183	cg05864011	56,419,094
182	197	7	56,551,532	56,551,725	cg26565223	56,551,590
182	197	7	56,551,532	56,551,725	cg26733975	56,551,640
183	198	7	63,640,953	63,641,100	cg19627238	63,641,030
184	199	7	71,682,241	71,682,374	cg09361748	71,682,306
185	200	7	73,667,344	73,667,502	cg04577162	73,667,397
186	201	7	75,994,025	75,994,129	cg11549025	75,994,082
187	202	7	92,672,414	92,672,591	cg03973379	92,672,484
188	203	7	93,519,111	93,519,270	cg18024479	93,519,220
189	204	7	93,519,734	93,519,921	cg20230721	93,519,855
189	204	7	93,519,734	93,519,921	cg23141855	93,519,892
190	205	7	93,520,129	93,520,314	cg26739865	93,520,172
190	205	7	93,520,129	93,520,314	cg22441533	93,520,175
190	205	7	93,520,129	93,520,314	cg14377593	93,520,183
190	205	7	93,520,129	93,520,314	cg15649801	93,520,269
190	205	7	93,520,129	93,520,314	cg10539069	93,520,275
190	205	7	93,520,129	93,520,314	cg24084681	93,520,288
190	206	7	93,520,218	93,520,417	cg15649801	93,520,269
190	206	7	93,520,218	93,520,417	cg10539069	93,520,275
190	206	7	93,520,218	93,520,417	cg24084681	93,520,288
190	206	7	93,520,218	93,520,417	cg07380959	93,520,323
190	206	7	93,520,218	93,520,417	cg03333330	93,520,346
191	207	7	97,739,636	97,739,763	cg10412943	97,739,696
192	208	7	107,222,998	107,223,149	cg14288925	107,223,076
193	209	7	112,459,079	112,459,252	cg15723346	112,459,177
194	210	7	137,528,877	137,529,011	cg18249027	137,528,924
195	211	7	138,229,827	138,230,013	cg12743416	138,229,989
195	212	7	138,229,962	138,230,082	cg12743416	138,229,989
196	213	7	140,218,088	140,218,193	cg06310157	140,218,138
197	214	7	149,470,177	149,470,341	cg23355674	149,470,284
197	214	7	149,470,177	149,470,341	cg02372712	149,470,295
197	214	7	149,470,177	149,470,341	cg10819771	149,470,335
197	215	7	149,470,296	149,470,417	cg10819771	149,470,335
197	215	7	149,470,296	149,470,417	cg16803141	149,470,343
198	216	7	151,505,060	151,505,235	cg22528270	151,505,116
199	217	7	154,001,944	154,002,123	cg10552126	154,002,064
200	218	7	157,476,849	157,477,048	cg20842326	157,476,887
200	218	7	157,476,849	157,477,048	cg02030008	157,476,930
201	219	7	158,893,820	158,894,046	cg03754402	158,893,862
201	219	7	158,893,820	158,894,046	cg18278817	158,894,003
202	220	8	1,940,449	1,940,648	cg01648999	1,940,523
203	221	8	2,820,759	2,820,890	cg12027248	2,820,857
204	222	8	3,316,775	3,316,942	cg22726414	3,316,819
204	222	8	3,316,775	3,316,942	cg00552087	3,316,893
205	223	8	6,666,345	6,666,528	cg26470958	6,666,420
206	224	8	23,102,019	23,102,151	cg17048073	23,102,091
207	225	8	42,010,120	42,010,298	cg20603637	42,010,142
207	225	8	42,010,120	42,010,298	cg03361810	42,010,162
207	225	8	42,010,120	42,010,298	cg07490776	42,010,169
208	226	8	55,087,259	55,087,372	cg06042504	55,087,323
209	227	8	56,677,694	56,677,802	cg22525251	56,677,752
210	228	8	62,606,110	62,606,265	cg14266248	62,606,211
211	229	8	65,496,060	65,496,215	cg07205627	65,496,126
212	230	8	67,874,084	67,874,269	cg20980783	67,874,178
212	230	8	67,874,084	67,874,269	cg26618965	67,874,206
212	230	8	67,874,084	67,874,269	cg00909646	67,874,250
213	231	8	74,878,432	74,878,608	cg17316966	74,878,476
214	232	8	87,586,856	87,587,031	cg20794957	87,586,952
215	233	8	97,167,711	97,167,870	cg16290866	97,167,768
216	234	8	121,457,722	121,457,881	cg26609386	121,457,799
216	234	8	121,457,722	121,457,881	cg26205175	121,457,826
216	234	8	121,457,722	121,457,881	cg09948425	121,457,834
217	235	8	124,193,729	124,193,888	cg02715629	124,193,817
218	236	8	144,162,695	144,162,861	cg04017479	144,162,802
219	237	8	144,872,770	144,872,931	cg09541794	144,872,852
220	238	9	13,278,424	13,278,544	cg10899099	13,278,512
220	239	9	13,278,482	13,278,680	cg10899099	13,278,512
220	239	9	13,278,482	13,278,680	cg14655855	13,278,626
221	240	9	33,446,962	33,447,112	cg02716826	33,447,032
222	241	9	139,082,858	139,083,048	cg14046688	139,082,923

17	309	10	70,320,076	70,320,272	cg03651138	70,320,213
17	310	10	70,320,138	70,320,336	cg03651138	70,320,213
17	311	10	70,320,244	70,320,441	NA	NA
17	312	10	70,320,391	70,320,529	cg19439331	70,320,470
18	313	10	94,831,753	94,831,931	NA	NA
18	314	10	94,831,879	94,832,032	NA	NA
18	315	10	94,831,899	94,832,098	cg16169084	94,832,033
18	316	10	94,832,006	94,832,160	cg16169084	94,832,033
18	316	10	94,832,006	94,832,160	cg18080906	94,832,106
18	317	10	94,832,077	94,832,219	cg18080906	94,832,106
18	318	10	94,832,148	94,832,338	NA	NA
18	319	10	94,832,250	94,832,426	NA	NA
18	320	10	94,832,327	94,832,503	NA	NA
18	321	10	94,832,455	94,832,558	NA	NA
18	322	10	94,832,473	94,832,670	cg04082461	94,832,569
18	322	10	94,832,473	94,832,670	cg11310684	94,832,631
18	323	10	94,832,573	94,832,741	cg11310684	94,832,631
18	324	10	94,832,651	94,832,802	NA	NA
18	325	10	94,832,729	94,832,924	NA	NA
18	326	10	94,832,778	94,832,960	NA	NA
18	327	10	94,832,909	94,833,087	cg14539900	94,833,042
18	327	10	94,832,909	94,833,087	cg10145436	94,833,054
18	328	10	94,833,011	94,833,177	cg14539900	94,833,042
18	328	10	94,833,011	94,833,177	cg10145436	94,833,054
18	328	10	94,833,011	94,833,177	cg04609350	94,833,102
18	328	10	94,833,011	94,833,177	cg02078870	94,833,108
18	328	10	94,833,011	94,833,177	cg24849846	94,833,158
18	329	10	94,833,064	94,833,262	cg04609350	94,833,102
18	329	10	94,833,064	94,833,262	cg02078870	94,833,108
18	329	10	94,833,064	94,833,262	cg24849846	94,833,158
18	329	10	94,833,064	94,833,262	cg03099492	94,833,214
18	330	10	94,833,193	94,833,372	cg03099492	94,833,214
18	331	10	94,833,245	94,833,410	NA	NA
18	332	10	94,833,357	94,833,556	cg26652906	94,833,520
18	332	10	94,833,357	94,833,556	cg19453618	94,833,548
19	333	10	100,027,671	100,027,803	NA	NA
19	334	10	100,027,707	100,027,838	NA	NA
19	335	10	100,027,787	100,027,908	NA	NA
19	336	10	100,027,872	100,028,017	cg24163616	100,027,958
19	336	10	100,027,872	100,028,017	cg09335911	100,027,962
19	337	10	100,027,883	100,028,018	cg24163616	100,027,958
19	337	10	100,027,883	100,028,018	cg09335911	100,027,962
19	338	10	100,028,238	100,028,368	cg04677227	100,028,320
19	339	10	100,028,320	100,028,477	cg11632906	100,028,468
19	340	10	100,028,347	100,028,546	cg11632906	100,028,468
19	340	10	100,028,347	100,028,546	cg07146435	100,028,499
19	341	10	100,028,460	100,028,621	cg11632906	100,028,468
19	341	10	100,028,460	100,028,621	cg07146435	100,028,499
19	342	10	100,028,519	100,028,704	NA	NA
19	343	10	100,028,605	100,028,745	NA	NA
19	344	10	100,028,673	100,028,854	NA	NA
19	345	10	100,028,730	100,028,893	NA	NA
19	346	10	100,028,835	100,029,027	cg04521765	100,028,929
19	347	10	100,028,942	100,029,119	NA	NA
19	348	10	100,029,054	100,029,229	NA	NA
19	349	10	100,029,129	100,029,294	NA	NA
19	350	10	100,029,217	100,029,382	NA	NA
20	351	10	102,894,121	102,894,320	cg25266629	102,894,148
20	351	10	102,894,121	102,894,320	cg04273871	102,894,227
20	352	10	102,894,266	102,894,438	cg07915434	102,894,342
20	353	10	102,894,351	102,894,547	cg07416656	102,894,441
20	354	10	102,894,491	102,894,648	cg08487063	102,894,639
20	355	10	102,894,609	102,894,793	cg08487063	102,894,639
20	356	10	102,894,618	102,894,814	cg08487063	102,894,639
20	357	10	102,894,763	102,894,881	NA	NA
20	358	10	102,894,774	102,894,892	NA	NA
20	359	10	102,894,862	102,895,008	NA	NA
20	360	10	102,894,870	102,895,019	NA	NA
20	361	10	102,894,983	102,895,181	cg14861089	102,895,043
20	361	10	102,894,983	102,895,181	cg23340017	102,895,133
20	362	10	102,895,060	102,895,211	cg23340017	102,895,133
20	363	10	102,895,141	102,895,333	NA	NA
20	364	10	102,895,235	102,895,416	NA	NA
20	365	10	102,895,314	102,895,513	NA	NA
20	366	10	102,895,406	102,895,571	NA	NA
20	367	10	102,895,479	102,895,646	NA	NA
20	368	10	102,895,550	102,895,733	cg14461582	102,895,708
20	369	10	102,895,626	102,895,785	cg14461582	102,895,708
20	370	10	102,895,712	102,895,908	NA	NA
21	371	11	44,116,085	44,116,284	NA	NA
21	372	11	44,116,181	44,116,303	NA	NA
21	373	11	44,116,337	44,116,460	NA	NA
21	374	11	44,116,418	44,116,601	NA	NA
21	375	11	44,116,488	44,116,665	NA	NA
21	376	11	44,116,588	44,116,787	NA	NA

223	242	10	7,451,243	7,451,370	cg09060610	7,451,314
224	243	10	8,090,822	8,091,008	cg25954627	8,090,846
224	243	10	8,090,822	8,091,008	cg27542609	8,090,924
225	244	10	16,562,539	16,562,708	cg24037897	16,562,626
226	245	10	25,306,296	25,306,452	cg06763909	25,306,365
227	246	10	54,071,043	54,071,202	cg12799790	54,071,134
228	247	10	69,370,035	69,370,219	cg03333602	69,370,143
229	248	10	74,451,593	74,451,748	cg15871229	74,451,622
229	248	10	74,451,593	74,451,748	cg23879147	74,451,659
230	249	10	102,896,764	102,896,925	cg20120208	102,896,869
231	250	10	104,503,587	104,503,733	cg05855489	104,503,620
231	250	10	104,503,587	104,503,733	cg10107304	104,503,664
231	250	10	104,503,587	104,503,733	cg12846190	104,503,666
231	250	10	104,503,587	104,503,733	cg03575163	104,503,671
231	250	10	104,503,587	104,503,733	cg14838188	104,503,687
232	251	10	105,127,555	105,127,701	cg11667101	105,127,581
232	251	10	105,127,555	105,127,701	cg18586343	105,127,615
232	251	10	105,127,555	105,127,701	cg20684973	105,127,632
232	251	10	105,127,555	105,127,701	cg18732587	105,127,670
232	251	10	105,127,555	105,127,701	cg25713684	105,127,687
232	251	10	105,127,555	105,127,701	cg16500454	105,127,701
232	252	10	105,127,651	105,127,750	cg18732587	105,127,670
232	252	10	105,127,651	105,127,750	cg25713684	105,127,687
232	252	10	105,127,651	105,127,750	cg16500454	105,127,701
233	253	10	115,998,910	115,999,052	cg20212699	115,998,991
233	253	10	115,998,910	115,999,052	cg16811695	115,999,009
233	253	10	115,998,910	115,999,052	cg13084429	115,999,021
234	254	10	119,000,960	119,001,119	cg00512279	119,000,991
234	254	10	119,000,960	119,001,119	cg19721867	119,001,066
235	255	10	125,651,336	125,651,478	cg14676407	125,651,370
235	255	10	125,651,336	125,651,478	cg27003782	125,651,391
236	256	10	125,853,103	125,853,296	cg00660608	125,853,219
236	256	10	125,853,103	125,853,296	cg03084724	125,853,232
237	257	10	134,053,845	134,054,025	cg17555624	134,053,894
237	257	10	134,053,845	134,054,025	cg00355188	134,053,962
238	258	10	134,645,908	134,646,045	cg23908771	134,645,960
238	258	10	134,645,908	134,646,045	cg01391063	134,645,978
238	258	10	134,645,908	134,646,045	cg13166642	134,645,995
239	259	10	134,790,827	134,790,986	cg16444701	134,790,855
239	259	10	134,790,827	134,790,986	cg11194118	134,790,922
240	260	10	135,153,924	135,154,106	cg17247026	135,153,937
240	260	10	135,153,924	135,154,106	cg24748548	135,153,961
240	260	10	135,153,924	135,154,106	cg06922248	135,153,972
241	261	11	1,965,084	1,965,199	cg03652715	1,965,140
242	262	11	2,162,455	2,162,647	cg17300736	2,162,475
242	262	11	2,162,455	2,162,647	cg11701022	2,162,478
242	262	11	2,162,455	2,162,647	cg01368777	2,162,483
242	262	11	2,162,455	2,162,647	cg21237591	2,162,510
242	262	11	2,162,455	2,162,647	cg22225943	2,162,536
242	262	11	2,162,455	2,162,647	cg08014499	2,162,545
242	262	11	2,162,455	2,162,647	cg05203776	2,162,582
242	262	11	2,162,455	2,162,647	cg20088847	2,162,616
243	263	11	2,555,503	2,555,641	cg05898618	2,555,576
244	264	11	8,190,671	8,190,785	cg04041669	8,190,690
245	265	11	10,764,560	10,764,675	cg09645818	10,764,592
246	266	11	26,968,478	26,968,603	cg10327008	26,968,547
247	267	11	30,929,165	30,929,324	cg21758962	30,929,224
248	268	11	31,845,283	31,845,464	cg08047827	31,845,331
248	268	11	31,845,283	31,845,464	cg24701575	31,845,389
249	269	11	32,912,661	32,912,798	cg01481251	32,912,719
250	270	11	35,229,399	35,229,560	cg15239179	35,229,448
251	271	11	58,385,876	58,386,014	cg12027899	58,385,947
252	272	11	61,258,049	61,258,160	cg13236257	61,258,107
253	273	11	62,414,701	62,414,860	cg16260700	62,414,786
254	274	11	63,839,416	63,839,615	cg09705456	63,839,479
254	274	11	63,839,416	63,839,615	cg27456707	63,839,511
254	274	11	63,839,416	63,839,615	cg03460534	63,839,528
255	275	11	64,397,695	64,397,832	cg27466845	64,397,734
255	275	11	64,397,695	64,397,832	cg21660452	64,397,807
256	276	11	66,361,032	66,361,187	cg06456738	66,361,081
257	277	11	69,197,133	69,197,266	cg25169679	69,197,217
258	278	11	72,213,943	72,214,122	cg11958933	72,213,993
259	279	11	94,474,270	94,474,454	cg06233455	94,474,371
260	280	11	103,858,835	103,858,945	cg18274315	103,858,893
261	281	11	115,530,953	115,531,099	cg02256049	115,531,001
261	281	11	115,530,953	115,531,099	cg21540621	115,531,033
262	282	11	116,897,494	116,897,653	cg23684410	116,897,558
263	283	11	125,933,158	125,933,353	cg09544918	125,933,187
263	283	11	125,933,158	125,933,353	cg23021551	125,933,215
263	283	11	125,933,158	125,933,353	cg05631262	125,933,228
263	283	11	125,933,158	125,933,353	cg09059511	125,933,236
263	283	11	125,933,158	125,933,353	cg26193555	125,933,258
263	283	11	125,933,158	125,933,353	cg05462819	125,933,319
264	284	11	128,813,366	128,813,528	cg03996398	128,813,442
264	284	11	128,813,366	128,813,528	cg08432383	128,813,451

21	377	11	44,116,668	44,116,867	NA	NA
21	378	11	44,116,751	44,116,905	NA	NA
21	379	11	44,116,848	44,116,938	cg08133496	44,116,911
21	380	11	44,116,852	44,116,987	cg08133496	44,116,911
21	380	11	44,116,852	44,116,987	cg02699915	44,116,952
21	381	11	44,116,918	44,117,076	cg02699915	44,116,952
21	381	11	44,116,918	44,117,076	cg21061731	44,116,994
21	381	11	44,116,918	44,117,076	cg24612772	44,117,021
21	381	11	44,116,918	44,117,076	cg03022937	44,117,063
21	382	11	44,116,978	44,117,155	cg21061731	44,116,994
21	382	11	44,116,978	44,117,155	cg24612772	44,117,021
21	382	11	44,116,978	44,117,155	cg03022937	44,117,063
21	383	11	44,117,055	44,117,160	cg03022937	44,117,063
21	384	11	44,117,131	44,117,304	cg02620470	44,117,187
21	384	11	44,117,131	44,117,304	cg12640937	44,117,231
21	384	11	44,117,131	44,117,304	cg02050903	44,117,243
21	385	11	44,117,534	44,117,679	cg06536200	44,117,561
21	386	11	44,117,610	44,117,796	NA	NA
22	387	11	72,928,287	72,928,484	NA	NA
22	388	11	72,928,406	72,928,588	NA	NA
22	389	11	72,928,536	72,928,735	NA	NA
22	390	11	72,928,634	72,928,789	NA	NA
22	391	11	72,928,714	72,928,863	NA	NA
22	392	11	72,928,812	72,928,992	cg26761618	72,928,926
22	393	11	72,928,947	72,929,124	cg10287137	72,929,054
22	393	11	72,928,947	72,929,124	cg16872520	72,929,124
22	394	11	72,928,972	72,929,163	cg10287137	72,929,054
22	394	11	72,928,972	72,929,163	cg16872520	72,929,124
22	394	11	72,928,972	72,929,163	cg11110643	72,929,135
22	395	11	72,929,098	72,929,167	cg16872520	72,929,124
22	395	11	72,929,098	72,929,167	cg11110643	72,929,135
22	395	11	72,929,098	72,929,167	cg24235904	72,929,167
22	396	11	72,929,125	72,929,307	cg11110643	72,929,135
22	396	11	72,929,125	72,929,307	cg24235904	72,929,167
22	396	11	72,929,125	72,929,307	cg26046204	72,929,170
22	396	11	72,929,125	72,929,307	cg23757899	72,929,173
22	396	11	72,929,125	72,929,307	cg26571618	72,929,307
22	397	11	72,929,126	72,929,307	cg11110643	72,929,135
22	397	11	72,929,126	72,929,307	cg24235904	72,929,167
22	397	11	72,929,126	72,929,307	cg26046204	72,929,170
22	397	11	72,929,126	72,929,307	cg23757899	72,929,173
22	397	11	72,929,126	72,929,307	cg26571618	72,929,307
22	398	11	72,929,283	72,929,379	cg26571618	72,929,307
22	398	11	72,929,283	72,929,379	cg14318946	72,929,312
22	398	11	72,929,283	72,929,379	cg01875418	72,929,368
22	399	11	72,929,329	72,929,484	cg01875418	72,929,368
22	399	11	72,929,329	72,929,484	cg20718724	72,929,382
22	399	11	72,929,329	72,929,484	cg26013434	72,929,394
22	400	11	72,929,358	72,929,528	cg01875418	72,929,368
22	400	11	72,929,358	72,929,528	cg20718724	72,929,382
22	400	11	72,929,358	72,929,528	cg26013434	72,929,394
22	401	11	72,929,484	72,929,587	cg00334798	72,929,553
22	402	11	72,929,569	72,929,738	NA	NA
22	403	11	72,929,658	72,929,770	NA	NA
22	404	11	72,929,714	72,929,841	NA	NA
22	405	11	72,929,750	72,929,934	cg04388983	72,929,844
22	406	11	72,929,813	72,929,975	cg04388983	72,929,844
23	407	11	74,177,253	74,177,452	NA	NA
23	408	11	74,177,372	74,177,545	NA	NA
23	409	11	74,177,437	74,177,635	NA	NA
23	410	11	74,177,528	74,177,687	NA	NA
23	411	11	74,177,620	74,177,763	NA	NA
23	412	11	74,177,668	74,177,858	NA	NA
23	413	11	74,177,799	74,177,998	NA	NA
23	414	11	74,177,913	74,178,070	NA	NA
23	415	11	74,177,978	74,178,144	cg23189044	74,178,114
23	416	11	74,178,040	74,178,239	cg23189044	74,178,114
23	417	11	74,178,127	74,178,290	cg04507515	74,178,268
23	418	11	74,178,214	74,178,404	cg04507515	74,178,268
23	419	11	74,178,359	74,178,446	NA	NA
23	420	11	74,178,388	74,178,545	cg05789714	74,178,462
23	421	11	74,178,409	74,178,605	cg05789714	74,178,462
23	422	11	74,178,527	74,178,684	cg16105620	74,178,684
23	423	11	74,178,586	74,178,784	cg16105620	74,178,684
23	423	11	74,178,586	74,178,784	cg04574090	74,178,749
23	423	11	74,178,586	74,178,784	cg02595219	74,178,761
23	424	11	74,178,668	74,178,824	cg16105620	74,178,684
23	424	11	74,178,668	74,178,824	cg04574090	74,178,749
23	424	11	74,178,668	74,178,824	cg02595219	74,178,761
23	424	11	74,178,668	74,178,824	cg11775521	74,178,795
23	424	11	74,178,668	74,178,824	cg18838431	74,178,800
23	425	11	74,178,763	74,178,951	cg11775521	74,178,795
23	425	11	74,178,763	74,178,951	cg18838431	74,178,800
23	425	11	74,178,763	74,178,951	cg16839921	74,178,883
24	426	12	108,732,484	108,732,670	NA	NA

264	284	11	128,813,366	128,813,528	cg23811942	128,813,470
265	285	12	7,456,893	7,457,048	cg14285764	7,456,988
266	286	12	22,778,393	22,778,559	cg24012034	22,778,485
267	287	12	46,320,829	46,321,020	cg01311909	46,320,890
267	287	12	46,320,829	46,321,020	cg05346476	46,320,944
268	288	12	50,473,406	50,473,528	cg18942451	50,473,456
269	289	12	52,638,521	52,638,644	cg07022048	52,638,592
270	290	12	58,013,570	58,013,696	cg14494721	58,013,636
270	290	12	58,013,570	58,013,696	cg04963424	58,013,645
270	290	12	58,013,570	58,013,696	cg16577509	58,013,651
270	290	12	58,013,570	58,013,696	cg08820231	58,013,687
271	291	12	64,915,907	64,916,095	cg02265721	64,916,007
272	292	12	92,540,391	92,540,544	cg05819371	92,540,484
273	293	12	95,942,877	95,943,011	cg13879483	95,942,907
273	293	12	95,942,877	95,943,011	cg07783282	95,942,964
274	294	12	104,680,853	104,681,028	cg14290287	104,680,923
275	295	12	110,157,607	110,157,766	cg19879537	110,157,712
276	296	12	117,480,265	117,480,410	cg06768361	117,480,333
277	297	12	120,032,056	120,032,215	cg18348736	120,032,142
278	298	12	125,477,506	125,477,656	cg04984513	125,477,549
278	298	12	125,477,506	125,477,656	cg19378602	125,477,596
279	299	12	125,578,271	125,578,466	cg02591564	125,578,355
280	300	12	130,589,058	130,589,200	cg21898944	130,589,151
281	301	12	133,065,744	133,065,881	cg20214067	133,065,776
281	301	12	133,065,744	133,065,881	cg24846573	133,065,806
281	301	12	133,065,744	133,065,881	cg23681339	133,065,808
282	302	13	28,367,600	28,367,779	cg14367229	28,367,741
282	303	13	28,367,710	28,367,882	cg14367229	28,367,741
283	304	13	34,392,670	34,392,831	cg24492140	34,392,781
284	305	13	36,044,791	36,044,954	cg00944193	36,044,860
284	305	13	36,044,791	36,044,954	cg02340053	36,044,886
285	306	13	69,557,883	69,558,035	cg18586262	69,557,960
286	307	13	72,245,888	72,246,061	cg12776754	72,246,001
287	308	13	100,548,380	100,548,578	cg01297756	100,548,415
287	308	13	100,548,380	100,548,578	cg13917662	100,548,513
288	309	13	103,052,700	103,052,874	cg22583065	103,052,828
289	310	13	110,433,947	110,434,101	cg01569664	110,434,016
290	311	13	111,000,203	111,000,357	cg03469869	111,000,343
290	312	13	111,000,308	111,000,467	cg03469869	111,000,343
291	313	13	112,190,967	112,191,119	NA	NA
291	314	13	112,191,105	112,191,286	cg27285197	112,191,122
291	314	13	112,191,105	112,191,286	cg14952266	112,191,215
291	314	13	112,191,105	112,191,286	cg11696108	112,191,272
292	315	13	114,064,948	114,065,086	cg19088635	114,065,008
293	316	14	20,482,844	20,483,027	cg18476566	20,482,899
294	317	14	50,886,349	50,886,509	cg09841898	50,886,416
295	318	14	60,040,621	60,040,806	cg21074911	60,040,732
296	319	14	60,629,120	60,629,291	cg06898279	60,629,192
297	320	14	61,120,151	61,120,307	cg15534086	61,120,234
298	321	14	62,228,539	62,228,698	cg07544451	62,228,636
298	321	14	62,228,539	62,228,698	cg12123983	62,228,668
299	322	14	64,010,701	64,010,885	cg19965063	64,010,753
299	322	14	64,010,701	64,010,885	cg04113056	64,010,798
299	322	14	64,010,701	64,010,885	cg21073637	64,010,844
300	323	14	64,562,654	64,562,792	cg13037437	64,562,705
301	324	14	68,444,730	68,444,873	cg04179571	68,444,789
302	325	14	71,632,555	71,632,732	cg15910301	71,632,612
303	326	14	73,270,862	73,271,028	cg09196248	73,270,968
304	327	14	92,418,055	92,418,197	cg08312152	92,418,138
305	328	14	95,477,492	95,477,596	cg23878730	95,477,582
305	329	14	95,477,545	95,477,715	cg23878730	95,477,582
306	330	14	100,798,723	100,798,891	cg04415736	100,798,844
307	331	14	101,990,011	101,990,168	cg06821183	101,990,064
308	332	14	105,119,492	105,119,612	cg01649538	105,119,562
309	333	15	25,486,270	25,486,429	cg01010923	25,486,368
310	334	15	39,875,655	39,875,837	cg01637789	39,875,777
311	335	15	42,210,034	42,210,178	cg07202353	42,210,119
312	336	15	43,785,445	43,785,623	cg12309516	43,785,537
313	337	15	44,116,699	44,116,866	cg07002382	44,116,782
313	337	15	44,116,699	44,116,866	cg03788666	44,116,829
314	338	15	45,004,520	45,004,644	cg08188779	45,004,577
315	339	15	50,613,650	50,613,775	cg26390924	50,613,702
316	340	15	62,533,382	62,533,565	cg02175578	62,533,502
317	341	15	63,449,695	63,449,847	cg02713581	63,449,717
317	341	15	63,449,695	63,449,847	cg19279045	63,449,725
317	341	15	63,449,695	63,449,847	cg02759888	63,449,753
317	341	15	63,449,695	63,449,847	cg21271767	63,449,808
317	341	15	63,449,695	63,449,847	cg09431919	63,449,821
317	341	15	63,449,695	63,449,847	cg10545276	63,449,841
317	342	15	63,449,799	63,449,913	cg21271767	63,449,808
317	342	15	63,449,799	63,449,913	cg09431919	63,449,821
317	342	15	63,449,799	63,449,913	cg10545276	63,449,841
317	342	15	63,449,799	63,449,913	cg23837188	63,449,871
317	342	15	63,449,799	63,449,913	cg13950367	63,449,913
318	343	15	63,784,983	63,785,132	cg26971423	63,785,031

24	427	12	108,732,580	108,732,766	NA	NA
24	428	12	108,732,681	108,732,822	NA	NA
24	429	12	108,732,748	108,732,907	NA	NA
24	430	12	108,732,802	108,732,987	NA	NA
24	431	12	108,732,920	108,733,102	cg06933965	108,732,994
24	432	12	108,733,034	108,733,163	NA	NA
24	433	12	108,733,119	108,733,249	NA	NA
24	434	12	108,733,205	108,733,369	cg25832824	108,733,253
24	434	12	108,733,205	108,733,369	cg03612522	108,733,261
24	434	12	108,733,205	108,733,369	cg12142354	108,733,275
24	434	12	108,733,205	108,733,369	cg15448445	108,733,308
24	434	12	108,733,205	108,733,369	cg08110272	108,733,321
24	435	12	108,733,277	108,733,476	cg15448445	108,733,308
24	435	12	108,733,277	108,733,476	cg08110272	108,733,321
24	435	12	108,733,277	108,733,476	cg03408433	108,733,370
24	436	12	108,733,421	108,733,602	NA	NA
24	437	12	108,733,544	108,733,666	NA	NA
24	438	12	108,733,581	108,733,714	NA	NA
24	439	12	108,733,667	108,733,761	NA	NA
24	440	12	108,733,693	108,733,859	cg00298951	108,733,791
24	441	12	108,733,737	108,733,902	cg00298951	108,733,791
24	442	12	108,733,838	108,733,981	NA	NA
24	443	12	108,733,918	108,734,095	NA	NA
24	444	12	108,734,009	108,734,183	NA	NA
25	445	15	90,645,770	90,645,959	cg02609028	90,645,872
25	445	15	90,645,770	90,645,959	cg19394101	90,645,875
25	445	15	90,645,770	90,645,959	cg09156067	90,645,907
25	446	15	90,645,883	90,646,076	cg09156067	90,645,907
25	446	15	90,645,883	90,646,076	cg24995652	90,645,967
25	447	15	90,646,003	90,646,197	cg11963595	90,646,093
25	448	15	90,646,108	90,646,262	NA	NA
25	449	15	90,646,173	90,646,324	NA	NA
25	450	15	90,646,373	90,646,557	NA	NA
25	451	15	90,646,456	90,646,624	NA	NA
25	452	15	90,646,538	90,646,731	cg25244102	90,646,631
25	453	15	90,646,656	90,646,855	NA	NA
25	454	15	90,646,768	90,646,919	NA	NA
25	455	15	90,646,857	90,647,056	NA	NA
25	456	15	90,646,946	90,647,145	NA	NA
25	457	15	90,647,046	90,647,233	NA	NA
25	458	15	90,647,169	90,647,354	NA	NA
25	459	15	90,647,257	90,647,423	NA	NA
25	460	15	90,647,360	90,647,488	NA	NA
26	461	20	17,295,183	17,295,327	cg20371142	17,295,221
26	462	20	17,295,237	17,295,420	NA	NA
26	463	20	17,295,286	17,295,473	NA	NA
26	464	20	17,295,397	17,295,585	NA	NA
26	465	20	17,295,492	17,295,686	NA	NA
26	466	20	17,295,589	17,295,788	NA	NA
26	467	20	17,295,747	17,295,868	cg17135722	17,295,815
26	468	20	17,295,775	17,295,943	cg17135722	17,295,815
26	469	20	17,295,907	17,296,057	cg23756474	17,296,030
26	470	20	17,295,926	17,296,086	cg23756474	17,296,030
26	471	20	17,296,034	17,296,161	cg16134686	17,296,161
26	472	20	17,296,090	17,296,260	cg16134686	17,296,161
26	473	20	17,296,143	17,296,303	cg16134686	17,296,161
26	474	20	17,296,247	17,296,341	cg09165842	17,296,317
26	475	20	17,296,268	17,296,382	cg09165842	17,296,317
26	476	20	17,296,319	17,296,469	cg20157281	17,296,450
26	477	20	17,296,364	17,296,558	cg20157281	17,296,450
26	478	20	17,296,488	17,296,620	cg13698778	17,296,597
26	479	20	17,296,535	17,296,716	cg13698778	17,296,597
26	480	20	17,296,598	17,296,791	NA	NA
26	481	20	17,296,688	17,296,878	NA	NA
27	482	22	42,827,548	42,827,746	NA	NA
27	483	22	42,827,667	42,827,859	NA	NA
27	484	22	42,827,813	42,827,976	NA	NA
27	485	22	42,827,933	42,828,055	NA	NA
27	486	22	42,827,956	42,828,150	cg17568996	42,828,125
27	487	22	42,828,043	42,828,232	cg17568996	42,828,125
27	488	22	42,828,138	42,828,296	NA	NA
27	489	22	42,828,209	42,828,382	NA	NA
27	490	22	42,828,341	42,828,445	cg03365751	42,828,386
27	490	22	42,828,341	42,828,445	cg27098470	42,828,411
27	490	22	42,828,341	42,828,445	cg07264666	42,828,415
27	490	22	42,828,341	42,828,445	cg09335713	42,828,418
27	491	22	42,828,363	42,828,515	cg03365751	42,828,386
27	491	22	42,828,363	42,828,515	cg27098470	42,828,411
27	491	22	42,828,363	42,828,515	cg07264666	42,828,415
27	491	22	42,828,363	42,828,515	cg09335713	42,828,418
27	491	22	42,828,363	42,828,515	cg01383955	42,828,447
27	492	22	42,828,426	42,828,604	cg01383955	42,828,447
27	492	22	42,828,426	42,828,604	cg07044422	42,828,516
27	493	22	42,828,492	42,828,681	cg07044422	42,828,516
27	494	22	42,828,633	42,828,775	NA	NA

319	344	15	65,107,995	65,108,160	cg04842787	65,108,040
320	345	15	74,889,227	74,889,396	cg07708777	74,889,327
321	346	15	85,923,476	85,923,645	cg06742159	85,923,547
321	346	15	85,923,476	85,923,645	cg21325941	85,923,618
322	347	15	86,233,182	86,233,337	cg05341539	86,233,214
322	347	15	86,233,182	86,233,337	cg25947619	86,233,220
322	347	15	86,233,182	86,233,337	cg25510609	86,233,236
323	348	15	88,799,404	88,799,523	cg11525479	88,799,523
324	349	15	92,612,781	92,612,914	cg02107844	92,612,836
325	350	15	93,198,958	93,199,127	cg02705800	93,199,018
325	350	15	93,198,958	93,199,127	cg25864024	93,199,037
325	350	15	93,198,958	93,199,127	cg15289316	93,199,049
326	351	15	100,249,090	100,249,237	cg07382129	100,249,186
327	352	16	610,838	610,990	cg08950915	610,895
328	353	16	703,337	703,508	cg04343385	703,464
329	354	16	1,017,755	1,017,866	cg07197480	1,017,808
330	355	16	1,243,449	1,243,608	cg01128460	1,243,544
331	356	16	1,336,592	1,336,780	cg16748643	1,336,696
332	357	16	2,180,076	2,180,250	cg08114547	2,180,141
333	358	16	3,921,140	3,921,335	cg01591591	3,921,282
334	359	16	4,733,052	4,733,247	cg16520815	4,733,181
335	360	16	10,965,159	10,965,280	cg10466548	10,965,217
336	361	16	13,135,457	13,135,616	cg07631533	13,135,565
337	362	16	15,489,109	15,489,240	cg06405517	15,489,190
337	362	16	15,489,109	15,489,240	cg09294492	15,489,203
338	363	16	19,991,626	19,991,747	cg26672573	19,991,691
339	364	16	20,814,643	20,814,795	cg06579338	20,814,739
340	365	16	30,034,697	30,034,851	cg05034471	30,034,801
341	366	16	30,457,107	30,457,236	cg07842593	30,457,166
342	367	16	30,686,472	30,686,660	cg00336022	30,686,562
343	368	16	31,408,811	31,408,929	cg26767387	31,408,867
343	368	16	31,408,811	31,408,929	cg10174031	31,408,898
343	368	16	31,408,811	31,408,929	cg07228987	31,408,900
343	369	16	31,408,868	31,409,055	cg10174031	31,408,898
343	369	16	31,408,868	31,409,055	cg07228987	31,408,900
344	370	16	54,965,014	54,965,122	cg02056682	54,965,084
344	370	16	54,965,014	54,965,122	cg05870072	54,965,087
344	370	16	54,965,014	54,965,122	cg05362516	54,965,089
344	370	16	54,965,014	54,965,122	cg06191898	54,965,100
345	371	16	57,278,524	57,278,700	cg26527793	57,278,643
346	372	16	64,016,958	64,017,150	cg06074252	64,017,087
347	373	16	65,098,771	65,098,870	cg06125461	65,098,852
347	374	16	65,098,810	65,099,000	cg06125461	65,098,852
348	375	16	70,380,410	70,380,584	cg10356341	70,380,474
348	375	16	70,380,410	70,380,584	cg00607725	70,380,558
349	376	16	75,271,076	75,271,225	cg01215963	75,271,147
349	376	16	75,271,076	75,271,225	cg09121928	75,271,179
350	377	16	85,676,314	85,676,457	cg26648465	85,676,412
351	378	16	86,872,461	86,872,623	cg10324585	86,872,570
352	379	16	87,099,993	87,100,150	cg09331651	87,100,066
353	380	16	87,796,211	87,796,368	cg07208891	87,796,307
354	381	16	88,019,995	88,020,131	cg16553026	88,020,079
355	382	16	88,177,667	88,177,804	cg26894673	88,177,780
355	383	16	88,177,755	88,177,952	cg26894673	88,177,780
356	384	16	88,704,962	88,705,099	cg27132152	88,705,016
357	385	17	1,633,642	1,633,794	cg21824343	1,633,714
357	385	17	1,633,642	1,633,794	cg02736280	1,633,745
358	386	17	4,692,229	4,692,366	cg03704673	4,692,287
359	387	17	4,891,477	4,891,587	cg14928057	4,891,542
360	388	17	7,037,025	7,037,151	cg08154612	7,037,098
361	389	17	7,165,204	7,165,351	cg15298719	7,165,255
362	390	17	7,405,819	7,405,930	cg07615572	7,405,889
362	391	17	7,405,867	7,406,026	cg07615572	7,405,889
362	391	17	7,405,867	7,406,026	cg17016524	7,405,958
363	392	17	8,021,953	8,022,114	cg12210770	8,022,010
364	393	17	8,906,357	8,906,547	cg04743961	8,906,382
364	393	17	8,906,357	8,906,547	cg21721093	8,906,386
364	394	17	8,906,528	8,906,699	cg07848706	8,906,601
365	395	17	21,188,360	21,188,519	cg16597437	21,188,442
365	395	17	21,188,360	21,188,519	cg05679521	21,188,468
366	396	17	21,220,715	21,220,874	cg16320699	21,220,807
367	397	17	27,966,666	27,966,798	cg16033151	27,966,740
368	398	17	37,828,223	37,828,374	cg19758448	37,828,296
369	399	17	40,175,765	40,175,961	cg26535158	40,175,841
370	400	17	40,464,263	40,464,395	cg05548912	40,464,318
371	401	17	41,312,403	41,312,533	cg02173360	41,312,467
372	402	17	42,392,505	42,392,652	cg03061122	42,392,559
373	403	17	43,044,878	43,045,058	cg04819499	43,045,014
374	404	17	43,339,411	43,339,569	cg17222164	43,339,476
374	404	17	43,339,411	43,339,569	cg03048083	43,339,497
374	404	17	43,339,411	43,339,569	cg01627847	43,339,512
374	404	17	43,339,411	43,339,569	cg08124910	43,339,515
375	405	17	54,828,905	54,829,064	cg14266156	54,829,018
376	406	17	56,084,407	56,084,548	cg24269276	56,084,441
376	406	17	56,084,407	56,084,548	cg04498971	56,084,468

27	495	22	42,828,673	42,828,847	NA	NA
27	496	22	42,828,757	42,828,944	NA	NA
27	497	22	42,828,869	42,829,060	cg07464578	42,828,946
27	498	22	42,828,925	42,829,117	cg07464578	42,828,946
27	499	22	42,829,044	42,829,182	NA	NA
27	500	22	42,829,135	42,829,242	NA	NA

376	406	17	56,084,407	56,084,548	cg14553224	56,084,507
377	407	17	75,368,850	75,369,019	cg06848185	75,368,902
377	408	17	75,368,995	75,369,168	cg19554255	75,369,051
377	408	17	75,368,995	75,369,168	cg16779463	75,369,055
377	408	17	75,368,995	75,369,168	cg17300544	75,369,091
377	409	17	75,369,102	75,369,272	cg03804136	75,369,219
377	409	17	75,369,102	75,369,272	cg15044248	75,369,224
377	409	17	75,369,102	75,369,272	cg02884239	75,369,228
378	410	17	76,220,836	76,220,997	cg19272238	76,220,898
378	410	17	76,220,836	76,220,997	cg07366188	76,220,929
378	410	17	76,220,836	76,220,997	cg10070788	76,220,955
379	411	17	76,348,748	76,348,914	cg17845279	76,348,823
379	411	17	76,348,748	76,348,914	cg21640526	76,348,849
380	412	17	76,921,811	76,921,999	cg06641285	76,921,829
380	412	17	76,921,811	76,921,999	cg05306745	76,921,845
380	412	17	76,921,811	76,921,999	cg15981475	76,921,948
381	413	17	76,932,217	76,932,388	cg14779622	76,932,315
381	413	17	76,932,217	76,932,388	cg17552357	76,932,332
382	414	17	78,155,158	78,155,313	cg13855862	78,155,264
383	415	17	79,415,561	79,415,759	cg01810575	79,415,682
384	416	17	79,971,390	79,971,506	cg06105699	79,971,436
385	417	17	80,709,099	80,709,345	cg00960700	80,709,150
386	418	18	12,911,072	12,911,244	cg25356164	12,911,138
387	419	18	34,327,403	34,327,561	cg21367586	34,327,498
388	420	18	74,499,448	74,499,560	cg25691825	74,499,511
389	421	18	74,848,763	74,848,902	cg12635694	74,848,845
390	422	18	77,793,100	77,793,237	cg04727522	77,793,182
391	423	19	2,131,225	2,131,338	cg05718393	2,131,293
391	423	19	2,131,225	2,131,338	cg23103406	2,131,296
392	424	19	2,643,016	2,643,134	cg15425921	2,643,085
393	425	19	6,495,792	6,495,950	cg06548442	6,495,872
393	425	19	6,495,792	6,495,950	cg11915671	6,495,897
394	426	19	6,746,129	6,746,248	cg00513012	6,746,184
394	426	19	6,746,129	6,746,248	cg19736654	6,746,221
395	427	19	8,214,628	8,214,731	cg17909996	8,214,685
396	428	19	8,273,644	8,273,781	cg24413781	8,273,693
397	429	19	10,450,412	10,450,532	cg18054725	10,450,472
398	430	19	31,843,302	31,843,447	cg22079008	31,843,360
398	430	19	31,843,302	31,843,447	cg09253179	31,843,396
399	431	19	35,249,109	35,249,273	cg00019275	35,249,217
400	432	19	37,019,197	37,019,372	cg23949233	37,019,255
400	432	19	37,019,197	37,019,372	cg16885608	37,019,262
400	432	19	37,019,197	37,019,372	cg18105335	37,019,307
400	432	19	37,019,197	37,019,372	cg18882201	37,019,327
400	432	19	37,019,197	37,019,372	cg19990402	37,019,372
401	433	19	37,861,428	37,861,557	cg06610641	37,861,546
401	434	19	37,861,533	37,861,645	cg06610641	37,861,546
402	435	19	38,085,577	38,085,746	cg27321876	38,085,706
402	435	19	38,085,577	38,085,746	cg16708785	38,085,709
403	436	19	45,654,162	45,654,321	cg27052073	45,654,213
404	437	19	45,901,506	45,901,703	cg08512167	45,901,567
404	437	19	45,901,506	45,901,703	cg13645732	45,901,649
405	438	19	46,180,196	46,180,355	cg18642271	46,180,314
405	439	19	46,180,306	46,180,440	cg18642271	46,180,314
405	439	19	46,180,306	46,180,440	cg17144108	46,180,412
406	440	19	46,318,696	46,318,892	cg27118825	46,318,748
406	440	19	46,318,696	46,318,892	cg04379348	46,318,762
407	441	19	47,923,301	47,923,461	cg07478208	47,923,331
407	441	19	47,923,301	47,923,461	cg17910813	47,923,387
408	442	19	48,918,066	48,918,187	cg15779837	48,918,116
409	443	19	54,377,144	54,377,342	cg18054632	54,377,302
409	444	19	54,377,302	54,377,463	cg18691133	54,377,440
410	445	19	58,514,764	58,514,943	cg05377415	58,514,816
410	445	19	58,514,764	58,514,943	cg27295481	58,514,839
411	446	20	271,313	271,469	cg11991627	271,345
411	446	20	271,313	271,469	cg23989297	271,347
411	446	20	271,313	271,469	cg17441724	271,417
411	446	20	271,313	271,469	cg25232745	271,422
411	446	20	271,313	271,469	cg06440615	271,425
411	446	20	271,313	271,469	cg25031040	271,432
412	447	20	304,008	304,179	cg01822050	304,156
413	448	20	2,508,885	2,509,043	cg07545427	2,508,981
414	449	20	20,349,139	20,349,565	cg13326227	20,349,168
414	449	20	20,349,139	20,349,565	cg00648301	20,349,241
415	450	20	24,449,631	24,449,790	cg05347927	24,449,668
415	450	20	24,449,631	24,449,790	cg22479299	24,449,704
415	450	20	24,449,631	24,449,790	cg02997755	24,449,734
415	450	20	24,449,631	24,449,790	cg22452236	24,449,760
416	451	20	24,450,260	24,450,404	cg09623400	24,450,353
416	451	20	24,450,260	24,450,404	cg19654195	24,450,361
417	452	20	32,378,120	32,378,279	cg01554089	32,378,171
418	453	20	34,099,423	34,099,569	cg04070427	34,099,511
419	454	20	36,013,363	36,013,478	cg23635789	36,013,428
420	455	20	36,153,843	36,153,992	cg03226872	36,153,865
420	455	20	36,153,843	36,153,992	cg17271585	36,153,947

421	456	20	42,142,967	42,143,118	cg22601123	42,142,995
421	456	20	42,142,967	42,143,118	cg22330467	42,143,015
421	456	20	42,142,967	42,143,118	cg14306330	42,143,045
421	456	20	42,142,967	42,143,118	cg01071811	42,143,080
421	456	20	42,142,967	42,143,118	cg02611863	42,143,096
422	457	20	43,161,193	43,161,315	cg00726046	43,161,260
423	458	20	44,782,043	44,782,220	cg11206526	44,782,126
424	459	20	48,728,592	48,728,758	cg13046524	48,728,642
425	460	20	50,158,137	50,158,289	cg02561912	50,158,202
426	461	20	56,881,887	56,882,037	cg21532801	56,881,949
427	462	20	58,586,763	58,586,907	cg02423105	58,586,819
428	463	20	60,701,221	60,701,380	cg06096175	60,701,350
428	464	20	60,701,310	60,701,504	cg06096175	60,701,350
429	465	20	61,803,852	61,804,017	cg13590979	61,803,906
430	466	20	62,124,844	62,125,003	cg14636268	62,124,947
431	467	21	30,365,345	30,365,502	cg01446372	30,365,398
431	467	21	30,365,345	30,365,502	cg23864210	30,365,465
432	468	21	34,398,760	34,398,899	cg14843922	34,398,849
433	469	21	36,412,349	36,412,507	cg16071713	36,412,456
434	470	21	43,098,798	43,098,952	cg20206277	43,098,901
435	471	21	47,784,037	47,784,223	cg05931989	47,784,119
436	472	22	19,748,761	19,748,948	cg02583938	19,748,777
436	472	22	19,748,761	19,748,948	cg16332936	19,748,910
436	472	22	19,748,761	19,748,948	cg16113681	19,748,925
436	473	22	19,748,917	19,749,020	cg16113681	19,748,925
437	474	22	21,333,564	21,333,718	cg06985578	21,333,706
438	475	22	30,723,129	30,723,265	cg10574851	30,723,212
438	475	22	30,723,129	30,723,265	cg24944736	30,723,217
439	476	22	39,414,416	39,414,543	cg27451450	39,414,471
440	477	22	41,183,411	41,183,603	cg03888645	41,183,474
441	478	22	42,949,775	42,949,924	cg04416895	42,949,810
441	478	22	42,949,775	42,949,924	cg08157579	42,949,829
442	479	22	42,976,088	42,976,208	cg08094784	42,976,149
443	480	22	46,831,936	46,832,056	cg17551295	46,831,988
444	481	22	50,435,357	50,435,476	cg18730511	50,435,413
445	482	X	11,683,507	11,683,626	cg21880156	11,683,548
445	482	X	11,683,507	11,683,626	cg18036967	11,683,625
445	483	X	11,683,601	11,683,783	cg18036967	11,683,625
446	484	X	23,017,880	23,018,046	cg21908878	23,017,887
446	484	X	23,017,880	23,018,046	cg14020146	23,017,980
446	484	X	23,017,880	23,018,046	cg26009591	23,017,991
446	484	X	23,017,880	23,018,046	cg08264725	23,018,003
446	484	X	23,017,880	23,018,046	cg27124742	23,018,006
446	484	X	23,017,880	23,018,046	cg22052586	23,018,036
447	485	X	30,904,301	30,904,451	cg05204193	30,904,380
448	486	X	66,765,192	66,765,368	cg03096488	66,765,289
449	487	X	69,355,989	69,356,149	cg00713642	69,356,087
450	488	X	69,509,620	69,509,767	cg18030003	69,509,686
450	488	X	69,509,620	69,509,767	cg17206029	69,509,694
450	488	X	69,509,620	69,509,767	cg00213957	69,509,697
450	488	X	69,509,620	69,509,767	cg13622893	69,509,727
451	489	X	69,673,743	69,673,865	cg01448525	69,673,805
452	490	X	70,316,329	70,316,487	cg18288715	70,316,444
453	491	X	73,513,315	73,513,500	cg10575170	73,513,412
453	491	X	73,513,315	73,513,500	cg07823797	73,513,418
453	491	X	73,513,315	73,513,500	cg19487868	73,513,453
453	491	X	73,513,315	73,513,500	cg04667267	73,513,464
454	492	X	75,249,487	75,249,685	cg08103876	75,249,519
454	492	X	75,249,487	75,249,685	cg03236853	75,249,539
455	493	X	117,629,107	117,629,286	cg27062326	117,629,163
456	494	X	118,750,201	118,750,354	cg17385225	118,750,273
457	495	X	135,333,537	135,333,704	cg07446674	135,333,561
457	495	X	135,333,537	135,333,704	cg27047283	135,333,567
457	495	X	135,333,537	135,333,704	cg24347720	135,333,661
457	495	X	135,333,537	135,333,704	cg13304035	135,333,674
457	495	X	135,333,537	135,333,704	cg13243544	135,333,698
458	496	X	136,115,950	136,116,119	cg14463432	136,115,991
459	497	X	138,287,387	138,287,578	cg06981242	138,287,529
459	497	X	138,287,387	138,287,578	cg08349204	138,287,578
460	498	X	139,587,254	139,587,372	cg23862713	139,587,270
460	498	X	139,587,254	139,587,372	cg26369916	139,587,279
460	498	X	139,587,254	139,587,372	cg07929406	139,587,304
460	498	X	139,587,254	139,587,372	cg03202526	139,587,311
460	498	X	139,587,254	139,587,372	cg11638117	139,587,335
460	498	X	139,587,254	139,587,372	cg11409998	139,587,372
460	499	X	139,587,337	139,587,500	cg11409998	139,587,372
460	499	X	139,587,337	139,587,500	cg24432916	139,587,479
461	500	X	144,903,592	144,903,733	cg24880787	144,903,661
461	500	X	144,903,592	144,903,733	cg19332075	144,903,691
462	501	X	151,143,173	151,143,343	cg18748981	151,143,213
462	501	X	151,143,173	151,143,343	cg25528646	151,143,302

Table S2. Summary sequencing and methylation statistics for the (A) SC panel and (B) BM panel. Summary statistics regarding trimming and mapping of sequencing reads, as well as methylation levels are reported. The data sets were analyzed as described in the Methods section.

(A) SC Panel

	BL 100	BL 250	BL 1000	BL 1500	BL 10-A	BL 50-A	BL 100-A	BL 250-A	SCL 100	SCL 250	SCL 1000	SCL 1500	SCL 10-A	SCL 50-A	SCL 100-A	SCL 250-A
**Raw sequencing read quality filtering (fastq-mcf)**																
Total number of sequencing reads	305,590	253,255	273,603	215,406	190,386	448,017	466,972	535,264	324,934	404,680	234,005	243,583	381,979	372,326	425,036	456,764
Number of discarded low-quality reads	20,128	15,497	13,564	7,548	33,127	64,864	64,978	49,528	23,697	30,245	13,826	11,846	68,043	46,823	68,224	14,009
**Final alignment report (Bismark)**																
Total number of sequencing reads analyzed	285,462	237,758	260,039	207,858	157,259	383,153	401,994	485,736	301,237	374,435	220,179	231,737	313,936	325,503	356,812	442,755
Sequence pairs with a unique best hit	50,950	52,449	88,158	77,987	5,537	50,661	47,885	72,498	30,035	59,954	61,605	65,520	14,519	11,948	36,301	52,171
CT/GA/CT (converted top strand)	25,841	26,950	45,388	40,886	2,990	24,954	24,425	38,147	16,140	29,269	30,902	33,186	8,445	5,933	18,655	26,929
GA/CT/CT (compl. to converted top strand)	812	571	1,146	586	238	2,212	2,048	3,018	360	897	656	517	603	981	2,205	1,413
GA/CT/GA (compl. to converted bottom strand)	23,766	24,498	41,158	36,164	1,422	22,489	20,505	30,283	13,101	29,143	29,572	31,403	4,048	3,733	14,339	23,367
CT/GA/GA (converted bottom strand)	531	430	466	351	887	1,006	907	1,050	434	645	475	414	1,423	1,301	1,102	462
Mapping efficiency	17.80%	22.10%	33.90%	37.50%	3.50%	13.20%	11.90%	14.90%	10.00%	16.00%	28.00%	28.30%	4.60%	3.70%	10.20%	11.80%
Sequence pairs with no alignments under any condition	230,296	182,088	169,151	128,073	147,169	323,119	345,266	403,760	265,673	308,019	155,755	163,526	289,682	305,764	311,638	387,963
Sequence pairs that did not map uniquely	4,216	3,221	2,730	1,798	4,553	9,373	8,843	9,478	5,529	6,462	2,819	2,691	9,735	7,791	8,873	2,621
Sequence pairs that were discarded, because genomic sequence could not be extracted	0	0	0	0	0	0	0	0	0	0	0	0	0	0	0	0
**Targeted sequencing statistics (TEQC)**																
Number of sequencing reads used	101,900	104,898	176,316	155,974	11,074	101,322	95,770	144,996	60,070	119,908	123,210	131,040	29,038	23,896	72,602	104,342
Fraction of aligned reads that overlap with any target region	0.947	0.958	0.961	0.974	0.660	0.871	0.869	0.876	0.930	0.943	0.962	0.968	0.743	0.642	0.819	0.918
**Final cytosine methylation report (Bismark)**																
Total number of C's analyzed	1,755,662	1,809,211	3,142,878	2,791,484	88,718	1,486,819	1,440,186	2,304,494	938,487	1,979,953	2,138,844	2,322,030	283,795	245,177	968,769	1,726,513
Total methylated C's in CpG context	60,894	64,307	112,329	99,370	2,006	50,213	54,278	80,600	96,638	208,548	239,126	266,576	11,008	9,712	61,519	138,007
Total methylated C's in CHG context	4,521	4,703	7,691	6,379	352	2,870	4,833	5,066	2,686	5,171	4,082	4,707	747	811	1,760	4,370
Total methylated C's in CHH context	23,504	21,285	25,009	19,679	19,638	41,267	36,018	42,676	20,708	29,434	16,916	18,296	40,594	37,655	34,924	22,195
Total C to T conversions in CpG context	264,855	285,653	472,920	457,113	4,648	159,028	157,429	268,765	69,294	142,078	158,502	174,856	17,444	13,312	55,533	133,988
Total C to T conversions in CHG context	515,501	540,786	937,339	849,569	16,818	424,062	410,229	659,168	280,052	587,904	638,813	701,058	65,738	55,388	270,147	510,946
Total C to T conversions in CHH context	886,387	892,477	1,587,590	1,359,374	45,256	809,379	777,399	1,248,219	469,109	1,006,818	1,081,405	1,156,537	148,264	128,299	544,886	917,007
C methylated in CpG context	18.70%	18.40%	19.20%	17.90%	30.10%	24.00%	25.60%	23.10%	58.20%	59.50%	60.10%	60.40%	38.70%	42.20%	52.60%	50.70%
C methylated in CHG context	0.90%	0.90%	0.80%	0.70%	2.10%	0.70%	1.20%	0.80%	0.90%	0.90%	0.60%	0.70%	1.10%	1.40%	0.60%	0.80%
C methylated in CHH context	2.60%	2.30%	1.60%	1.40%	30.30%	4.90%	4.40%	3.30%	4.20%	2.80%	1.50%	1.60%	21.50%	22.70%	6.00%	2.40%

	FFPE 100	FFPE 250	FFPE 1000	FFPE 1500	FFPE 10-A	FFPE 50-A	FFPE 100-A	FFPE 250-A
**Raw sequencing read quality filtering (fastq-mcf)**								
Total number of sequencing reads	197,172	190,892	97,956	54,414	320,858	274,304	452,992	296,896
Number of discarded low-quality reads	18,754	14,777	8,778	5,222	50,972	51,514	49,175	29,535
**Final alignment report (Bismark)**								
Total number of sequencing reads analyzed	178,418	176,115	89,178	49,192	269,886	222,790	403,817	267,361
Sequence pairs with a unique best hit	8,607	8,394	4,377	3,912	9,570	8,974	9,914	6,443
CT/GA/CT (converted top strand)	5,156	4,800	2,419	2,039	5,752	5,716	5,703	3,363

GA/CT/CT (compl. to converted top strand)	180	220	100	115	267	211	519	642
GA/CT/GA (compl. to converted bottom strand)	2,856	3,046	1,637	1,615	2,594	2,305	2,702	1,706
CT/GA/GA (converted bottom strand)	415	328	221	143	957	742	990	732
Mapping efficiency	4.80%	4.80%	4.90%	8.00%	3.50%	4.00%	2.50%	2.40%
Sequence pairs with no alignments under any condition	164,887	163,706	82,577	44,172	251,252	205,615	385,703	255,696
Sequence pairs that did not map uniquely	4,924	4,015	2,224	1,108	9,064	8,201	8,200	5,222
Sequence pairs that were discarded, because genomic sequence could not be extracted	0	0	0	0	0	0	0	0
**Targeted sequencing statistics (TEQC)**								
Number of sequencing reads used	17,214	16,788	8,754	7,824	19,140	17,948	19,828	12,886
Fraction of aligned reads that overlap with any target region	0.837	0.841	0.814	0.845	0.713	0.748	0.666	0.598
**Final cytosine methylation report (Bismark)**								
Total number of C's analyzed	221,077	229,836	115,732	115,309	137,560	120,540	178,147	124,523
Total methylated C's in CpG context	8,041	8,729	4,522	4,097	2,786	2,777	8,295	6,532
Total methylated C's in CHG context	507	722	341	274	399	346	642	647
Total methylated C's in CHH context	14,591	11,803	7,604	4,325	31,410	24,993	34,373	27,797
Total C to T conversions in CpG context	21,305	23,207	13,108	13,443	4,359	3,785	7,168	5,151
Total C to T conversions in CHG context	57,517	64,522	31,638	32,685	27,563	24,942	36,690	23,436
Total C to T conversions in CHH context	119,116	120,853	58,519	60,485	71,043	63,697	90,979	60,960
C methylated in CpG context	27.40%	27.30%	25.60%	23.40%	39.00%	42.30%	53.60%	55.90%
C methylated in CHG context	0.90%	1.10%	1.10%	0.80%	1.40%	1.40%	1.70%	2.70%
C methylated in CHH context	10.90%	8.90%	11.50%	6.70%	30.70%	28.20%	27.40%	31.30%

(B) BM Panel

	BL 100	BL 250	BL 1000	BL 1500	BL 10-A	BL 50-A	BL 100-A	BL 250-A	SCL 100	SCL 250	SCL 1000	SCL 1500	SCL 10-A	SCL 50-A	SCL 100-A	SCL 250-A
**Raw sequencing read quality filtering (fastq-mcf)**																
Total number of sequencing reads	582,276	804,368	429,851	378,921	854,499	430,215	759,866	1,053,732	476,191	673,353	453,915	283,451	364,617	600,335	605,800	1,054,642
Number of discarded low-quality reads	22,617	30,032	11,855	7,929	69,394	34,255	52,774	59,765	24,612	29,870	14,475	7,983	37,056	58,337	52,120	76,018
**Final alignment report (Bismark)**																
Total number of sequencing reads analyzed	559,659	774,336	417,996	370,992	785,105	395,960	707,092	993,967	451,579	643,483	439,440	275,468	327,561	541,998	553,680	978,624
Sequence pairs with a unique best hit	65,766	101,257	121,647	134,812	24,239	13,951	57,757	142,385	30,992	83,346	116,942	90,278	15,944	26,081	46,530	112,189
CT/GA/CT (converted top strand)	31,448	48,727	59,434	65,413	5,595	4,170	23,418	64,652	13,517	38,180	54,973	43,087	4,977	8,732	18,851	50,189
GA/CT/CT (compl. to converted top strand)	1,867	2,762	1,582	1,478	2,965	1,339	2,482	3,563	1,408	2,474	1,614	987	997	1,752	1,936	3,581
GA/CT/GA (compl. to converted bottom strand)	27,997	43,923	57,794	65,587	8,469	5,066	25,116	64,919	12,284	37,522	57,082	44,245	5,952	9,316	19,908	48,365
CT/GA/GA (converted bottom strand)	4,454	5,845	2,837	2,334	7,210	3,376	6,741	9,251	3,783	5,170	3,273	1,959	4,018	6,281	5,835	10,054
Mapping efficiency	11.80%	13.10%	29.10%	36.30%	3.10%	3.50%	8.20%	14.30%	6.90%	13.00%	26.60%	32.80%	4.90%	4.80%	8.40%	11.50%
Sequence pairs with no alignments under any condition	480,871	655,652	288,720	229,817	733,782	368,840	630,936	828,445	408,373	543,673	313,012	180,031	301,577	498,888	491,209	841,144
Sequence pairs that did not map uniquely	13,022	17,427	7,629	6,363	27,084	13,169	18,399	23,137	12,214	16,464	9,486	5,159	10,040	17,029	15,941	25,291
Sequence pairs that were discarded, because genomic sequence could not be extracted	0	0	0	0	0	0	0	0	0	0	0	0	0	0	0	0
**Targeted sequencing statistics (TEQC)**																
Number of sequencing reads used	131,532	202,514	243,294	269,624	48,478	27,902	115,514	284,770	61,984	166,692	233,884	180,556	31,888	52,162	93,060	224,378
Fraction of aligned reads that overlap with any target region	0.730	0.760	0.888	0.912	0.115	0.180	0.590	0.750	0.546	0.742	0.873	0.899	0.301	0.286	0.583	0.687
**Final cytosine methylation report (Bismark)**																
Total number of C's analyzed	2,231,032	3,475,046	4,274,365	4,801,645	482,071	297,007	1,797,890	4,706,481	940,575	2,774,485	4,042,875	3,165,998	392,178	625,554	1,401,229	3,537,416
Total methylated C's in CpG context	136,827	211,382	248,228	272,026	8,619	8,548	107,597	280,668	68,985	225,261	331,199	264,391	15,469	26,771	101,189	252,659

Total methylated C's in CHG context	4,556	6,707	7,749	8,034	2,857	1,631	3,554	9,194	2,173	5,677	6,565	4,660	1,161	1,716	2,691	6,356
Total methylated C's in CHH context	88,335	123,030	59,833	48,119	224,813	107,940	156,072	189,735	96,218	123,836	71,269	39,661	112,347	172,607	142,900	220,610
Total C to T conversions in CpG context	243,117	375,397	493,633	576,172	10,774	10,426	157,013	441,440	63,987	212,005	333,917	265,317	20,322	27,843	89,924	237,358
Total C to T conversions in CHG context	593,709	922,917	1,168,209	1,320,134	38,430	37,076	445,782	1,250,708	222,187	727,832	1,106,767	871,985	61,944	105,902	339,741	900,274
Total C to T conversions in CHH context	1,164,488	1,835,613	2,296,713	2,577,160	196,578	131,386	927,872	2,534,736	487,025	1,479,874	2,193,158	1,719,984	180,935	290,715	724,784	1,920,159
C methylated in CpG context	36.00%	36.00%	33.50%	32.10%	44.40%	45.10%	40.70%	38.90%	51.90%	51.50%	49.80%	49.90%	43.20%	49.00%	52.90%	51.60%
C methylated in CHG context	0.80%	0.70%	0.70%	0.60%	6.90%	4.20%	0.80%	0.70%	1.00%	0.80%	0.60%	0.50%	1.80%	1.60%	0.80%	0.70%
C methylated in CHH context	7.10%	6.30%	2.50%	1.80%	53.40%	45.10%	14.40%	7.00%	16.50%	7.70%	3.10%	2.30%	38.30%	37.30%	16.50%	10.30%

Table S3. Primer sequences for the (A) universal PCR and (B) Illumina sequencing reaction. In the universal PCR, the reverse primers harbored a sample-specific 8-bp barcode. The column 'Barcode' provides the reverse complementary DNA sequences of these barcodes, which were used for demultiplexing in the subsequent Illumina sequencing reaction. A total of 96 barcodes (based on Kozarewa I &Turner DJ. Methods Mol Biol 2011; 733:279-98) are shown. For the universal PCR using the SC and BM panel, we used the primers M_rev_25–48 and M_rev_25–40, respectively. For the sequencing reaction on a MiSeq DNA sequencer [Illumina], we used custom Read1 and Read2 primers.

(A) Universal PCR

ID	Sequence	Barcode
U_fwd	5'-AATGATACGGCGACCACCGAGATCTACACTCTTTCCCTACACGACGCTCTTCCGATCTCTG	NA
U_rev_1	5'-CAAGCAGAAGACGGCATACGAGAT-ACAAGCTA-GTGACTGGAGTTCAGACGTGTGCTCTTCCGATCTGAC	TAGCTTGT
U_rev_2	5'-CAAGCAGAAGACGGCATACGAGAT-AAACATCG-GTGACTGGAGTTCAGACGTGTGCTCTTCCGATCTGAC	CGATGTTT
U_rev_3	5'-CAAGCAGAAGACGGCATACGAGAT-ACATTGGC-GTGACTGGAGTTCAGACGTGTGCTCTTCCGATCTGAC	GCCAATGT
U_rev_4	5'-CAAGCAGAAGACGGCATACGAGAT-ACCACTGT-GTGACTGGAGTTCAGACGTGTGCTCTTCCGATCTGAC	ACAGTGGT
U_rev_5	5'-CAAGCAGAAGACGGCATACGAGAT-AACGTGAT-GTGACTGGAGTTCAGACGTGTGCTCTTCCGATCTGAC	ATCACGTT
U_rev_6	5'-CAAGCAGAAGACGGCATACGAGAT-CGCTGATC-GTGACTGGAGTTCAGACGTGTGCTCTTCCGATCTGAC	GATCAGCG
U_rev_7	5'-CAAGCAGAAGACGGCATACGAGAT-CAGATCTG-GTGACTGGAGTTCAGACGTGTGCTCTTCCGATCTGAC	CAGATCTG
U_rev_8	5'-CAAGCAGAAGACGGCATACGAGAT-ATGCCTAA-GTGACTGGAGTTCAGACGTGTGCTCTTCCGATCTGAC	TTAGGCAT
U_rev_9	5'-CAAGCAGAAGACGGCATACGAGAT-CTGTAGCC-GTGACTGGAGTTCAGACGTGTGCTCTTCCGATCTGAC	GGCTACAG
U_rev_10	5'-CAAGCAGAAGACGGCATACGAGAT-AGTACAAG-GTGACTGGAGTTCAGACGTGTGCTCTTCCGATCTGAC	CTTGTACT
U_rev_11	5'-CAAGCAGAAGACGGCATACGAGAT-CATCAAGT-GTGACTGGAGTTCAGACGTGTGCTCTTCCGATCTGAC	ACTTGATG
U_rev_12	5'-CAAGCAGAAGACGGCATACGAGAT-AGTGGTCA-GTGACTGGAGTTCAGACGTGTGCTCTTCCGATCTGAC	TGACCACT
U_rev_13	5'-CAAGCAGAAGACGGCATACGAGAT-AACAACCA-GTGACTGGAGTTCAGACGTGTGCTCTTCCGATCTGAC	TGGTTGTT
U_rev_14	5'-CAAGCAGAAGACGGCATACGAGAT-AACCGAGA-GTGACTGGAGTTCAGACGTGTGCTCTTCCGATCTGAC	TCTCGGTT
U_rev_15	5'-CAAGCAGAAGACGGCATACGAGAT-AACGCTTA-GTGACTGGAGTTCAGACGTGTGCTCTTCCGATCTGAC	TAAGCGTT
U_rev_16	5'-CAAGCAGAAGACGGCATACGAGAT-AAGACGGA-GTGACTGGAGTTCAGACGTGTGCTCTTCCGATCTGAC	TCCGTCTT
U_rev_17	5'-CAAGCAGAAGACGGCATACGAGAT-AAGGTACA-GTGACTGGAGTTCAGACGTGTGCTCTTCCGATCTGAC	TGTACCTT
U_rev_18	5'-CAAGCAGAAGACGGCATACGAGAT-ACACAGAA-GTGACTGGAGTTCAGACGTGTGCTCTTCCGATCTGAC	TTCTGTGT
U_rev_19	5'-CAAGCAGAAGACGGCATACGAGAT-ACAGCAGA-GTGACTGGAGTTCAGACGTGTGCTCTTCCGATCTGAC	TCTGCTGT
U_rev_20	5'-CAAGCAGAAGACGGCATACGAGAT-ACCTCCAA-GTGACTGGAGTTCAGACGTGTGCTCTTCCGATCTGAC	TTGGAGGT
U_rev_21	5'-CAAGCAGAAGACGGCATACGAGAT-ACGCTCGA-GTGACTGGAGTTCAGACGTGTGCTCTTCCGATCTGAC	TCGAGCGT
U_rev_22	5'-CAAGCAGAAGACGGCATACGAGAT-ACGTATCA-GTGACTGGAGTTCAGACGTGTGCTCTTCCGATCTGAC	TGATACGT
U_rev_23	5'-CAAGCAGAAGACGGCATACGAGAT-ACTATGCA-GTGACTGGAGTTCAGACGTGTGCTCTTCCGATCTGAC	TGCATAGT
U_rev_24	5'-CAAGCAGAAGACGGCATACGAGAT-AGAGTCAA-GTGACTGGAGTTCAGACGTGTGCTCTTCCGATCTGAC	TTGACTCT
U_rev_25	5'-CAAGCAGAAGACGGCATACGAGAT-AGATCGCA-GTGACTGGAGTTCAGACGTGTGCTCTTCCGATCTGAC	TGCGATCT
U_rev_26	5'-CAAGCAGAAGACGGCATACGAGAT-AGCAGGAA-GTGACTGGAGTTCAGACGTGTGCTCTTCCGATCTGAC	TTCCTGCT
U_rev_27	5'-CAAGCAGAAGACGGCATACGAGAT-AGTCACTA-GTGACTGGAGTTCAGACGTGTGCTCTTCCGATCTGAC	TAGTGACT
U_rev_28	5'-CAAGCAGAAGACGGCATACGAGAT-ATCCTGTA-GTGACTGGAGTTCAGACGTGTGCTCTTCCGATCTGAC	TACAGGAT
U_rev_29	5'-CAAGCAGAAGACGGCATACGAGAT-ATTGAGGA-GTGACTGGAGTTCAGACGTGTGCTCTTCCGATCTGAC	TCCTCAAT
U_rev_30	5'-CAAGCAGAAGACGGCATACGAGAT-CAACCACA-GTGACTGGAGTTCAGACGTGTGCTCTTCCGATCTGAC	TGTGGTTG
U_rev_31	5'-CAAGCAGAAGACGGCATACGAGAT-CAAGACTA-GTGACTGGAGTTCAGACGTGTGCTCTTCCGATCTGAC	TAGTCTTG
U_rev_32	5'-CAAGCAGAAGACGGCATACGAGAT-CAATGGAA-GTGACTGGAGTTCAGACGTGTGCTCTTCCGATCTGAC	TTCCATTG
U_rev_33	5'-CAAGCAGAAGACGGCATACGAGAT-CACTTCGA-GTGACTGGAGTTCAGACGTGTGCTCTTCCGATCTGAC	TCGAAGTG
U_rev_34	5'-CAAGCAGAAGACGGCATACGAGAT-CAGCGTTA-GTGACTGGAGTTCAGACGTGTGCTCTTCCGATCTGAC	TAACGCTG
U_rev_35	5'-CAAGCAGAAGACGGCATACGAGAT-CATACCAA-GTGACTGGAGTTCAGACGTGTGCTCTTCCGATCTGAC	TTGGTATG
U_rev_36	5'-CAAGCAGAAGACGGCATACGAGAT-CCAGTTCA-GTGACTGGAGTTCAGACGTGTGCTCTTCCGATCTGAC	TGAACTGG
U_rev_37	5'-CAAGCAGAAGACGGCATACGAGAT-CCGAAGTA-GTGACTGGAGTTCAGACGTGTGCTCTTCCGATCTGAC	TACTTCGG
U_rev_38	5'-CAAGCAGAAGACGGCATACGAGAT-CCGTGAGA-GTGACTGGAGTTCAGACGTGTGCTCTTCCGATCTGAC	TCTCACGG
U_rev_39	5'-CAAGCAGAAGACGGCATACGAGAT-CCTCCTGA-GTGACTGGAGTTCAGACGTGTGCTCTTCCGATCTGAC	TCAGGAGG
U_rev_40	5'-CAAGCAGAAGACGGCATACGAGAT-CGAACTTA-GTGACTGGAGTTCAGACGTGTGCTCTTCCGATCTGAC	TAAGTTCG
U_rev_41	5'-CAAGCAGAAGACGGCATACGAGAT-CGACTGGA-GTGACTGGAGTTCAGACGTGTGCTCTTCCGATCTGAC	TCCAGTCG
U_rev_42	5'-CAAGCAGAAGACGGCATACGAGAT-CGCATACA-GTGACTGGAGTTCAGACGTGTGCTCTTCCGATCTGAC	TGTATGCG
U_rev_43	5'-CAAGCAGAAGACGGCATACGAGAT-CTCAATGA-GTGACTGGAGTTCAGACGTGTGCTCTTCCGATCTGAC	TCATTGAG
U_rev_44	5'-CAAGCAGAAGACGGCATACGAGAT-CTGAGCCA-GTGACTGGAGTTCAGACGTGTGCTCTTCCGATCTGAC	TGGCTCAG
U_rev_45	5'-CAAGCAGAAGACGGCATACGAGAT-CTGGCATA-GTGACTGGAGTTCAGACGTGTGCTCTTCCGATCTGAC	TATGCCAG
U_rev_46	5'-CAAGCAGAAGACGGCATACGAGAT-GAATCTGA-GTGACTGGAGTTCAGACGTGTGCTCTTCCGATCTGAC	TCAGATTC
U_rev_47	5'-CAAGCAGAAGACGGCATACGAGAT-GACTAGTA-GTGACTGGAGTTCAGACGTGTGCTCTTCCGATCTGAC	TACTAGTC
U_rev_48	5'-CAAGCAGAAGACGGCATACGAGAT-GAGCTGAA-GTGACTGGAGTTCAGACGTGTGCTCTTCCGATCTGAC	TTCAGCTC
U_rev_49	5'-CAAGCAGAAGACGGCATACGAGAT-GATAGACA-GTGACTGGAGTTCAGACGTGTGCTCTTCCGATCTGAC	TGTCTATC
U_rev_50	5'-CAAGCAGAAGACGGCATACGAGAT-GCCACATA-GTGACTGGAGTTCAGACGTGTGCTCTTCCGATCTGAC	TATGTGGC
U_rev_51	5'-CAAGCAGAAGACGGCATACGAGAT-GCGAGTAA-GTGACTGGAGTTCAGACGTGTGCTCTTCCGATCTGAC	TTACTCGC
U_rev_52	5'-CAAGCAGAAGACGGCATACGAGAT-GCTAACGA-GTGACTGGAGTTCAGACGTGTGCTCTTCCGATCTGAC	TCGTTAGC

U_rev_53	5'-CAAGCAGAAGACGGCATACGAGAT-GCTCGGTA-GTGACTGGAGTTCAGACGTGTGCTCTTCCGATCTGAC	TACCGAGC
U_rev_54	5'-CAAGCAGAAGACGGCATACGAGAT-GGAGAACA-GTGACTGGAGTTCAGACGTGTGCTCTTCCGATCTGAC	TGTTCTCC
U_rev_55	5'-CAAGCAGAAGACGGCATACGAGAT-GGTGCGAA-GTGACTGGAGTTCAGACGTGTGCTCTTCCGATCTGAC	TTCGCACC
U_rev_56	5'-CAAGCAGAAGACGGCATACGAGAT-GTACGCAA-GTGACTGGAGTTCAGACGTGTGCTCTTCCGATCTGAC	TTGCGTAC
U_rev_57	5'-CAAGCAGAAGACGGCATACGAGAT-GTCGTAGA-GTGACTGGAGTTCAGACGTGTGCTCTTCCGATCTGAC	TCTACGAC
U_rev_58	5'-CAAGCAGAAGACGGCATACGAGAT-GTCTGTCA-GTGACTGGAGTTCAGACGTGTGCTCTTCCGATCTGAC	TGACAGAC
U_rev_59	5'-CAAGCAGAAGACGGCATACGAGAT-GTGTTCTA-GTGACTGGAGTTCAGACGTGTGCTCTTCCGATCTGAC	TAGAACAC
U_rev_60	5'-CAAGCAGAAGACGGCATACGAGAT-TAGGATGA-GTGACTGGAGTTCAGACGTGTGCTCTTCCGATCTGAC	TCATCCTA
U_rev_61	5'-CAAGCAGAAGACGGCATACGAGAT-TATCAGCA-GTGACTGGAGTTCAGACGTGTGCTCTTCCGATCTGAC	TGCTGATA
U_rev_62	5'-CAAGCAGAAGACGGCATACGAGAT-TCCGTCTA-GTGACTGGAGTTCAGACGTGTGCTCTTCCGATCTGAC	TAGACGGA
U_rev_63	5'-CAAGCAGAAGACGGCATACGAGAT-TCTTCACA-GTGACTGGAGTTCAGACGTGTGCTCTTCCGATCTGAC	TGTGAAGA
U_rev_64	5'-CAAGCAGAAGACGGCATACGAGAT-TGAAGAGA-GTGACTGGAGTTCAGACGTGTGCTCTTCCGATCTGAC	TCTCTTCA
U_rev_65	5'-CAAGCAGAAGACGGCATACGAGAT-TGGAACAA-GTGACTGGAGTTCAGACGTGTGCTCTTCCGATCTGAC	TTGTTCCA
U_rev_66	5'-CAAGCAGAAGACGGCATACGAGAT-TGGCTTCA-GTGACTGGAGTTCAGACGTGTGCTCTTCCGATCTGAC	TGAAGCCA
U_rev_67	5'-CAAGCAGAAGACGGCATACGAGAT-TGGTGGTA-GTGACTGGAGTTCAGACGTGTGCTCTTCCGATCTGAC	TACCACCA
U_rev_68	5'-CAAGCAGAAGACGGCATACGAGAT-TTCACGCA-GTGACTGGAGTTCAGACGTGTGCTCTTCCGATCTGAC	TGCGTGAA
U_rev_69	5'-CAAGCAGAAGACGGCATACGAGAT-AACTCACC-GTGACTGGAGTTCAGACGTGTGCTCTTCCGATCTGAC	GGTGAGTT
U_rev_70	5'-CAAGCAGAAGACGGCATACGAGAT-AAGAGATC-GTGACTGGAGTTCAGACGTGTGCTCTTCCGATCTGAC	GATCTCTT
U_rev_71	5'-CAAGCAGAAGACGGCATACGAGAT-AAGGACAC-GTGACTGGAGTTCAGACGTGTGCTCTTCCGATCTGAC	GTGTCCTT
U_rev_72	5'-CAAGCAGAAGACGGCATACGAGAT-AATCCGTC-GTGACTGGAGTTCAGACGTGTGCTCTTCCGATCTGAC	GACGGATT
U_rev_73	5'-CAAGCAGAAGACGGCATACGAGAT-AATGTTGC-GTGACTGGAGTTCAGACGTGTGCTCTTCCGATCTGAC	GCAACATT
U_rev_74	5'-CAAGCAGAAGACGGCATACGAGAT-ACACGACC-GTGACTGGAGTTCAGACGTGTGCTCTTCCGATCTGAC	GGTCGTGT
U_rev_75	5'-CAAGCAGAAGACGGCATACGAGAT-ACAGATTC-GTGACTGGAGTTCAGACGTGTGCTCTTCCGATCTGAC	GAATCTGT
U_rev_76	5'-CAAGCAGAAGACGGCATACGAGAT-AGATGTAC-GTGACTGGAGTTCAGACGTGTGCTCTTCCGATCTGAC	GTACATCT
U_rev_77	5'-CAAGCAGAAGACGGCATACGAGAT-AGCACCTC-GTGACTGGAGTTCAGACGTGTGCTCTTCCGATCTGAC	GAGGTGCT
U_rev_78	5'-CAAGCAGAAGACGGCATACGAGAT-AGCCATGC-GTGACTGGAGTTCAGACGTGTGCTCTTCCGATCTGAC	GCATGGCT
U_rev_79	5'-CAAGCAGAAGACGGCATACGAGAT-AGGCTAAC-GTGACTGGAGTTCAGACGTGTGCTCTTCCGATCTGAC	GTTAGCCT
U_rev_80	5'-CAAGCAGAAGACGGCATACGAGAT-ATAGCGAC-GTGACTGGAGTTCAGACGTGTGCTCTTCCGATCTGAC	GTCGCTAT
U_rev_81	5'-CAAGCAGAAGACGGCATACGAGAT-ATCATTCC-GTGACTGGAGTTCAGACGTGTGCTCTTCCGATCTGAC	GGAATGAT
U_rev_82	5'-CAAGCAGAAGACGGCATACGAGAT-ATTGGCTC-GTGACTGGAGTTCAGACGTGTGCTCTTCCGATCTGAC	GAGCCAAT
U_rev_83	5'-CAAGCAGAAGACGGCATACGAGAT-CAAGGAGC-GTGACTGGAGTTCAGACGTGTGCTCTTCCGATCTGAC	GCTCCTTG
U_rev_84	5'-CAAGCAGAAGACGGCATACGAGAT-CACCTTAC-GTGACTGGAGTTCAGACGTGTGCTCTTCCGATCTGAC	GTAAGGTG
U_rev_85	5'-CAAGCAGAAGACGGCATACGAGAT-CCATCCTC-GTGACTGGAGTTCAGACGTGTGCTCTTCCGATCTGAC	GAGGATGG
U_rev_86	5'-CAAGCAGAAGACGGCATACGAGAT-CCGACAAC-GTGACTGGAGTTCAGACGTGTGCTCTTCCGATCTGAC	GTTGTCGG
U_rev_87	5'-CAAGCAGAAGACGGCATACGAGAT-CCTAATCC-GTGACTGGAGTTCAGACGTGTGCTCTTCCGATCTGAC	GGATTAGG
U_rev_88	5'-CAAGCAGAAGACGGCATACGAGAT-CCTCTATC-GTGACTGGAGTTCAGACGTGTGCTCTTCCGATCTGAC	GATAGAGG
U_rev_89	5'-CAAGCAGAAGACGGCATACGAGAT-CGACACAC-GTGACTGGAGTTCAGACGTGTGCTCTTCCGATCTGAC	GTGTGTCG
U_rev_90	5'-CAAGCAGAAGACGGCATACGAGAT-CGGATTGC-GTGACTGGAGTTCAGACGTGTGCTCTTCCGATCTGAC	GCAATCCG
U_rev_91	5'-CAAGCAGAAGACGGCATACGAGAT-CTAAGGTC-GTGACTGGAGTTCAGACGTGTGCTCTTCCGATCTGAC	GACCTTAG
U_rev_92	5'-CAAGCAGAAGACGGCATACGAGAT-GAACAGGC-GTGACTGGAGTTCAGACGTGTGCTCTTCCGATCTGAC	GCCTGTTC
U_rev_93	5'-CAAGCAGAAGACGGCATACGAGAT-GACAGTGC-GTGACTGGAGTTCAGACGTGTGCTCTTCCGATCTGAC	GCACTGTC
U_rev_94	5'-CAAGCAGAAGACGGCATACGAGAT-GAGTTAGC-GTGACTGGAGTTCAGACGTGTGCTCTTCCGATCTGAC	GCTAACTC
U_rev_95	5'-CAAGCAGAAGACGGCATACGAGAT-GATGAATC-GTGACTGGAGTTCAGACGTGTGCTCTTCCGATCTGAC	GATTCATC
U_rev_96	5'-CAAGCAGAAGACGGCATACGAGAT-GCCAAGAC-GTGACTGGAGTTCAGACGTGTGCTCTTCCGATCTGAC	GTCTTGGC

(B) Illumina sequencing reaction

ID	Sequence	Barcode
Read_1	5'-ACACTCTTTCCCTACACGACGCTCTTCCGATCTCTG	NA
Read_2	5'-GTGACTGGAGTTCAGACGTGTGCTCTTCCGATCTGAC	NA
